# Power dynamics and intersectoral collaboration for health in low- and middle-income countries: a realist review

**DOI:** 10.1093/heapol/czaf022

**Published:** 2025-04-05

**Authors:** Praveenkumar Aivalli, Sara Dada, Brynne Gilmore, Prashanth Nuggehalli Srinivas, Aoife De Brún

**Affiliations:** UCD Centre for Interdisciplinary Research Education and Innovation in Health Systems (UCD IRIS Centre), School of Nursing Midwifery and Health Systems, University College Dublin, Dublin 4, Ireland; School of Nursing Midwifery and Health Systems, University College Dublin, Dublin 4, Ireland; UCD Centre for Interdisciplinary Research Education and Innovation in Health Systems (UCD IRIS Centre), School of Nursing Midwifery and Health Systems, University College Dublin, Dublin 4, Ireland; School of Nursing Midwifery and Health Systems, University College Dublin, Dublin 4, Ireland; UCD Centre for Interdisciplinary Research Education and Innovation in Health Systems (UCD IRIS Centre), School of Nursing Midwifery and Health Systems, University College Dublin, Dublin 4, Ireland; School of Nursing Midwifery and Health Systems, University College Dublin, Dublin 4, Ireland; Institute of Public Health, No. 250, 2nd C Main, 2nd Cross, Girinagar 1st Phase, Bangalore, Karnataka 560070, India; UCD Centre for Interdisciplinary Research Education and Innovation in Health Systems (UCD IRIS Centre), School of Nursing Midwifery and Health Systems, University College Dublin, Dublin 4, Ireland; School of Nursing Midwifery and Health Systems, University College Dublin, Dublin 4, Ireland

**Keywords:** intersectoral collaboration, power dynamics, implementation, LMICs, realist, realist review

## Abstract

Intersectoral collaboration (ISC) is a critical strategy in global health for addressing complex challenges requiring multi-sectoral engagement. Although studies examined ISC in low- and middle-income countries (LMICs), gaps remain in understanding how power dynamics between stakeholders influence the effectiveness of ISC in these settings. This realist synthesis examines how, why, for whom, under what context, and to what extent power dynamics shape ISC in LMIC health programmes and policies, offering insights crucial for improving health policy implementation. Five initial programme theories were developed through a scoping review, document analysis, and qualitative study. A systematic search of Medline, Embase, CINAHL, Web of Science, and grey literature (2012–23) yielded 2850 records, with 23 included after screening. This period was chosen to capture contemporary shifts in ISC, following the 2012 UN Political Declaration on NCDs and the WHO’s 2013 Health in All Policies (HiAP) framework, which strengthened multi-sectoral governance in LMICs. It also builds on prior reviews, ensuring an up-to-date synthesis of power dynamics in ISC. Data were synthesized using the context–mechanism–outcome framework, generating demi-regularities to refine programme theories (PTs). Findings reveal that power imbalances frequently manifest through hierarchical governance structures, resource disparities, and historical inequities, shaping ISC outcomes. Six refined PTs highlight: (i) inclusive policy development processes mitigate power asymmetries but require intentional facilitation to prevent marginalization of less dominant sectors. (ii) Leadership commitment and shared goal alignment enhance collaboration, yet competing institutional priorities often reinforce power struggles. (iii) Equitable resource allocation acts as both a catalyst for trust and a source of conflict, with donor influence exacerbating dependency dynamics. (iv) Hierarchical communication norms in LMICs undermine transparency, though informal interpersonal networks can circumvent bureaucratic barriers. (v) Ambiguity in roles and mandates amplifies power vacuums, enabling dominant actors to disproportionately influence agendas. Additionally, a sixth PT emerged: (vi) sustained interpersonal relationships counterbalance structural power imbalances, fostering accountability and adaptive problem-solving. These findings demonstrate that power dynamics in ISC within LMICs are mediated by both structural factors (e.g. funding models and institutional hierarchies) and relational mechanisms (e.g. trust and negotiation). Successful collaboration hinges on recognizing and addressing these dual dimensions of power. This synthesis advances the theoretical and practical understanding of ISC, offering policymakers actionable insights to navigate power-related challenges in intersectoral health initiatives.

Key messages

- Power imbalances in intersectoral collaborations in LMICs are entrenched by donor dependency and hierarchical governance; equitable partnerships require binding agreements on resource transparency and mechanisms to counterbalance donor influence on agendas.
- Inclusive policy processes fail without intentional facilitation—structurally marginalized sectors (e.g. local non-governmental organization) require dedicated support to ensure their input shapes decisions.
- Role ambiguity creates power vacuums exploited by dominant actors—collaborative governance mandates must clarify sectoral responsibilities and enforce accountability through joint monitoring frameworks.
- Informal interpersonal networks can often achieve what formal hierarchies cannot—policymakers should incentivize trust-building activities (e.g. cross-sector staff rotations and shared platforms for grassroots stakeholders) to circumvent bureaucratic barriers.
- Donor-driven funding models perpetuate inequity—transitioning to pooled, multi-sectoral financing with LMIC-led prioritization reduces power asymmetries and fosters locally sustainable collaboration.
- Leadership alignment alone cannot resolve institutional power struggles—shared goals must be paired with conflict mediation protocols and penalties for noncompliance to prevent high-resource sectors from dominating agendas.

## Introduction

Intersectoral collaboration (ISC) is widely recognized as a fundamental strategy in global health due to its holistic approach to addressing complex health challenges. This recognition dates back to the Alma Ata Declaration of 1978, which underscored the necessity of primary healthcare and intersectoral action to achieve ‘Health for All’ (World Health Organization 1978). The OttawaModern Classics of Leadership Charter for Health Promotion (1986) further reinforced the importance of ISC by advocating for healthy public policies across sectors, fostering supportive environments, and strengthening community action to improve public health (WHO 1986).

The Agenda 2030 for Sustainable Development Goals solidified ISC’s centrality, particularly in Goal 3, which seeks to ensure healthy lives and well-being for all. Achieving this goal necessitates cross-sectoral engagement in areas such as education, gender equality, and clean water and sanitation—each integral to advancing health outcomes (Colglazier 2015). The World Health Organization (WHO) has defined ISC as follows:

“A recognised relationship between part or parts of the health sector with part or parts of another sector formed to take action on an issue to achieve health outcomes in a way that is more effective, efficient, or sustainable than could be achieved by the health sector acting alone”. (WHO 1997, p.3)

The conceptual landscape surrounding ISC is characterized by terminological plurality, with terms including ‘intersectoral action’, ‘multi-sectoral collaboration’, and ‘cross-sector collaboration’ employed interchangeably in academic and policy discourse (Chircop et al. 2015, Colglazier 2015, Asaaga et al. 2021). While ISC is widely regarded as an essential mechanism for tackling complex societal challenges, its operationalization remains ambiguous, partly due to inconsistent conceptualizations of ‘collaboration’ across disciplines (Emerson et al. 2012, Chircop et al. 2015). Scholars have attempted to clarify these conceptual foundations using theoretical frameworks such as collaborative governance and network governance, which emphasize relational dynamics, power structures, and institutional interdependencies (Emerson et al. 2012, Belrhiti et al. 2024). However, persistent gaps remain in understanding how these theoretical constructs manifest in low- and middle-income countries (LMICs), particularly within health systems and public policy contexts.

Systematic reviews examining ISC governance in LMICs have identified structural barriers including fragmented institutional coordination, sectoral silos, and political economy constraints (Bennett et al. 2018, Glandon et al. 2019, Ssennyonjo et al. 2021). While these analyses provide critical insights into formal governance arrangements, they often prioritize institutional architectures over the informal power relations that fundamentally shape collaborative processes (Glandon et al. 2018, Belrhiti et al. 2024). For instance, Bennett et al. (2018) highlight the technical challenges of aligning sectoral priorities but pay limited attention to how asymmetrical power dynamics between stakeholders influence agenda-setting and resource allocation. Similarly, Ssennyonjo et al. (2021) apply social science theories to intragovernmental coordination yet acknowledge the need for deeper empirical exploration of power imbalances in multi-sectoral policy-making.

Recent advancements in health policy and systems research have begun addressing these gaps by integrating political economy lenses and participatory methodologies (Parashar et al. 2020, Dakyaga et al. 2023, BelrhitI et al. 2024). For example, Belrhiti et al. (2024) demonstrate how power asymmetries—rooted in resource disparities, epistemic hierarchies, and institutional mandates—undermine collaborative decision-making in healthcare networks. Their findings resonate with broader critiques of collaborative governance, where theoretical models of ‘equitable partnership’ often fail to account for hierarchical realities in LMIC contexts (Glandon et al. 2019, Okeyo et al. 2020b). While these insights have deepened our understanding, critical questions remain regarding how power operates in everyday ISC practices, particularly during policy implementation, where bureaucratic politics and community-level discretion intersect (Parashar et al. 2020, Dakyaga et al. 2023).

This synthesis builds on prior work by interrogating the micro-dynamics of power within ISC frameworks, shifting the focus from static institutional analyses to how relational tensions and negotiation processes shape health policy outcomes. Previous reviews have identified coordination challenges (Bennett et al. 2018, Glandon et al. 2019), yet they primarily emphasized macro-level governance structures rather than the contextual realities of power negotiation among frontline actors. Additionally, while organizational theories offer robust frameworks for analysing collaboration—such as relational contracts (Nabatchi and Emerson 2021), trust-building mechanisms (Emerson et al. 2012), and network resilience (Provan and Kenis 2008)—their application to LMIC health systems remains underdeveloped. By synthesizing contemporary evidence on power dynamics (Belrhiti et al. 2024) with grounded insights from LMIC policy contexts, this review contributes a nuanced understanding of how ISC can be reimagined through equity-centred governance frameworks.

### Theoretical Bases for ISC

A diverse range of theoretical perspectives informs ISC, offering analytical tools to examine how sectors interact, share resources, and navigate complex governance environments. Institutional theory (Peters 2022) provides a structural lens, emphasizing how formal rules, norms, and organizational cultures shape collaboration. However, it has been critiqued for its limited attention to the fluid and negotiated nature of cross-sectoral interactions, which are often influenced by informal power dynamics and relational dependencies (Scott 2005).

Resource dependence theory (Pfeffer and Salancik 2015) shifts the focus to how organizations engage in strategic exchanges to manage resource constraints. While this perspective is useful in understanding power asymmetries in ISC, it tends to assume predictable dependency patterns, underestimating the contextual variations in resource flows across sectors with differing institutional mandates and political priorities. In LMICs, where intersectoral initiatives often operate amid resource scarcity and donor-driven agendas, the theory’s assumptions require critical interrogation.

Collaborative governance theory (Ansell and Gash 2008) is particularly relevant to ISC, as it highlights mechanisms such as shared motivation, trust-building, and the capacity for joint action. However, its emphasis on consensual decision-making does not always align with the hierarchical and resource-constrained realities of LMIC settings (Emerson et al. 2012). The assumption that collaboration emerges through deliberative processes overlooks underlying power struggles, particularly in contexts where dominant sectors exert disproportionate influence over policy-making and resource allocation.

Given the adaptive and nonlinear nature of ISC, complexity theory (Byrne 2002) provides valuable insights into the emergent properties of collaboration, illustrating how small shifts in actor relationships can lead to systemic changes. However, its focus on macro-level system dynamics often lacks the granularity needed for actionable policy insights (Plsek and Greenhalgh 2001). Similarly, social network theory (Liu et al. 2017) enhances our understanding of stakeholder interactions, power relations, and influence diffusion but tends to prioritize network structures over the micro-level processes that sustain or undermine collaboration.

Boundary spanning theory (Schotter et al. 2017) offers a complementary perspective by examining individual actors who mediate cross-sectoral interactions. It underscores the role of brokers, intermediaries, and champions in navigating institutional divides. However, while boundary spanners can facilitate coordination, their effectiveness is often constrained by structural hierarchies and sectoral mandates, limiting their ability to foster equitable partnerships across sectors.

Rather than adopting any single theoretical lens, this study integrates insights from these perspectives to develop a more nuanced understanding of power dynamics in ISC. By synthesizing institutional structures, resource dependencies, governance mechanisms, and relational interactions, this approach bridges existing theoretical perspectives while critically assessing their applicability to varied governance contexts, particularly in LMICs. This synthesis allows for a more contextually grounded analysis of power, moving beyond abstract conceptualizations to examine how collaboration unfolds in practice.

### Power dynamics

Power dynamics in health systems are shaped by the interactions among various stakeholders, including policymakers, healthcare providers, communities, international organizations, and nongovernmental organizations (NGOs) (Gilson 2003, Erasmus and Gilson 2008, Walt et al. 2008). These dynamics influence decision-making processes, resource allocation, and policy implementation, often determining whose interests are prioritized. Lukes’ (2005) three-dimensional framework conceptualizes power beyond overt decision-making, incorporating non-decisionmaking, which involves the suppression of alternative voices, and ideological power, which operates by shaping beliefs and preferences. This tripartite model highlights power’s structural, relational, and discursive dimensions, revealing how it is embedded within institutional arrangements and everyday practices.

Power in ISC is inherently complex, shaped by both formal structures and informal negotiations. Systems theory provides further insights into power’s nonlinearity, emergence, and path dependency (Gilson et al. 2017). Nonlinearity underscores that power relations do not follow predictable trajectories; small shifts in influence can lead to disproportionate changes in policy outcomes. Emergence highlights how macro-level power structures, such as global health governance frameworks, arise from micro-level interactions (Adam and De Savigny 2012). Path dependency reflects how historical inequities, including colonial legacies and entrenched sectoral hierarchies, continue to shape contemporary policy-making (Frenk and Gómez-Dantés 2018). These perspectives emphasize that power is not merely a static resource but is continuously reproduced through feedback loops, contested narratives, and institutional adaptations (ADAM and De Savigny 2012, Gilson et al. 2017).

In ISC, power asymmetries manifest through hierarchical decision-making, resource disparities, and sectoral dominance. Network governance theories argue that power in multi-sector collaborations is not simply additive but emergent, shaped by actors with varying levels of authority, legitimacy, and knowledge (Provan and Kenis 2008, Klijn and Koppenjan 2015). However, structural inequities often privilege well-resourced sectors, such as finance and health, while marginalizing local governments and civil society actors (Huxham and Vangen 2013, Cairney and Oliver 2020). This reinforces patterns of policy capture, where dominant stakeholders steer ISC agendas in ways that may not align with community priorities.

To navigate these challenges, ISC requires adaptive governance approaches that acknowledge the contextual fluidity of power (Brinkerhoff 2002b, Belrhiti et al. 2024). Political economy perspectives further illuminate how global actors, donor priorities, and national policies interact to influence local decision-making structures (Sheikh et al. 2011, ADAM and De Savigny 2012). For example, international funding agencies may impose technical priorities that override local expertise, reinforcing epistemic hierarchies and limiting bottom-up participation (Molyneux et al. 2012).

In LMICs, understanding power dynamics in ISC necessitates examining how structural inequities influence everyday governance practices. For instance, a donor-funded nutrition programme may unintentionally deepen power imbalances if its design prioritizes technical expertise over local knowledge (Molyneux et al. 2012). Similarly, bureaucratic incentives and political affiliations can shape who has access to decision-making spaces, further entrenching disparities (Sheikh et al. 2011, Gilson et al. 2017). Recognizing these dynamics allows for more context-sensitive strategies that promote balanced power relations, such as embedding neutral facilitation mechanisms, ensuring participatory budgeting, and fostering multi-level accountability (Ansell and Gash 2008, Provan and Kenis 2008, Okeyo et al. 2020a).

The notion of ‘balanced power dynamics’ distinguishes itself from ‘equitable power distribution’ by prioritizing relational parity—ensuring no single actor dominates decision-making—rather than pursuing uniform influence across sectors. Achieving balance necessitates context-sensitive strategies to mitigate asymmetries, such as instituting neutral facilitation mechanisms (e.g. third-party mediators) to amplify marginalized voices, embedding transparency in resource allocation through participatory budgeting, enforcing accountability via independent oversight bodies, and strengthening capacity among under-resourced stakeholders through targeted training (Ansell and Gash 2008, Provan and Kenis 2008, Klijn and Koppenjan 2015, Okeyo et al. 2020b). For instance, Kenya’s integration of local community leaders into national health committees exemplifies how iterative negotiation and adaptive governance can reduce hierarchical control while fostering dynamic equilibrium (Okeyo et al. 2020b). Crucially, balance is not static parity but a continuous process of recalibration, demanding flexibility to address shifting power relations and sectoral interdependencies (Brinkerhoff 2002b, Belrhiti et al. 2024).

This synthesis adopts an organizational and institutional lens to power dynamics, prioritizing frameworks that elucidate how stakeholders negotiate authority, resources, and agendas within ISC. While acknowledging broader conceptualizations. Achieving balanced power dynamics does not necessitate equal influence across sectors but rather ensures that no single actor monopolizes decision-making. This requires continuous recalibration of power relations, responsive governance structures, and iterative negotiation to accommodate shifting sectoral interdependencies (Brinkerhoff 2002a, Belrhiti et al. 2024). By incorporating political economy insights, institutional perspectives, and systems-based approaches, this study seeks to advance a more nuanced understanding of power dynamics in ISC, bridging theory with the realities of policy-making in LMICs.

Given the complexity and fluidity of power in ISC, its exploration requires multiple analytical lenses that move beyond institutional and structural analyses. Power is not merely about formal authority or resource control but is relational, negotiated, and contingent on historical, political, and contextual factors. Understanding these dimensions necessitates methodological approaches that account for underlying mechanisms and conditional influences, rather than assuming linear relationships between governance structures and outcomes. A realist approach, which focuses on how and why power dynamics manifest differently across contexts, offers a promising way forward in disentangling these complexities and will be explored in the following section.

### Rationale of realist synthesis

ISC is inherently complex, shaped by power dynamics, institutional structures, and contextual contingencies. Traditional evaluative approaches, which assume linear cause–effect relationships (successional causation), fail to capture the interactive and evolving nature of ISC (Astbury and Leeuw 2010). Instead, ISC requires an approach that examines generative causation, focusing on how and why specific mechanisms—such as trust-building, resource negotiation, or bureaucratic discretion—produce outcomes under particular conditions (Marchal et al. 2012). A realist synthesis is uniquely suited to this task, as it explains not just whether an intervention works, but how, why, for whom, and under what conditions it succeeds or fails (Pawson et al. 2005, Wong et al. 2013b).

At the heart of realist methodology is the context–mechanism–outcome (CMO) configuration (CMOC), which provides a structured framework for analysing complex interventions. Context (C) refers to the environmental, institutional, and sociocultural conditions in which ISC operates—such as resource constraints, hierarchical governance structures, or historical inequities (Adam and De Savigny 2012). Mechanisms (M) are the underlying processes, responses, or reasoning triggered in specific contexts, shaping how actors navigate collaboration (Pawson 2006). Outcomes (O) are not merely the direct results of ISC initiatives but emerge from the interaction between context and mechanisms. For example, a participatory decision-making process (M) may empower local health workers (O) in contexts where governance structures are decentralized (C) but may fail in top-down, bureaucratic environments where power is concentrated.

### A realist synthesis was chosen for three key reasons

First, ISC is fundamentally dynamic, shaped by the interactions of multiple actors, institutions, and systems over time. Traditional systematic reviews, which often prioritize aggregating evidence to determine intervention effectiveness, are ill-equipped to unravel the underlying mechanisms driving collaboration (Mondal et al. 2021). ISC outcomes result from iterative processes involving negotiation, power struggles, and adaptation, requiring an approach that captures these emergent patterns.

Second, the existing literature on ISC in LMICs lacks robust theoretical frameworks that explain how power dynamics influence implementation (Emerson and Nabatchi 2015, Sriram et al. 2018). While studies discuss institutional arrangements and policy mandates, they often fail to account for the ways in which power is exercised, contested, and reconfigured. Realist synthesis addresses this gap by generating empirically grounded, testable explanations for how power relations shape ISC effectiveness.

This synthesis engages with key theoretical frameworks, including institutional theory, network governance, and participatory governance models. While these theories explain macro-level structures, they often treat governance as static, overlooking how power operates differently across contexts. Realist synthesis complements these perspectives by examining how specific mechanisms—such as legitimacy, trust-building, or informal bargaining—are activated or constrained depending on contextual conditions (Greenhalgh et al. 2017). A realist lens helps unpack how these historical and political contexts interact with mechanisms such as trust, negotiation, and coalition-building, producing different outcomes across settings (Brinkerhoff 2002a, Belrhiti et al. 2024).

For instance, a donor-funded health initiative may inadvertently reinforce power asymmetries (O) if its design (M) prioritizes technical expertise over local knowledge in contexts (C) where epistemic hierarchies prevail (Molyneux et al. 2012). Similarly, bureaucratic discretion (M) may empower local implementers (O) in decentralized governance structures (C) but may be constrained in centralized systems where policy control is highly top-down. These examples illustrate why ISC cannot be understood solely through institutional mandates or policy frameworks—it requires an examination of underlying mechanisms within specific contexts.

By leveraging realist synthesis, this study develops context-sensitive theories of change, offering insights for practitioners, policymakers, and researchers. This approach does not merely apply existing theories but constructs empirically grounded explanations that inform adaptive, equity-focused collaboration models.

The research question for this realist synthesis is: what are the causal explanations for how, why, and for whom power dynamics influence ISC during the implementation of ISC health programs and policies in LMICs?

## Materials and methods

The review adhered to the Realist and Meta-narrative Evidence Syntheses: Evolving Standards (RAMESES) publication standards (Wong et al. 2016). The review process involved following iterative steps.

### Defining review scope

The review began by defining its scope and locating existing theories related to power dynamics in ISC. To ground the analysis in empirical evidence, a small-scale qualitative study was conducted in Assam, India, focusing on programme implementers’ perspectives on ISC and the role of power within ISC (Aivalli et al. 2024). The study employed semi-structured interviews with 24 participants, including health administrators, NGO representatives, and frontline workers involved in maternal and child health ISC initiatives. Participants were purposively sampled to capture diverse roles and hierarchies. Interviews explored (i) experiences of collaboration, (ii) perceived power imbalances, and (iii) contextual barriers or facilitators to equitable decision-making. Data were transcribed, anonymised, and analysed using thematic analysis (Braun and Clarke 2006), with codes iteratively refined to identify patterns in power-related challenges (e.g. resource control and institutional mandates) and coping strategies (e.g. negotiation and coalition-building).

Findings from Assam were systematically compared with evidence from peer-reviewed studies on ISC in LMICs. To ensure rigour, a cross-case comparative approach (Bartlett and Vavrus 2017) was applied: themes from Assam (e.g. ‘asymmetrical resource allocation stifling local agency’) were juxtaposed with analogous or contrasting findings from other LMIC contexts (e.g. decentralized governance enabling flexibility in Ethiopia; (Garcia and Rajkumar 2008). This process highlighted recurring mechanisms (e.g. trust-building as a mediator of power imbalances) and context-specific variables (e.g. political stability) shaping ISC outcomes.

Additionally, the selection of theories for the development of initial programme theories (IPTs) followed a structured and iterative process to minimize selection bias, as recommended by Booth et al. (2020). IPTs are hypotheses developed during the initial stages of realist evaluation that outline how, why, and under what circumstances a programme or intervention is expected to produce specific outcomes. IPTs propose the underlying mechanisms—factors that generate change—and the contextual conditions necessary for these mechanisms to operate (Pawson and Tilley 1997). A realist-informed scoping review was conducted, employing a systematic search strategy across multiple academic databases to identify theoretical frameworks relevant to ISC. Theories were mapped against key ISC constructs, such as power dynamics, resource exchange, and governance structures, to assess their explanatory relevance. Additionally, expert consultations and iterative engagement with stakeholders ensured the inclusion of theories that aligned with both empirical evidence and practical insights from the field. However, limitations of these theoretical frameworks were identified during IPT development. Institutional theory, for instance, primarily focuses on organizational structures and norms but may underplay the dynamic and negotiated aspects of ISC (Scott 2005).

Resource dependence theory highlights resource exchanges but does not fully account for variations in power relations across different governance contexts (Pfeffer and Salancik 2015). While collaborative governance theory provides insights into shared motivation and trust-building (Ansell and Gash 2008), its applicability in hierarchical and resource-constrained settings remains a challenge (Emerson et al. 2012). To address these limitations, ‘folk theories’ emerging from qualitative insights—such as implementers’ lived experiences, tacit knowledge, and local governance dynamics—were systematically merged with global ISC literature. This integration was achieved through a scoping review, ensuring methodological rigour in identifying and synthesizing both formal theories and context-specific explanatory insights. By making these processes explicit, the study strengthens its theoretical foundation, ensuring that IPT development is both empirically grounded and analytically robust. This comparative synthesis informed the development of five IPTs, which sought to explain how and why power asymmetries shape collaborative outcomes in different contexts. By integrating empirical evidence with broader theoretical insights, the IPTs offer a nuanced understanding of the mechanisms driving effective or constrained ISC.

### Searching databases for evidence

Systematic searches were conducted in databases such as Medline, Embase, CINAHL, and Web of Science, as well as in Google and Google Scholar. The search included articles published from 1 January 2012 to 31 May 2023. Bibliographies of relevant articles were also searched to ensure a comprehensive review.

### Selection and appraisal of evidence

The lead author (P.A.) developed the inclusion and exclusion criteria, which were reviewed and agreed upon by the research team. Inclusion criteria were set to include peer-reviewed empirical studies that explored the aspects of power dynamics, such as power relations, hierarchy, psychological safety, communication, interpersonal relations, recognition, role clarity, role conflict, interpersonal influence, and dominance. To be included, articles needed to provide detailed information on how these factors interact within specific CMOCs. Both intended and unintended outcomes of power dynamics in ISC were considered. The exclusion criteria ruled out research that did not involve intersectoral or cross-sectoral collaboration, articles that only briefly mentioned ISC without explaining how power dynamics influenced it, studies focused on just one sector without discussing interactions with others, research conducted outside of LMICs, and previous systematic, literature, narrative, and realist reviews.

Selected studies were then screened for relevance, rigour, and richness, using Covidence software to manage the review process (Covidence 2014). The screening process involved an initial review by title and abstract, followed by a full-text assessment, performed by the lead author and a second researcher. Any disagreements were resolved through discussion between the researchers, with unresolved issues decided by a third member of the research team. The assessment focused on the relevance, richness, and rigour of the sources, evaluating the extent to which they provided detailed descriptions of their methods and the generalizability and trustworthiness of their findings based on those methods (Dada et al. 2023).

In realist synthesis, the unit of analysis is not the entirety of a study but the evidentiary fragments within the study (Wong et al. 2014). While the rigour of data in traditional systematic reviews is often based on the plausibility of the methods through which the data were generated (Wong et al. 2013, Dada et al. 2023), in realist synthesis, data could be drawn from any part of a paper, not just the results section (Jagosh 2019). The most important decision regarding data quality was the contribution each paper could make to building and refining the programme theory (PT), usually stemming from the ‘pieces’ of data rather than the entire body of the paper (Pawson 2006).

Rigour in realist synthesis refers to the credibility, plausibility, and trustworthiness of the methods used to generate data or a theory and depends on two criteria: trustworthiness (how plausible and reliable the methods used to obtain data are) and coherence (whether the data are consistent and logical with explanatory breadth) (Pawson 2006, Wong 2018). To ensure the relevance and rigour of the studies, they were classified into primary contributors (Erasmus and Gilson 2008, Mwisongo et al. 2016, Kamugumya and Olivier 2016, Janse van Rensburg et al. 2018, Aunger et al. 2021, Asaaga et al. 2021, Mondal et al. 2021, Ssennyonjo et al. 2022, Such et al. 2022, Etiaba et al. 2023, Nyawira et al. 2023) and secondary contributors (Buffardi et al. 2012, Kielmann et al. 2014, Okma et al. 2016, Kim et al. 2017, Khayatzadeh-Mahani et al. 2019, Balane et al. 2020, Okeyo et al. 2021, Yasobant et al. 2021, Bakhtiari et al. 2022, Male et al. 2022, Mondal et al. 2022, Pathak et al. 2022, Wagner et al. 2022, Chiari et al. 2023).

Primary contributors were studies that directly supported the study’s goals and were conducted in LMICs. Even if these studies did not fully explain ISC, they were included if they discussed power dynamics affecting ISC. Secondary contributors were studies that, while not as directly aligned with the study’s main goals, still provided useful insights into how mechanisms might work in the context of LMICs. Additionally, studies from nonhospital settings with similar contexts were also included as secondary contributors.

### Extraction of data

Data were extracted from the selected articles, focusing on CMOCs. The data were organized and coded using NVivo software (NVivo 2002), employing both inductive and deductive approaches. Deductive coding was informed by prespecified topics derived from the review’s conceptual framework and research questions, including power dynamics (e.g. decision-making hierarchies and veto authority), collaboration structures (e.g. formal agreements and accountability mechanisms), and contextual factors (e.g. political stability and donor influence). These codes aligned with established theories, such as institutional theory’s emphasis on norms and actor–network theory’s focus on stakeholder alliances, ensuring the analysis remained grounded in existing scholarship. Inductive coding, conversely, allowed novel themes to emerge organically from the data. For example, unanticipated dynamics like informal negotiation practices (e.g. frontline workers circumventing bureaucratic protocols to secure resources) and community-led resistance (e.g. grassroots advocacy to counter power imbalances) were identified, enriching the analysis with context-specific agency and adaptive strategies absent from prior frameworks.

The coding process in NVivo was closely integrated with a structured data abstraction form that documented study characteristics (e.g. country, sector, and intervention type) and preliminary CMOCs. Deductive codes populated predefined fields in the form, such as ‘mechanism: resource control’ or ‘context: decentralized governance’, while inductive codes generated new nodes (e.g. ‘mechanism: covert bargaining’). NVivo’s matrix coding queries enabled cross-tabulation of deductive and inductive codes, revealing patterns such as tensions between formal accountability mechanisms (deductive) and informal negotiation (inductive) in rigid bureaucratic contexts.

This process aimed to uncover the generative mechanisms at play in different contexts. A data extraction form was developed based on the IPTs and the synthesis aims, with iterative refinements throughout the process. During data extraction, each paper’s characteristics were recorded, including bibliographic details (title, author, journal, and year of publication); study type and design; target population, intervention, and type of programme (Delany-Crowe et al. 2019); generative causation in the form of contexts (Cs), mechanisms (Ms), and outcomes (Os) (Pawson and Tilley 1997); and notes/observations on the paper. Extracted data also included settings, power aspects in ISC, impacts on ISC health policy implementation, models or theoretical frameworks informing power dynamics, and CMOCs.

Evidence to confirm, refute, or refine the IPTs was documented alongside notes on decision-making processes and linkages to other IPTs. Two reviewers independently extracted and compared an initial sample of documents, and a small sample of data extraction forms was shared with other coauthors for feedback. Relevant data elements were used to test and refine the PT, re-examining sources to capture missed data. The goal was to reach theoretical saturation, prioritizing literature with primary relevance to the research questions (Wong et al. 2014, Jagosh 2020). To ensure transparency and rigour in the CMO analysis, Supplementary File 1 has been provided, including data extraction tables, coding frameworks, and iterative refinements of PTs. These documents enable reproducibility by demonstrating how data sources informed the refinement of CMOCs at each analytical stage.

### Analysis and synthesis

The extracted data were synthesized to form CMOCs. In this process, ‘Context’ refers to the specific conditions in which ISC takes place (e.g. hierarchical structures, resource constraints, or policy mandates), ‘Mechanism’ captures the underlying drivers or triggers that influence ISC (e.g. trust-building, negotiation, or bureaucratic authority), and ‘Outcome’ denotes the resulting effects of these interactions on programme implementation and effectiveness. These CMOCs are organized into demi-regularities. Demi-regularities refer to recurring but contingent patterns of social or behavioural phenomena that emerge under specific conditions, reflecting underlying causal mechanisms without deterministic predictability. Unlike universal laws in positivist traditions, demi-regularities in realist research acknowledge the complexity and context-dependence of social systems, wherein mechanisms may generate similar but not identical outcomes across different settings (Pawson 2006). These patterns serve as empirical signatures of causal processes, guiding researchers in identifying how and when particular mechanisms are activated or constrained by structural, institutional, and relational contexts. By recognizing demi-regularities, realist scholars can move beyond simple cause–effect relationships and instead develop nuanced explanations of what works, for whom, and under what conditions (Westhorp 2018).

This synthesis aimed to explain how power dynamics operate in different contexts and influence the outcomes of ISC health programmes. Data analysis techniques used in realist synthesizing include reconciling, situating, adjudicating, juxtaposing, and consolidating (Vareilles et al. 2017). In this realist synthesis, the data were employed to situate the operational mechanisms within the context of ISC in LMIC settings. This involved juxtaposing new mechanisms to enhance and propose a refined PT to explain the impact of power dynamics on the implementation of ISC health policies in these settings.

### Refine PT

The synthesis and analysis of data led to the refinement of the IPTs. The IPTs were iteratively adjusted based on evidence extracted from the data through a realist synthesis process. This involved systematically comparing findings with theoretical insights, identifying patterns of how power dynamics influenced collaboration, and modifying the IPTs to better reflect the generative causation observed in the data. For example, if initial assumptions suggested that formal governance structures were essential for ISC, but the data revealed that informal relationships played a stronger role, the IPT was revised to account for these emergent mechanisms. Through this iterative process, the IPTs evolved into a refined PT that more accurately explains the conditions under which power dynamics facilitate or hinder ISC effectiveness. The refined PTs were then validated and prepared for further testing in real-world settings.

A study protocol, including the development of IPTs, search strategy, inclusion/exclusion criteria, and associated steps in the process was registered at PROSPERO (protocol registration no. PROSPERO 2023 CRD42023460813) and a preprint was published (Aivalli et al. 2023).

## Results

**Figure 1.**
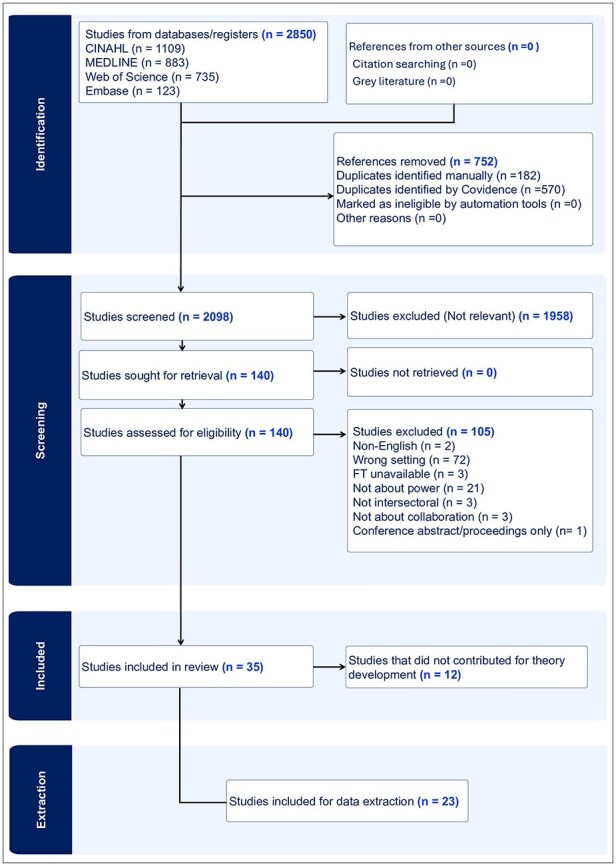
A detailed systematic review illustrated through the PRISMA flow diagram. PRISMA, Preferred Reporting Items for Systematic Reviews and Meta-Analysis.

Upon searching, a total of 2850 studies were identified from four databases: CINAHL (1109), MEDLINE (883), Web of Science (735), and Embase (123), with no additional references from other sources. After removing 752 duplicates identified by Covidence, 2098 studies were screened based on titles and abstracts, leading to the exclusion of 1958 studies. The remaining 140 studies were sought for full-text retrieval. Upon full-text assessment, 105 studies were excluded for reasons such as non-English language (2), wrong setting (72), full-text unavailability (3), irrelevance to power (21), lack of multisector focus (3), irrelevance to collaboration (3), and being conference abstracts only (1). Ultimately, 35 studies were included in the review, with 12 of these not contributing to theory development, leaving 23 studies for final data extraction. Please see Fig. 1 for a detailed PRISMA flow diagram.

### Description of the studies

The studies were conducted in multiple LMICs, predominantly in sub-Saharan Africa, including Kenya, South Africa, Tanzania, and Uganda. Additionally, studies were also carried out in India. While some research focused on national and regional levels (e.g. India and Uganda), others were concentrated at district or local levels (e.g. South Africa and Tanzania). The thematic focus includes understanding challenges and coordination in health systems (Kenya), system dynamics in mental health services (South Africa), public–private partnerships (Tanzania), multisectoral action for health (Uganda), and the convergence of human, animal, and environmental health sectors (India).

These studies employed qualitative and mixed-methods approaches, which were assessed for depth based on their capacity to elucidate generative causality. This was achieved through rigorous triangulation—cross-verifying mechanisms across data sources, stakeholders, and theoretical lenses—alongside iterative theory refinement, where hypotheses were tested and revised against empirical data. Reflexivity was applied to interrogate how power asymmetries shaped decision-making hierarchies and collaborative practices, while saturation ensured that emerging theories were robustly grounded in diverse participant perspectives and contextual realities. Additionally, studies that synthesized qualitative narratives with quantitative data, where applicable, provided deeper insights into the contingent pathways through which structural, institutional, and relational factors activated or constrained mechanisms This approach aligned with the realist objective of explaining what works, for whom, and under what conditions, ensuring methodological rigour and theoretical robustness.

### PTs and supporting empirical evidence

From the selected studies, a total of 73 unique CMOCs were identified. A full list of 73 CMOCs can be found in Supplementary File 1. These 73 CMOCs were organized into five broad ‘demi-regularities’ informing refinement of five IPTs into 5 PTs and the development of a new sixth PT. A list of demi-regularities and connected CMOCs can be found in Supplementary File 2. Table 1 outlines the five IPTs developed at the outset of the study. Additionally, Table 2 presents the refined PTs alongside the corresponding CMOCs and supporting studies.

**Table 1.** Initial programme theories

IPT 1: ownership	During the policy framing process, if the different sectors/departments are given equal ownership of the programme, then it increases the participation, coordination, and collaboration among stakeholders and supports balance in the power dynamics for planning and implementation of health interventions. This results in all sectors feeling valued, acknowledged, and empowered, which promotes engagement and collaborative intersectoral action.
IPT 2: goal alignment	If there is leadership support for interventions and sector goals are aligned with the sectoral priorities along with appropriate resources to support implementation, then this motivates, empowers, and engages staff, creating a sense of team efficacy and a shared sense of responsibility and accountability. This results in connectedness with the broader system and is more likely to result in better engagement/collaborative action in intersectoral collaborative interventions.
IPT 3: resource	If resources are not fairly distributed and are inaccessible to certain sectors, it can exacerbate dependency in weaker sectors and increase power imbalances. This leads to poorer engagement and diminished collaboration in programme implementation, ultimately hindering the success of intersectoral interventions.
IPT 4: trust and hierarchies	In settings where hierarchical structures exist, during ISC collaborative meetings or planning meeting, people may not speak up if other sectors’ views are not respected and/or information or data provided by one sector are not trusted by another sector. This will decrease the open communication, participation, coordination, and collaboration among the stakeholders of other sectors that lead to poor engagement/collaborative action in programmes.
IPT 5: role clarity	In settings where role clarity was not sufficiently delineated in policy or guidelines, there will be conflict and/or confusion among sectors and implementing teams, which can lead to poor engagement, work inefficiency, and poor accountability that will also impact the interpersonal relationships among sectoral stakeholders at all level, hindering implementation.

**Table 2.** Revised PTs with relevant CMOCs and supporting studies

Revised PTs	Revised PT	CMOC evidences	Source
PT 1: inclusive policy development	When health policy development ensures equal ownership and participation across various sectors and departments, it promotes inclusivity, mutual understanding, open communication, shared decision-making, equal resource allocation, shared vision, empowerment, and motivation. All sectors feel acknowledged and valued, leading to increased and sustainable engagement.	CMOC-7, CMOC-8, CMOC-9, CMOC-17, CMOC-20, CMOC-21, CMOC-22, CMOC-23, CMOC-25, CMOC-26, CMOC-43, CMOC-44, CMOC-45, CMOC-49, CMOC-50, CMOC-51, CMOC-66, CMOC-68, CMOC-69, CMOC-70, CMOC-71, CMOC-72, CMOC-73	Janse van Rensburg et al. 2018, Kamugumya and Olivier 2016, Kim et al. 2017, Pathak et al. 2022, Glandon et al. 2019, Buffardi et al. 2012, Chiari et al. 2023, Male et al. 2022
PT 2: leadership structures	When the leadership structures are democratic, then it creates an environment that values inclusivity, participation, shared decision-making, and transparent communication. This environment inspires commitment and empowers individuals by providing them with resources and autonomy, thereby reinforcing their collective belief in achieving shared goals. This fosters equitable partnerships, counters power imbalances, and strengthens connections, ultimately enhancing collaboration in intersectoral interventions.	CMOC-5, CMOC-6, CMOC-32, CMOC-34, CMOC-35, CMOC-36, CMOC-41, CMOC-46, CMOC-47, CMOC-53, CMOC-56, CMOC-57, CMOC-58	Nyawira et al. 2023, Ssennyonjo et al. 2021, Pathak et al. 2022, Etiaba et al. 2023, Balane et al. 2020
PT 3: equitable resource allocation	When resources are unevenly or inadequately distributed among sectors, it increases the dependency of less influential or less powerful sectors and exacerbates perceived power imbalances. This inequitable resource allocation not only hampers sectoral engagement and collaborative action in programme implementation but also undermines the sustainability of the partnership, as it fosters less enduring and more inequitable relationships among the collaborating sectors.	CMOC-9, CMOC-20, CMOC-22, CMOC-24, CMOC-26, CMOC-27, CMOC-40, CMOC-41, CMOC-42, CMOC-59, CMOC-60, CMOC-61, CMOC-64	Janse van Rensburg et al. 2018, Kamugumya and Olivier 2016b, Ssennyonjo et al. 2021, Bakhtiari et al. 2022, Kielmann et al. 2014
PT 4: communication and trust in hierarchical structures	When stakeholders in settings with hierarchical structures perceive that other sectors’ perspectives are not respected or lack trust in information provided by other sectors, then they withhold their input during intersectoral meetings or planning sessions. This lack of open communication impedes participation, coordination, and collaboration among stakeholders, ultimately resulting in poor engagement, poor information sharing, and limited collaborative action in programs.	CMOC-1, CMOC-3, CMOC-4, CMOC-10, CMOC-11, CMOC-12, CMOC-18, CMOC-19, CMOC-28, CMOC-30, CMOC-38, CMOC-39, CMOC-48, CMOC-52, CMOC-62, CMOC-63, CMOC-65, CMOC-67	Nyawira et al. 2023, Janse van Rensburg et al. 2018, Kamugumya and Olivier 2016a, Asaaga et al. 2021, Ssennyonjo et al. 2021, Pathak et al. 2022, Kielmann et al. 2014, Glandon et al. 2019
PT 5: role clarity and conflict resolution	When policy or guidelines lack clear role delineation, then conflicts and confusion among sectors and teams arise, leading to poor engagement, inefficiency, and reduced accountability, which in turn hinders programme implementation and impacts interpersonal relationships among stakeholders at all levels.	CMOC-2, CMOC-25, CMOC-31, CMOC-33, CMOC-34, CMOC-37, CMOC-49, CMOC-50, CMOC-51, CMOC-52	Nyawira et al. 2023, Kamugumya and Olivier 2016a, Asaaga et al. 2021, Ssennyonjo et al. 2021, Pathak et al. 2022
PT 6: interpersonal relationship	When stakeholders connect beyond their professional boundaries, ISC improves. These personal connections build trust and mutual respect, making stakeholders more willing to share resources and support each other. This helps balance power dynamics and leads to better teamwork and programme success.	CMOC-10, CMOC-12, CMOC-13, CMOC-16, CMOC-32, CMOC-50, CMOC-67	Janse van Rensburg et al. 2018, Ssennyonjo et al. 2021, Pathak et al. 2022, Glandon et al. 2019

#### Revised PT 1: inclusive policy development

This PT was supported by 23 CMOCs extracted from six documents (Table 2). This PT explains that granting equal ownership and meaningfully engaging all the sectors right from the stage of the policy development process enhance stakeholder participation, coordination, and collaboration, fostering balanced power dynamics and empowering all sectors. For instance, in a study conducted in Uganda (Ssennyonjo et al. 2022), a participant highlighted the importance of inclusive policy development by describing the formation of a multisectoral committee for the prevention and control of noncommunicable diseases:

First, we formed a multisectoral committee for the prevention and control of NCDs, and we met for some time. Of course, it involved all the key sectors, i.e. Gender, Agri-culture, Trade, Works, OPM, Finance, Presidents’ office. It was inaugurated in 2018. But the challenge is that it is inactive now because the other sectors don’t find the motivation. They don’t see it as their own mandate talking about health. (MOH-6, p. 1033)

This quote illustrates how the lack of sustained motivation and engagement from nonhealth sectors can lead to inactivity within such committees. The reason for this disengagement often stems from a sense of exclusion or irrelevance, where these sectors do not see the initiative as part of their mandate. This perception reinforces power imbalances, with the health sector taking the lead, while other sectors feel sidelined. In another example a study from Uganda (Male et al. 2022), where a respondent from a nonhealth sector pointed out that,

We are aware of the framework convention, but I think this convention is one of those which is (limited) to the health sector. (We) don’t use it—if it was ‘normal’ law that we use every day, I would (follow it) but the principal actor is the Ministry of Health. (Respondent from the nonhealth sector, p. 15).

This response suggests that policies primarily anchored within the health sector may not resonate with other stakeholders, limiting their engagement. While the exclusion of nonhealth sectors can reinforce power asymmetries, the issue is not solely one of stakeholder inclusion but also of stewardship. Stewardship, as conceptualized in public administration, entails the government’s responsibility to guide and embed diverse actors, including nonhealth sectors and NGOs, within public service missions. Rather than merely seeking broader participation, effective ISC requires an approach where governmental agencies actively cultivate shared responsibility and integrate different sectors into policy implementation processes. Without such stewardship, nonhealth actors may perceive policies as externally imposed rather than collectively owned, leading to disengagement and diminished collaboration. Furthermore, another study conducted in South Africa (Janse van Rensburg et al. 2018) highlights the interconnectedness between two sectors—state government and NGOs—adds another layer to this dynamic. As one participant noted:

Whether they get funded through grants, or through tax increases, or whatever, the work that NGOs do is the state’s responsibility. The only reason that they do it is because they do it on behalf of the state. So you can never financially untie yourself from an NGO…. (SW_TH, p. 1124)

This quote highlights how NGOs are often seen as extensions of the state, performing tasks that are fundamentally the state’s responsibility. However, if NGOs are not fully integrated into the policy development process as equal partners, power imbalances do arise, leading to inefficiencies and a lack of accountability. When NGOs feel that their work is merely an extension of state responsibilities without having a say in policy decisions, this can further exacerbate power disparities and hinder effective collaboration.

These illustrative examples from the literature underscore the critical role of inclusive policy development in fostering balanced power dynamics and effective ISC. When all sectors are equally involved and their contributions are valued, it leads to a more collaborative environment where shared goals can be pursued more effectively (Travis et al. 2002, Haines et al. 2004, Sheikh et al. 2011). Conversely, the exclusion of certain sectors not only diminishes their engagement but also undermines the overall success of intersectoral initiatives, as imbalanced power dynamics hinder cooperation and coordination across sectors (Harris et al. 2012, Shankardass et al. 2012).

The refinement of the IPT into the revised PT was informed by several key findings. One of the most striking insights was the recognition that merely granting equal ownership to different sectors during the policy framing process was insufficient to guarantee sustained participation and collaboration. The empirical evidence, particularly from Uganda and South Africa, highlighted that even when multisectoral committees were established with broad representation, their effectiveness was often undermined by a lack of sustained motivation among nonhealth sectors. The disengagement of these sectors was not solely due to their formal exclusion but also stemmed from the perception that health-focused policies were not within their mandate, thereby reinforcing power imbalances rather than addressing them. This finding necessitated a shift in the PT to explicitly acknowledge that transparent communication is a crucial element in ensuring active and continued participation, beyond formal inclusion.

Another significant insight came from the study in South Africa, which revealed that NGOs, despite playing a critical role in service delivery, often perceived themselves as mere extensions of the state rather than as equal partners in policy development. This dependency on state funding and directives created an imbalance in decision-making power, where NGOs lacked meaningful influence over policy direction. This finding underscored the need to revise the IPT by explicitly addressing the dominance of any single sector, rather than assuming that equal ownership alone would balance power dynamics. The revised PT thus reflects a more nuanced understanding that equitable participation requires not only shared ownership but also mechanisms that prevent any one sector—whether governmental or nongovernmental—from exerting disproportionate control over policy decisions.

Furthermore, evidence from Uganda demonstrated that while intersectoral engagement was initiated, nonhealth sectors often viewed ISC committees as peripheral to their core responsibilities. This reluctance was linked to a lack of perceived value and relevance in engaging with health policies, reinforcing the need to incorporate transparent communication as a key mechanism in the revised PT. If sectors are not only granted ownership but also meaningfully engaged through clear, contextually relevant communication, they are more likely to see their contributions as integral to the policy process. This shift in emphasis ensures that the revised PT captures not just the structural aspects of ISC but also the relational and perceptual factors that influence sustained engagement.

These findings collectively led to the refinement of the IPT by integrating transparent communication as a mechanism for fostering genuine collaboration and by reframing the concept of power balance to explicitly recognize and mitigate the risk of sectoral dominance in decision-making. The revised PT, therefore, offers a more comprehensive and context-sensitive explanation of how inclusive policy development can support effective ISC in health interventions. An illustrative example of how these findings contributed to the refinement of the theory is provided in Table 3.

**Table 3.** Illustrative example of theory refinement

IPT	Theory refinement illustration
IPT 1	During the policy framing process, if the different sectors/departments are given equal ownership of the programme, then it increases the participation, coordination, and collaboration among stakeholders and supports balance in the power dynamics for planning and implementation of health interventions. This results in all sectors feeling valued, acknowledged, and empowered, which promotes engagement and collaborative intersectoral action.
Revised PT	When health policy development ensures equal participation from the various sectors, it promotes inclusivity, mutual understanding, open communication, shared decision-making, shared vision, empowerment, and motivation. Participants feel that their perspectives and contributions are valued, which enhances their sense of belonging and creates a cohesive environment where stakeholders are more willing to cooperate and support one another. This acknowledgment helps to balance power dynamics and supports sustainable engagement by recognizing the indispensable role of state responsibility in healthcare.
Practical evidence from a study conducted in South Africa	It was made clear, though, that the state holds primary responsibility for mental healthcare. ‘Whether they get funded through grants, or through tax increases, or whatever, the work that NGOs do is the state’s responsibility. The only reason that they do it is because they do it on behalf of the state. So you can never financially untie yourself from an NGO… (SW_TH).’ The participant’s perspective suggests that regardless of the funding source, whether it be grants or tax increments, the activities carried out by NGOs ultimately remain the responsibility of the state. The participant emphasizes that NGOs operate on behalf of the state and therefore cannot completely detach themselves from financial reliance on the state. In the study, it was further reported that social workers from the NGO were valuable role‐players in a collaborative arrangement between the state psychiatric hospital and a specialized mental health NGO. Social workers at the hospital served as gatekeepers for the NGO to specialized services, while social workers from the NGO conducted home visits and provided other community‐based services for the hospital whenever they got sufficient acknowledgments for their service.
What changed in the revised PT?	Revised PT broadens the scope of the original concepts by explicitly emphasizing inclusivity, mutual understanding, open communication, and shared decision-making. These elements are crucial in contributing to a shared vision, empowerment, and motivation among participants. The revised PT deepens the understanding of value and belonging by stressing that participants feel their perspectives and contributions are recognized and valued, thereby enhancing their sense of belonging and fostering a cohesive environment. This nuance suggests that when participants feel valued, it leads to a greater willingness to cooperate and support each other. Additionally, the revised PT elaborates on the importance of balancing power dynamics by explaining that the acknowledgement of all sectors is essential for creating equitable power relations and supporting sustainable engagement. It also introduces the concept of recognizing the indispensable role of authorities within collaborative policies, ensuring that all voices are heard and respected in the planning and implementation processes. Practical evidence connection The study highlights the state’s primary responsibility for mental healthcare, irrespective of the funding source (grants or tax increases). NGOs act on behalf of the state, indicating a reliance on state acknowledgment and financial support. Social workers from NGOs and the state psychiatric hospital collaboratively contribute to mental health services, with their roles and acknowledgments being crucial for effective collaboration. How the revised PT reflects the example Value and acknowledgment: The example illustrates the importance of NGOs receiving sufficient acknowledgment for their services, which supports the revised PT’s emphasis on participants feeling valued and their contributions being recognized. Collaboration and power dynamics: The collaborative arrangement between social workers from the state hospital and NGOs in the example supports the revised PT’s focus on shared decision-making and mutual support, which balance power dynamics and enhance cooperation.

#### PT 2: leadership structures

A total of 13 CMOCs extracted from eight documents, as detailed in Table 2, informed the refinement of PT2. This PT describes the importance of leadership support within ISC to balance the impact of power. It highlights that when leaders adopt democratic and inclusive approaches, it creates an environment where inclusivity, participation, shared decision-making, and transparent communication are highly valued. This environment encourages individuals to be committed and motivated by giving them the necessary resources and autonomy. Consequently, this strengthens their collective belief in achieving shared goals. Such an environment promotes fair partnerships and fosters strong personal connections among different sectors, thereby reducing power imbalances and improving collaboration in intersectoral interventions. For example, in a study conducted in Afghanistan (Pathak et al. 2022), one participant described the important aspects of a successful partnership that demonstrated this inclusive leadership approach:

Timely updates, communications, a consultative decision-making approach were some of the key features of this partnership. (Kabul Polytechnic University, p. 9)

This quote demonstrates how democratic leadership—characterized by timely updates, open communication, and a consultative approach—can facilitate successful partnerships. When all sectors remain well informed and actively engaged in decision-making, it fosters trust and ensures alignment towards common goals. This inclusive approach nurtures collective responsibility and commitment, which are essential for effective ISC (Gilson 2003, Bryson et al. 2006). However, the feasibility of participative leadership must be understood within its contextual constraints. In settings with limited statehood, such as Afghanistan, governance challenges and political instability may hinder the implementation of participative decision-making. In such contexts, while inclusivity remains a critical goal, leadership structures must also account for administrative clarity and functional governance mechanisms to maintain order and efficiency.

Conversely, excessive centralization of power can stifle participation and hinder collaboration. A study conducted in Uganda (Ssennyonjo et al. 2022) illustrates the negative consequences of nondemocratic leadership within ISC:

The power in the Cabinet is overcentralised. The President is too powerful. That is why we do not have standing cabinet committees. We have a lot of ad-hoc committees, which is not good. When you have standing committees, they allow you to disperse power to constituencies that will take an interest in thematic issues. (NSA-1, p. 1029)

This quote highlights how the concentration of power within a few decision-makers can weaken collaborative structures. The reliance on ad-hoc committees, rather than standing committees with sustained engagement, limits opportunities for meaningful participation and continuity. Without mechanisms that disperse power effectively, sectors outside the dominant authority may struggle to assert their influence, leading to imbalances that undermine intersectoral cooperation (Franco et al. 2004, Bryson et al. 2006, Kickbusch and Gleicher 2012).

It is also important to acknowledge that participative decision-making, while beneficial in many respects, is not without challenges. Overemphasis on consensus-building can slow down decision-making processes, particularly in complex governance environments where multiple, often competing, perspectives must be reconciled. Effective leadership structures must therefore balance democratic participation with administrative clarity, allowing for structured yet adaptive governance that can respond to evolving challenges. Leadership that spans hierarchical boundaries while maintaining decision-making efficiency is crucial to sustaining ISC without unnecessary delays or bureaucratic stagnation.

These examples underscore the significance of leadership structures in shaping power relations within ISC. While participative leadership can counteract power imbalances and foster more inclusive collaboration, its practical implementation requires careful consideration of governance capacity and context-specific constraints. The refined PT thus highlights the necessity of leadership approaches that not only promote inclusivity and shared decision-making but also ensure administrative efficiency and adaptability, striking a balance between democratic engagement and structured governance to achieve successful intersectoral interventions.

#### PT 3: fair resource allocation

The third PT was supported by 13 CMOCs, as outlined in Table 2. It explains that when resources including funding and skilled personnel are not distributed fairly among sectors, it makes weaker sectors more dependent and worsens power imbalances. This leads to poorer collaboration and weakens the partnership, making it less sustainable. A study conducted in India (Yasobant et al. 2021) exemplifies the impact of bureaucratic inefficiencies on resource accessibility. One participant described their frustration with the complex administrative processes that hinder timely access to government funding:

Ah! Government funding to me remains a mystery (laughing). We are still struggling to access some government funding for a project since last year (2018). We always hear there is money, but we don’t see it. Perhaps it is the way the system is structured and bureaucracy issues…. (Interview 12, Animal Health, p. 13)

This quote highlights how procedural delays and administrative inefficiencies, rather than explicit resource denial, can obstruct fair resource allocation. While funding may exist, systemic challenges such as bureaucratic complexity and unclear mechanisms of disbursement create barriers to access, disproportionately affecting sectors with weaker institutional influence. In contrast, sectors that possess stronger institutional positioning—such as finance ministries or dominant health agencies—may have greater capacity to navigate these bureaucratic processes, thereby consolidating their control over resource distribution. Similarly, disparities in skilled personnel further reinforce these imbalances. A study conducted in South Africa (Janse van Rensburg et al. 2018) illustrates how financial constraints directly impact workforce capacity:

‘Skilled workers equals money, and money is our only drawback. (CC_NGO4, p. 1125)

This statement underscores the interconnection between financial resources and human capital. Sectors with inadequate funding struggle to recruit and retain skilled professionals, limiting their ability to engage meaningfully in collaborative efforts. Conversely, well-resourced institutions—such as national ministries, international donors, or dominant government agencies—are better positioned to attract skilled personnel, thereby consolidating their decision-making authority and influence. Further evidence from Tanzania (Kamugumya and Olivier 2016) reveals the structural and relational dimensions of resource allocation at the district level. Although local governments are formally responsible for fund allocation and provider selection, power is distributed unevenly. The Community Health Management Team (CHMT), possessing strong biomedical expertise and financial resources, lacks an in-depth understanding of public–private partnerships (PPPs). Meanwhile, the District Council Team wields significant political influence over both the CHMT and the Community Health Service Board (Kamugumya and Olivier 2016), often prioritizing politically driven interests in resource allocation. This imbalance shapes engagement with nonstate actors and further complicates the equitable distribution of funds. Moreover, competing priorities within decentralized governance structures create additional challenges in optimizing resource allocation to improve system performance through PPPs.

These examples highlight that resource imbalances are not solely financial but also embedded within governance structures and institutional relationships. Sectors with stronger institutional positioning—whether government agencies with greater fiscal control, influential ministries such as finance, or international donors—often dictate the terms of resource distribution. Meanwhile, weaker sectors, including underfunded departments or community-level organizations, struggle to assert influence, perpetuating dependency and reinforcing top-down dynamics. The discussion of resource disparities can be effectively framed through the hardware and software dimensions of health systems. Structural challenges, such as bureaucratic inefficiencies and funding constraints, constitute the hardware components—formal mechanisms determining control over resources. These structural mechanisms generate conditions that reinforce hierarchical authority, limit autonomy, and constrain equitable resource allocation across sectors. Concurrently, relational challenges—such as power asymmetries, institutional influence, and sectoral dependency—represent the software dimension, shaping mechanisms related to trust-building, interaction patterns, and decision-making dynamics within ISC. These relational mechanisms trigger responses from stakeholders that either facilitate or impede meaningful engagement, depending on how power relations and dependencies manifest.

The interplay between hardware and software factors thus produces generative causation, influencing whether collaboration remains equitable or whether power disparities persist and hinder meaningful stakeholder participation. The revised PT explicitly recognizes these complexities by positing that equitable participation in ISC emerges through mechanisms activated by fair resource distribution combined with transparent governance arrangements. When governance mechanisms are transparent and inclusive, they mitigate power disparities, creating conditions for more balanced stakeholder interactions and equitable resource sharing. Conversely, the absence or weakness of such mechanisms activates negative generative processes, reinforcing existing power asymmetries and obstructing meaningful collaboration.

Therefore, empirical evidence presented in this study highlights that equitable participation in ISC is contingent upon activating positive generative mechanisms that not only ensure fair resource distribution but also institutionalize transparent governance arrangements capable of moderating power disparities.

#### PT 4: hierarchies, communication, and trust

This PT was supported by 18 CMOCs extracted from seven studies. This PT highlights the impact of hierarchical structures on communication and trust within ISC. The formal hierarchies typically emerge from established structures, such as government agencies, where authority is clearly defined by laws, policies, or organizational roles (Wildridge et al. 2004). These hierarchies dictate who has the final say in decisions, often sidelining sectors with less formal power, such as NGOs or community groups, whose voices may not be as influential. Informal hierarchies, on the other hand, are shaped by factors like experience, expertise, or personal relationships, which can also lead to power imbalances (Mcdonald et al. 2012). In such situations, power is often exerted through control over resources, information, communication, or the ability to influence key decisions. This can result in some sectors feeling undervalued or unheard, which weakens the collaboration as a whole (Bennett and Gadlin 2012). This was demonstrated in a study conducted in Uganda (Ssennyonjo et al. 2022) where a participant highlighted the impact of hierarchical imbalances, stating,

At times, we are called to meetings where decisions have already been made by the central authorities. Our role is often to simply endorse these decisions without any substantial contribution. (Respondent 3, national level, p. 7)

When stakeholders feel that their input is merely a formality rather than a valued contribution, they are likely to withhold further input. This dynamic stifles open communication and trust, both of which are essential for effective collaboration across sectors. The lack of clear communication channels fosters a sense of alienation, leading to feelings of being ‘out of the loop’, which in turn discourages active participation (Hargie et al. 2004, WHO 2008, Edmondson and Lei 2014). This issue was similarly highlighted in another study conducted to improve PPPs in Tanzania (Kamugumya and Olivier 2016) where a participant stated,

I have heard of the CHSB, but I don’t know what is usually discussed. We don’t have a representative. We (private providers) are considering forming an association and then appoint leaders, who would then be our representatives. But we have not yet managed to form that association. (Participant 21, Bagamoyo 2014, p. 4)

This quote illustrates how the absence of representation can lead to a disconnection between sectors. Without a seat at the table, private providers or other stakeholders may feel that their voices are not heard. This issue was again evident in another study conducted at the subnational level in Africa (Mwisongo et al. 2016), where an official from Guinea highlighted the issue of voices not being heard in hierarchical settings:

We at the sub-national levels have little say … We are often told to do several things since these dialogues began and sometimes they are contradictory… Of course, you cannot question, you follow orders. (Subnational level official, Guinea, p. 344)

This quote reflects the power imbalances inherent in hierarchical structures, where those at lower levels are expected to follow orders without question. When stakeholders are excluded from decision-making processes or feel that their contributions are not valued, it leads to disengagement, poor information sharing, and ultimately, ineffective collaboration. These examples illustrate how poor communication and power imbalances in hierarchical structures can undermine ISC. The practical examples provided have refined the IPT by highlighting how hierarchical structures, when perceived as disrespectful or untrustworthy, lead to stakeholders withholding input during intersectoral meetings. This disengagement disrupts open communication, which is crucial for effective collaboration. The revised PT now emphasizes that a lack of trust and respect in these settings impedes participation, coordination, and information sharing, ultimately undermining collaborative action in programmes. This adjustment offers a more nuanced understanding of the specific impacts of hierarchy on ISC.

#### PT 5: role clarity and conflict resolution

This PT was supported by 10 CMOCs extracted from 11 studies. The PT explains when roles and responsibilities are not clearly defined, ambiguity in decision-making can arise, allowing certain stakeholders to command more influence over others. This situation also strains interpersonal relationships among stakeholders, hindering the effective collaboration and communication necessary to achieve common goals. Unclear roles in policies and guidelines not only create confusion and inefficiencies but also have significant implications for power dynamics, such as marginalizing less-resourced sectors, ultimately leading to poor collaboration outcomes. For example, a study conducted in Odisha, India (Kim et al. 2017), illustrates this issue, where a participant from the social welfare department expressed frustration over the lack of role clarity:

There is a lot of confusion over who is responsible for what. Sometimes health workers end up doing tasks that Integrated Child Development Scheme workers should be doing and vice versa. This creates tension and affects our ability to work together effectively. (Respondent from the ICDS department, p. 16)

This statement highlights the blurring of sectoral responsibilities, which not only disrupts collaboration but also leads to hierarchical dominance, where one sector (in this case, health) assumes greater authority, reducing the influence of others such as the Integrated Child Development Scheme (ICDS). In ISC, the absence of clearly assigned mandates enables more influential sectors to take over decision-making, reinforcing power asymmetries and reducing the agency of less-resourced sectors (French and Raven 2004). A similar challenge emerged in Uganda (Male et al. 2022), where the implementation of Article 5.3 of the WHO Framework Convention on Tobacco Control faced resistance from nonhealth sectors due to a perception that tobacco control was exclusively the Ministry of Health’s responsibility:

My work is not public health. This law is about tobacco control—our colleagues at the Ministry of Health understand this law differently. This law is alien to our daily (working) lives. (Respondent from nonhealth sector, p. 15)

This statement reflects how the lack of shared ownership across sectors can alienate nonhealth actors, discouraging their participation in ISC initiatives. When policies are framed as the responsibility of a single sector rather than a cross-sectoral priority, other stakeholders may disengage, perceiving their role as secondary or irrelevant. This weakens the whole-of-government approach required for effective intersectoral action and reinforces sectoral silos in policy-making and implementation. Moreover, the lack of institutionalized processes to define and coordinate intersectoral roles exacerbates these challenges. When no clear structures exist to facilitate cross-sector engagement, dominant sectors dictate the terms of collaboration, leading to power imbalances. The study by Nyawira et al. (2023) in Kenya further demonstrates how role ambiguity contributes to these imbalances:

There are no clear-cut definitions of what you’re supposed to do. You just do the roles as they come. One day you’re a procurement officer, another day you’re an accountant then you are engaging at an international level. Also, there was no handing over. I came from a health facility to this office and therefore needed some sort of orientation as regards the job description. But when I landed there, I was told, this is your office. And I wondered, so what exactly am I supposed to do? I’m still learning. (Respondent 4, national level, p. 8)

While this example is drawn from an individual experience, it illustrates a broader systemic issue—where institutions lack clear role definitions in intersectoral governance. In ISC, such ambiguities often result in power disparities, where the most politically or financially dominant sectors take the lead, leaving others without a clear mandate or authority in decision-making. This structural disorganization not only creates inefficiencies but also erodes trust, as marginalized stakeholders may feel excluded from substantive participation.

These findings underscore that role ambiguity in ISC is not merely an administrative issue but a structural challenge that reinforces power asymmetries. When roles are poorly defined across sectors, dominant actors consolidate influence, marginalized sectors lose decision-making power, and collaboration becomes less inclusive and effective. The revised PT reflects these complexities by highlighting that unclear intersectoral roles do not just lead to confusion or inefficiencies but also exacerbate power struggles, limit accountability, and weaken collaborative governance. Addressing these challenges requires institutional mechanisms that clearly delineate sectoral responsibilities, ensure equitable participation, and prevent the monopolization of decision-making authority by more powerful actors (Mintzberg 1993, Mcdermott et al. 2015).

#### PT 6: interpersonal relationships

This PT emerged from the extensive synthesis of the evidence which was not conceptualized at the initial stage of IPT development. This theory was supported by seven CMOCs and five studies. The PT describes that, when people from different sectors work together closely and get along well, it builds trust and makes communication easier. This positive environment also means they respect each other more. As a result, there are fewer arguments, and everyone feels more equal in decision-making. This teamwork leads to improved cooperation, greater levels of involvement, and more efficient and effective working. For example, a study from South Africa (Janse van Rensburg et al. 2018) that evaluated the collaboration between a state psychiatric hospital and a specialized mental health NGO reported that the collaboration was greatly strengthened by the trusting relationships between social workers from both institutions. One participant highlighted this, stating,

The trust and respect we built with the hospital’s social workers made it easier to coordinate services. We knew we could rely on each other to deliver what was needed for the patients. (Respondent from the NGO, p. 12)

This trust facilitated smooth coordination and service delivery, ultimately leading to better patient outcomes. Similarly, in India (Yasobant et al. 2021), the importance of interpersonal connections was emphasized in the operationalization of the One Health approach. Building trust among stakeholders from various sectors, including human and animal health, was identified as a critical enabler for successful ISC. One respondent reflected,

The personal connections we’ve made with our counterparts in the health department have made a big difference. We now share information more freely, and this has improved our ability to respond quickly to the needs of the community. (ICDS worker, Odisha, p. 16)

These interpersonal connections helped bridge the gaps that formal power structures and unclear roles often create, enabling more effective collaboration. These examples demonstrate that when interpersonal relationships are strong, they can neutralize the impact of power imbalances that often hinder ISC. In Tanzania (Kamugumya and Olivier 2016), the success of PPPs was similarly attributed to the good relationships built over time between stakeholders, which facilitated better coordination and goal achievement. A district health official noted,

The success of our partnership with the private sector can largely be attributed to the good relationships we’ve built over time. Without this trust, it would be much harder to coordinate our activities and achieve our goals. (District health official, Tanzania, p. 5)

When stakeholders trust each other and communicate openly, they are more likely to overcome the challenges posed by hierarchical dominance and misaligned priorities. This interpersonal trust creates a more egalitarian environment, where all parties feel valued and empowered to contribute, thereby improving the overall efficiency and effectiveness of ISC. These illustrative examples demonstrate the importance of interpersonal connection within formal structures. Based on the evidence and practical illustrations discussed earlier, the IPTs have been refined to enhance their comprehensiveness for further testing through a realist evaluation approach. These refinements ensure that the PTs are more robust, contextually relevant, and actionable. They provide clearer guidance for policymakers and practitioners aiming to optimize ISC in health systems, making them better equipped to address the complexities and dynamics of ISC effectively.

## Discussion

This realist synthesis advances the understanding of how power dynamics shape ISC in health programmes across LMICs. By synthesizing evidence from 23 studies, six PTs were identified, each elucidating distinct yet interconnected mechanisms through which power influences ISC outcomes. The findings reveal that power is neither a static nor a monolithic force within ISC but rather a dynamic, relational, and context-dependent phenomenon. The study extends existing scholarship by offering a more nuanced understanding of how power operates through governance structures, leadership practices, resource distribution mechanisms, and interpersonal relationships. This synthesis also builds on and refines theoretical perspectives, drawing from complexity leadership theory (Uhl-Bien et al. 2007), stakeholder theory (Freeman 2010), collaborative governance theory (Ansell and Gash 2008), resource dependence theory (Pfeffer and Salancik 2015), and social exchange theory (Blau 2017) to deepen our understanding of power in ISC.

The findings underscore the critical role of inclusive policy development in fostering effective ISC. Collaborative governance theory (Ansell and Gash 2008) suggests that participatory decision-making enhances policy legitimacy and coordination. However, this review highlights that while inclusivity can strengthen engagement, it is not without challenges. Differences in sectoral mandates, conflicting priorities, and asymmetries in decision-making power can slow policy processes and generate tensions (Ataguba et al. 2011). This necessitates mechanisms to balance inclusivity with efficiency, such as structured stakeholder engagement frameworks and clearly defined governance mechanisms Moreover, governance structures shape the extent to which stakeholders can meaningfully participate.

In settings with weak institutional frameworks, engagement may be superficial, with dominant sectors exerting disproportionate influence (Habib and Taylor 1999, Swilling and Russell 2002). By contrast, in well-institutionalized governance systems, participatory policy-making enhances long-term collaboration (Herdiana et al. 2018). A study on Brazil’s reform highlighted that inclusive policy-making improved coordination and stakeholder buy-in (Atun et al. 2015); similar observations were documented in the following studies (Burgess et al. 2016, Herdiana et al. 2018, Holcomb et al. 2022).

Comprehensive consultations with a broad range of stakeholders, including civil society, healthcare professionals, and government agencies, enhanced the legitimacy of policies and ensured diverse needs and perspectives were considered, leading to more sustainable and effective health outcomes. While these cases illustrate the advantages of inclusive policy-making, its impact varies by context. In some settings, sectoral coordination is facilitated through strong institutional frameworks, whereas in others, governance structures and power imbalances limit effective engagement. Contrasting findings in some contexts highlight the complexities involved. For example, a study in Ghana found that while inclusive policy processes can enhance stakeholder engagement, they can also lead to delays and conflicts due to differing priorities and interests among sectors (Ataguba et al. 2011).

Additionally, the role of NGOs in policy development differs across governance systems. In some settings, such as South Africa, NGOs are deeply integrated into state functions, often acting as extensions of government responsibilities (Habib and Taylor 1999, Swilling and Russell 2002). However, in other contexts, such as certain Latin American or Southeast Asian countries, NGOs function independently or even in opposition to state policies, leading to different power dynamics in ISC (Hewison 1997, Clarke 2006, Weiss 2006, Dagnino 2008, Alvarez et al. 2018). Recognizing these variations ensures a more context-sensitive understanding of ISC and the mechanisms that enable or hinder inclusive policy development. These contextual insights informed the refinement of PT1 by highlighting that while inclusive policy development fosters engagement, its effectiveness depends on how power imbalances, sectoral mandates, and governance structures are navigated. Recognizing these nuances ensures that policies are not only inclusive but also actionable within specific political and institutional settings.

The findings on leadership reaffirm that leadership is a crucial determinant of ISC effectiveness, but they also highlight the limitations of a binary conceptualization of leadership as either hierarchical or participatory. Complexity leadership theory (Uhl-bien et al. 2007) offers a more nuanced perspective, suggesting that effective ISC governance requires an interplay between administrative leadership (to provide stability and regulatory clarity), adaptive leadership (to enable frontline innovation), and enabling leadership (to bridge hierarchical and networked structures).

This finding resonates with a study that explored leadership dynamics in emergency contexts that found democratic leadership, characterized by inclusivity and trust, leads to higher levels of follower motivation and collaboration (Rosing et al. 2022). Similarly, another study conducted on school-level leadership demonstrated that democratic leadership among school principals not only promotes distributed leadership but also ensures that leadership responsibilities are shared, leading to a more inclusive and collaborative environment (Kilicoglu 2018).

Research exploring leadership and sense-making for primary healthcare in South Africa highlights the role of systems thinking and collective problem-solving in fostering an inclusive, collaborative environment, key aspects of democratic leadership (Gilson et al. 2014a). Additionally, a qualitative assessment of community-directed interventions in rural Malawi and an analysis of leadership challenges in scaling up antiretroviral therapy in sub-Saharan Africa further emphasize the importance of participative leadership in complex health initiatives, reinforcing the need for transparent communication and inclusive decision-making to achieve organizational success (Van Damme et al. 2006).

The congruence between these studies suggests a broader applicability of democratic leadership principles across different organizational settings, further reinforcing the critical role of participative decision-making and transparent communication in achieving programme success. This study finds that while participative leadership fosters engagement and shared ownership, overly decentralized decision-making can create inefficiencies (Gilson and Agyepong 2018, Gilson et al. 2023). In LMICs, where governance structures often lack coherence, leadership approaches must be adapted to local realities. For instance, leadership in highly centralized systems must integrate participatory elements to encourage collaboration, while decentralized contexts require mechanisms to ensure alignment across sectors (Belrhiti et al. 2024).

Power imbalances in ISC are often reinforced by disparities in resource allocation. Resource dependence theory (Pfeffer and Salancik 2015) suggests that organizations with greater resource control exert disproportionate influence, shaping policy agendas and decision-making authority. This study corroborates existing evidence that sectors with greater financial and technical resources tend to dominate ISC processes, often marginalizing social welfare and education sectors (Buse and Walt 1997, Hafner and Shiffman 2013). However, this synthesis also highlights that inequitable resource distribution is not merely a function of power dynamics but is also shaped by structural constraints, such as donor-driven funding priorities and weak fiscal decentralization (Msuya 2004, Shiffman 2006).

While transparent budgeting and participatory funding mechanisms may mitigate inequities, they are insufficient in contexts with weak governance capacity. Thus, state stewardship is essential to ensuring that resource allocation is aligned with broader public health objectives rather than sector-specific interests (Kamugumya and Olivier 2016). The study findings resonate with a study in Brazil that found while resource allocation disparities existed, effective leadership and strong governance structures mitigated the negative impacts (Victora et al. 2011).

The study suggested that transparent and participatory governance mechanisms could help balance power dynamics, ensuring all sectors have a voice in decision-making processes, even when resource disparities are present. The issue of resource disparity is deeply intertwined with structural hierarchies and sectoral funding mechanisms. In many contexts, the health sector tends to receive more funding than social welfare, education, and other allied sectors, primarily due to donor-driven vertical programmes that prioritize disease-specific interventions, such as malaria, tuberculosis, and human immunodeficiency virus (HIV)/acquired immunodeficiency syndrome (AIDS) (Msuya 2004, Shiffman 2006). These programmes often channel resources directly to health agencies, leaving other sectors underfunded and dependent on health-led initiatives, thereby reinforcing a power imbalance where the health sector assumes a dominant role in decision-making (Buse and Walt 1997, Hafner and Shiffman 2013).

Additionally, resource imbalances are not only sectoral but also hierarchical, as national-level agencies typically control funding flows, leaving district and community-level organizations under-resourced (Sridhar and Woods 2013). This dynamic was evident in Tanzania, where the CHMT, despite having strong biomedical expertise and access to health-specific funding, lacked the institutional authority to allocate resources equitably across sectors. Meanwhile, the District Council Team, which wielded greater administrative power, prioritized politically driven interests over technical health considerations, reinforcing power asymmetries (Kamugumya and Olivier 2016). This example shows the strong interconnectedness of PT on leadership and resource allocation, which is vital for a more sustainable ISC. Strong, inclusive leadership ensures that resources are distributed fairly, which, in turn, empowers all sectors to participate fully and equally. This creates a balanced collaboration, reducing power imbalances and fostering sustained engagement (Gilson 2003, Siddiqi et al. 2009, Frenk and Moon 2013).

Conversely, when leadership fails to address resource disparities, it can weaken the motivation of less-resourced sectors, leading to a less effective partnership. Thus, leadership directly influences how resources are shared and how power dynamics are managed within ISC, making the two theories closely interlinked. Resource dependence theory and equity theory further elucidate the dynamics of resource allocation in ISC. Resource dependence theory posits that organizations are dependent on external resources, shaping power relations and organizational behaviour (Pfeffer and Salancik 2015). In the context of ISC, sectors with greater resources hold more power, influencing decisions and actions within the partnership. Perceived inequities in resource allocation can lead to dissatisfaction and reduced cooperation among stakeholders, highlighting the importance of equitable resource distribution for sustaining partnerships.

ISC often falters due to role ambiguity and fragmented accountability structures. This study finds that clearly defined roles enhance operational efficiency and reduce power imbalances, particularly in complex governance environments (Gilson et al. 2014). Role theory (Biddle 1986) and network governance theory (Agranoff 2007) provide useful frameworks for understanding these dynamics. Unlike traditional bureaucratic structures, ISC relies on distributed governance, where responsibilities are shared across multiple actors. However, without formal mechanisms for role clarification, power vacuums emerge, enabling dominant sectors to control decision-making.

Moreover, this study highlights that role clarity must extend beyond individual organizations to encompass intersectoral dynamics. Conflict management strategies that work within organizations do not necessarily translate to ISC, where power asymmetries, competing mandates, and governance fragmentation pose distinct challenges (Scott and Gerardi 2011). Studies have shown that establishing trust and collaborative governance structures enhances health security outcomes (Emerson 2018, Grootjans et al. 2022, Robert et al. 2022).

Trust is a fundamental enabler of ISC, mitigating hierarchical constraints and fostering cooperative decision-making. Social capital theory (Lin et al. 2001) and relational coordination theory (Gittell 2006) suggest that strong interpersonal networks enhance coordination by fostering shared goals and mutual respect. This study reaffirms that informal relationships can compensate for institutional weaknesses, enabling stakeholders to navigate power imbalances and enhance collaborative efficacy (Kuruvilla et al. 2018). However, the findings also caution against over-reliance on informal mechanisms, as they may entrench exclusivity and limit broader stakeholder engagement.

Clearly defined roles ensure that all stakeholders understand their responsibilities, which not only improves operational efficiency but also neutralizes the negative impact of power dynamics (Indicators 2010, Gilson et al. 2014, Buse et al. 2023). When roles are unclear, it can create power vacuums where more dominant stakeholders take control, marginalizing others and leading to conflicts and inefficiencies. Conversely, when roles are clearly delineated, it empowers all stakeholders by ensuring that each person or group has a specific, recognized function within the collaboration. This balance of power reduces the likelihood of dominance by any one group, fostering a more equitable and cooperative environment.

Research consistently shows that unclear roles, overlapping responsibilities, and weak institutional frameworks exacerbate power imbalances within health systems, undermining collaboration and efficiency (Emerson 2018, Ly et al. 2018, Conway et al. 2019, Al-Worafi 2024). A narrative synthesis of health system decentralization in the Indo-Pacific highlighted that decentralized governance can enhance performance in post-conflict settings by clearly defining the roles and responsibilities of various stakeholders (Brennan and Abimbola 2023). Similarly, a study on conflict management within healthcare teams emphasized the importance of role clarity in reducing interpersonal conflicts, thus balancing power. Well-defined roles and responsibilities helped mitigate conflicts and fostered a more collaborative working environment (Scott and Gerardi 2011).

These studies illustrate that clear role definitions are crucial in improving coordination, reducing conflicts, balancing the impact of power, and enhancing overall performance in healthcare settings. Role theory and conflict resolution theory provide a deeper understanding of these dynamics. Role theory suggests that clarity in roles reduces ambiguity, enhancing individual performance and organizational efficiency (Biddle 1986) Conflict resolution theory highlights the importance of effective communication and conflict management strategies in resolving interpersonal and intergroup conflicts, which are often exacerbated by unclear roles and responsibilities (Fisher et al. 2011).

Interpersonal relationships (PT 6) are critical for fostering trust and communication, which are essential for mitigating the risk of power disparities leading to exclusion or disengagement. This finding was corroborated by multiple studies highlighting the significance of interpersonal relationships and continuous communication in enhancing the effectiveness of multisectoral nutrition initiatives (Enn 2017, Alderwick et al. 2021, Banerjee 2021, Aivalli et al. 2024). Social capital theory (Lin et al. 2001) and relational coordination theory (Gittell 2006) both offer valuable frameworks for understanding how strong interpersonal relationships and effective coordination can help neutralize the impact of power dynamics in ISC.

Social capital theory posits that social networks, along with the norms of reciprocity and trustworthiness that develop within them, facilitate coordinated actions among individuals and groups. In the context of ISC, this theory suggests that when stakeholders build strong social networks characterized by trust and mutual support, they are better equipped to collaborate effectively, regardless of the formal power structures in place. By fostering a culture of reciprocity and trust, social capital theory helps to mitigate power imbalances, ensuring that all sectors feel equally valued and empowered to contribute to the collaboration. This can be particularly effective in preventing more dominant sectors from overshadowing others, as the shared social capital acts as a balancing force.

Relational coordination theory further supports this by emphasizing the importance of relationships characterized by shared goals, shared knowledge, and mutual respect (Gittell et al. 2010). According to this theory, such relationships improve organizational performance, especially in complex and interdependent environments like those often seen in ISC. When stakeholders in an ISC operate with a strong sense of relational coordination, power dynamics are less likely to disrupt collaboration because the focus shifts to achieving common goals through mutual understanding and respect. This theory underscores the idea that when relationships are built on a foundation of shared objectives and mutual respect, the influence of power imbalances is diminished. Stakeholders are more likely to engage collaboratively, rather than competitively, leading to more equitable and effective outcomes.

### Implications for practice and future research

#### Equitable resource allocation mechanisms

- Establish multi-sectoral funding boards: Create governance bodies with representation from health, education, social welfare, and civil society to allocate resources using transparent, criteria-based frameworks (e.g. Brazil’s participatory budgeting model) (Victora et al. 2011). Prioritize sectors historically marginalized in donor-driven vertical programmes (e.g. education in HIV/AIDS initiatives).
- Mandate resource equity audits: Regularly assess funding distribution across sectors using metrics such as per-capita allocations or alignment with local disease burdens. Publicly report findings to ensure accountability (Hafner and Shiffman 2013).

#### Context-adaptive leadership models

- Implement rotating leadership roles: In multisectoral committees, rotate leadership positions among sectors to prevent dominance by historically powerful actors (e.g. Uganda’s committee structures) (Bakiika et al. 2023).
- Adopt blended leadership frameworks: Combine hierarchical oversight (e.g. government-led accountability) with participatory decision-making. For example, designate a central coordinator to streamline processes while convening regular stakeholder forums for adaptive problem-solving (Uhl-Bien et al. 2007, Gilson et al. 2023).
- Train leaders in complexity-sensitive approaches: Develop capacity-building programmes focusing on negotiation, conflict mediation, and systems thinking to equip leaders for dynamic ISC environments (Belrhiti et al. 2020).

#### Institutionalizing role clarity

- Develop standardized role templates: Create sector-specific mandates outlining responsibilities, decision-making authority, and accountability mechanisms (e.g. India’s stunting reduction programme) (Kuruvilla et al. 2018). Disseminate these through memoranda of understanding signed by all stakeholders.
- Establish conflict resolution protocols: Designate neutral third-party mediators (e.g. academic institutions or civil society organizations) to resolve intersectoral disputes, ensuring conflicts do not derail collaboration (Fisher et al. 2011).

#### Trust-building and governance structures

- Launch digital transparency platforms: Publish real-time data on resource flows, decision-making processes, and programme outcomes to reduce mistrust (e.g. South Africa’s health information systems) (Gilson and Daire 2011).
- Formalize informal networks: Integrate community leaders and NGOs into governance structures through statutory roles (e.g. Ghana’s district health committees) to ensure sustained engagement beyond individual relationships (Ataguba et al. 2011).

#### Power-sensitive evaluation metrics

- Develop ISC performance dashboards: Track metrics such as sectoral representation in decision-making, frequency of cross-sector consultations, and equity in resource distribution (Sridhar and Woods 2013).
- Incorporate relational indicators: Use validated tools like relational coordination surveys (Gittell 2006) to measure trust, communication quality, and mutual respect among stakeholders.

### Future research priorities

To advance the evidence base on intersectoral ISC in LMICs, future research should prioritize several critical areas. First, operationalizing leadership in fragile contexts demands rigorous exploration of how blended models—combining hierarchical oversight with adaptive problem-solving—can be effectively implemented in settings with weak governance. This includes examining the role of informal leaders, such as community elders or grassroots organizers, in sustaining collaboration during crises, where formal structures may falter. Second, longitudinal studies tracing the evolution of power asymmetries over multi-year ISC initiatives are essential to identify interventions that sustain equity, particularly in resource-constrained environments. Third, institutionalizing role clarity requires deeper investigation into governance mechanisms—such as legislative reforms or intersectoral task forces—that codify mandates and accountability across sectors, ensuring partnerships remain both equitable and operationally viable. Fourth, crisis-responsive ISC warrants attention: research must dissect how pandemics, conflicts, or climate emergencies reshape power dynamics and what adaptive strategies emerge, offering lessons for resilience in volatile contexts. Finally, the nuanced relationship between NGOs and states merits scrutiny, particularly under what conditions NGOs enhance state stewardship through technical expertise or community engagement, versus inadvertently undermining governance through parallel systems or resource diversion. Addressing these gaps will strengthen theoretical and practical frameworks for navigating power in ISC, ultimately fostering more inclusive and sustainable health systems.

### Strengths and limitations

This synthesis demonstrates several significant strengths. First, it has developed six comprehensive PTs, offering a robust framework for understanding power dynamics within ISC. This framework is firmly grounded in empirical evidence from a variety of studies conducted in LMICs, thereby providing valuable, real-world insights into the practical challenges and successes associated with ISC. The evidence-based nature of this synthesis substantially enhances the validity and credibility of the conclusions drawn. Additionally, the theory-driven analysis offers nuanced explanations of how power dynamics shape ISC, addressing a critical yet frequently overlooked aspect of collaboration that is pivotal to the effectiveness of health interventions. Moreover, the synthesis offers actionable recommendations for policymakers and practitioners, advocating for inclusive governance, equitable resource distribution, and the cultivation of strong interpersonal relationships to strengthen ISC outcomes.

This synthesis has several limitations. First, the exclusion of non-English language studies, particularly French-language research, may have constrained the geographical scope, limiting insights from regions such as North and West Africa. Future reviews with multilingual inclusion could address this gap. Second, the focus on role clarity primarily examines intra-organizational dynamics, which may not fully capture the complexities of ISC. Applying network governance and systems-thinking approaches could offer a more comprehensive perspective. Finally, while this study critiques collaborative governance frameworks for not fully addressing power asymmetries, it is important to acknowledge their role in fostering multi-stakeholder engagement and policy coherence. Future research should explore how these frameworks can be adapted to better account for power dynamics in ISC. By recognizing these limitations, this study lays the groundwork for future research to expand geographical representation, integrate broader theoretical perspectives, and refine governance frameworks for ISC.

## Conclusion

This synthesis demonstrates that power in ISC is neither static nor unidirectional; it is negotiated through institutional structures, leadership practices, and relational dynamics. By integrating complexity leadership theory, network governance frameworks, and critical insights from LMIC case studies, we provide a nuanced understanding of how to navigate power asymmetries. For LMICs, advancing ISC requires not only technical solutions but also political commitments to equity, adaptive governance, and inclusive dialogue. Future efforts must prioritize context-sensitive strategies that acknowledge power as both a barrier and a catalyst for transformative health systems.

## Supplementary Material

czaf022_Supp

## Data Availability

All data analysed in this study will be included in the publication’s Supplementary material.

## Reflexivity statement

In this study, we have made a conscious effort to ensure that our authorship team is both inclusive and diverse. The team includes three women and two men, spanning multiple levels of seniority from different sectors. Our authors represent five nationalities, with two members affiliated with institutions in LMICs. Two of the authors are experts in realist methodology, while three specialize in various aspects of global health programming and research in India, Malawi, Uganda, and Ireland. Additionally, two authors bring extensive experience in conducting qualitative fieldwork in sub-Saharan Africa. By including diverse geographical contexts, we aim to enhance the depth of our research. Through this collaboration, we hope to reflect our commitment to addressing inequities in global health research and ensure that the voices shaping our work are varied and representative.
