# Supplementary material for: Power dynamics and intersectoral collaboration for health in low- and middle-income countries: a realist review
Source: Health Policy Plan. 2025 Apr 5;40(6):661–83. doi: 10.1093/heapol/czaf022 (PMC12160828; doi:10.1093/heapol/czaf022)
Supplement: czaf022_Supp [file czaf022_supp.zip › Supplementary File 1.docx]

**Supplementary File 1: Data Extraction**

| Author | Year | Publication Type | Aims/Objectives | Setting |
| --- | --- | --- | --- | --- |
| Lizah Nyawira | 2023 | Peer-reviewed article | Understand how the coordination of the health sector affects health system efficiency in Kenya. | LMIC-Kenya |
| Study Participants | Study Design | Findings | Description of how power affects Intersectoral Collaboration | Models/Theoretical Frameworks |
| Health administrative staff | Qualitative | The study discovered that despite having established formal coordination structures within the Kenyan health system, issues like duplication, fragmentation, and misalignment of functions and actions among various actors undermine the effective coordination of the sector. These challenges were observed both vertically, within different levels of the health ministry and county health departments, and horizontally, between governmental bodies and non-state partners. These coordination problems are likely to disrupt the efficiency of the Kenyan health system by raising the costs associated with its functions and impeding the successful implementation of health programs, thereby negatively impacting overall system performance. | The impact of power is evident in the study through the presence of "duplication, fragmentation, and misalignment" of functions and actions within the Kenyan health system. These issues are likely driven by power dynamics among different actors and entities within the system. The challenges of coordination, both vertically and horizontally, point to struggles for control, influence, and authority among ministries, departments, and non-state partners. These power struggles contribute to increased transaction costs, inefficiencies, and compromised implementation of health programs, ultimately affecting the performance of the health system. |  |
| Key messages of the paper | | Contributor |  | |
| Paper emphasises the significance of effective health sector coordination for achieving efficiency in the Kenyan health system. It identifies existing coordination challenges and proposes actionable recommendations to address these issues and enhance overall health system performance. | | Primary | Additional Notes: | |

| CMOC-1 | Context | Mechanism | Outcome | Excerpts from Text |
| --- | --- | --- | --- | --- |
|  | Hierarchical health administration systems | R1: Unclear coordination framework structures and processes and overlapping roles  R2: Stakeholders feel confused about their roles. The partnership and coordination structures, being advisory in nature, serve to inform decisions made by the more authoritative government structures. Stakeholders feel that without clear delineation of roles, intersectoral meetings are wistful | O1: Leading to complexities and inefficiencies in collaborated efforts | The presence of multiple coordinating structures within the health sector, such as the health sector partnership and coordination framework and intergovernmental coordination mechanisms, has led to duplication and inefficiency in coordination efforts. This redundancy in coordinating entities, specifically highlighted by the duplication of actions between the ICCs (Inter-County Coordinating Committees) and the health sector interagency coordinating committee, results in unnecessary repetition and overlapping meetings involving the same actors. This redundancy and overlapping meetings are considered wasteful in terms of time, resources, and effort. The existence of these redundant coordinating structures without clear differentiation of roles and responsibilities creates confusion and undermines the effectiveness of coordination, leading to poor coordination overall. There was duplication in the structures set up to improve co-ordination at the national MOH. Study respondents felt that there was some duplication between the coordinating structures established by the health sec- tor partnership and coordination framework, and inter- governmental coordination mechanisms. Specifically, it was reported that there were instances of duplication of actions by the ICCs and the health sector interagency coordinating committee. This arrangement led to multiple meetings with the same actors which was considered wasteful.  “You find a structure that is established as a technical thematic committee under the intergovernmental structures is almost like the one established as an interagency coordinating committee under the partnership framework. Sometimes you find that the two deal with similar things, but they have to remain separate because the intergovernmental structures are government structures and are decision-making structures, while the coordination and partnership structures may be more of advisory in nature to inform the decisions that the government structures make. Sometimes the players find it repetitive when they have to hold a different meeting for the partnership structure and the intergovernmental structure and yet they are discussing the same things.” (Respondent 6, National level). |
|  | Within a hierarchical health administration system (C), unclear coordination structures and overlapping roles (R1) create confusion among stakeholders. The advisory nature of partnership and coordination entities, which inform decisions by authoritative government structures, leads to perceived wastefulness in intersectoral meetings due to role ambiguity (R2). These challenges result in complexity and inefficiency in collaborative endeavors. | | | |
| Relevant IPT(s) | Confirm/Refute/ Refine* | Suggested Revisions* | Notes* | Relevant IPT(s) |
| IPT 4 | Confirm |  |  |  |

| CMOC-2 | Context | Mechanism | Outcome | Excerpts from Text |
| --- | --- | --- | --- | --- |
|  | Poorly defined structure within the Ministry of Health policies | R1: Lack of clear-cut job definitions and role ambiguity  R2: Makes stakeholders feel frustrated due to the lack of clear job definitions, resulting in role ambiguity. | O1: Leading to conflict between the staff  O2: Delay in decision making  02: Overlapping | Within the MOH, it was reported that the roles and functions of organisational units were inadequately articulated. This led to a lack of clear job descriptions for staff that resulted in duplication of roles and responsibilities at the MOH. This was exacerbated by a high turnover of staff in positions and inadequate handover between staff when role reassignments occur. Inadequate clarity over staff roles was said to result in duplication of activities, contestations among staff, and delays in decision-making, negatively impacting staff performance.  “There are no clear-cut definitions of what you’re supposed to do. You just do the roles as they come. One day you’re a procurement officer, another day you’re an accountant then you are engaging at an international level. Also, there was no handing over. I came from a health facility to this office and therefore needed some sort of orientation as regards the job description. But when I landed there, I was told, this is your office. And I wondered, so what exactly am I supposed to do? I’m still learning.” (Respondent 4, national level). |
|  | Poorly defined structures within Ministry of Health policies (C), coupled with the lack of clear-cut job definitions and role clarity (R1), lead to stakeholder frustration due to the absence of well-defined job roles, resulting in role ambiguity (R2). Consequently, conflicts among staff members arise (O1), delaying decision-making processes (O2). | | | |
| Relevant IPT(s) | Confirm/Refute/ Refine* | Suggested Revisions* | | Notes* |
| IPT 2 & 5 | Confirm |  | |  |

| CMOC-3 | Context | Mechanism | Outcome | Excerpts from Text |
| --- | --- | --- | --- | --- |
|  | Existing government arrangement of functions and operations within various Ministry of Health (MOH) units and vertical disease programs. | R1: Duplication of functions and monitoring and evaluation units within MOH units and vertical disease programs  R2: Stakeholders feel that duplication of functions and monitoring and evaluation units is wasteful and leads to inefficiency in resource utilisation. | O1: Lack of integration and coordination due to the duplication and fragmentation  O2: Overlapping tasks, redundant data collection, and resource wastage, ultimately leading to suboptimal collaboration outcomes. | “Within the MoH, for example, there is the M&E unit for the division of M& E. But if you go to the various programs, divisions, etc, each has their own M& E unit hence duplication of this function.” (Respondent 5, National level).  The duplication of functions within various Ministry of Health (MOH) units and vertical disease programs, as well as among these programs themselves, creates a fragmented environment. This duplication extends to monitoring and evaluation functions, with both the MOH unit and individual vertical programs having their own monitoring and evaluation units. This redundancy is seen as wasteful and leads to resource inefficiency. The fragmentation of health information systems among these programs and between vertical and non-vertical MOH operations further exacerbates the issue. The duplication of efforts and data collection not only wastes resources but also increases the reporting burden on health facilities. This has negative implications for service delivery and quality. Poor collaboration between sectors arises from this situation due to the lack of streamlined and integrated efforts. Each sector operates independently, resulting in overlapping tasks, redundant data collection, and resource wastage. This not only hampers the effective utilisation of resources but also impedes communication, coordination, and a unified approach to health initiatives. In essence, the duplication and fragmentation undermine the ability of different sectors to work together efficiently, leading to suboptimal collaboration outcomes |
|  | The existing government arrangement of functions and operations within various Ministry of Health (MOH) units and vertical disease programs (C) results in the duplication of functions and monitoring and evaluation units within these entities (R1). This redundancy is perceived as wasteful, leading to inefficient resource use (R2). Consequently, poor collaboration between sectors ensues due to a lack of integration and coordination stemming from this duplication and fragmentation (O1). This results in overlapping tasks, redundant data collection, and resource wastage, ultimately leading to suboptimal collaboration outcomes (O2). | | | |
| Relevant IPT(s) | Confirm/Refute/ Refine* | Suggested Revisions* | | Notes* |
| IPT 5 | Confirm |  | |  |

| CMOC-4 | Context | Mechanism | Outcome | Excerpts from Text |
| --- | --- | --- | --- | --- |
|  | Existing coordination of functions among Ministry of Health (MOH) state agencies with health sector regulatory roles. | R1: Multiple contradictory circulars  R2: The multiplicity of regulatory bodies has led to confusion, excessive requirements, and financial burdens for health facilities. Excessive burden faced by health facilities due to multiple assessments, overlapping regulatory requirements, and conflicting regulations from different health regulatory agencies creates ambiguity | O: Conflicts among State Agencies (SAGAs) at the county level and created a need for harmonisation in the regulatory framework | Participants also reported fragmented coordination of functions between the MOH state agencies that had health sector regulatory functions. It was noted that health facilities were subjected to multiple assessments and regulatory requirements from multiple health regulatory agencies, that were sometimes overlapping.  “Some SAGAs have had conflict within the counties. For example, recently we got a circular that the Kenya Medical Practitioners and dentist council (KMPDC) is the only organization that should be regulating facilities. Then shortly, we saw another memo saying the Kenya health professionals oversight authority (KHPOA) is the one that should regulate. The National Cancer Institute is also supposed to regulate cancer treatment centres. The National Health Insurance Fund (NHIF) is also regulating. There’s quite a bit of confusion and hence need for harmonization of this area. The facilities have been complaining that it is too much, every other person is coming with a requirement and requiring them to pay” (Respondent 9, National level).  Participant at the national level points out conflicts among State Agencies (SAGAs) at the county level, citing an example where there were contradictory circulars about which organisation should regulate health facilities. The Kenya Medical Practitioners and Dentist Council (KMPDC), Kenya Health Professionals Oversight Authority (KHPOA), National Cancer Institute, and National Health Insurance Fund (NHIF) are all mentioned as having regulatory roles. This multiplicity of regulatory bodies has led to confusion, excessive requirements, and financial burdens for health facilities. The participant stresses the urgent need for harmonisation in this area to address the prevailing chaos and challenges faced by facilities. |
|  | The ambiguity in coordinating functions within the Ministry of Health (MOH) (C), alongside the emergence of multiple contradictory circulars stemming from numerous regulatory bodies (R1), has engendered confusion, undue demands, and financial strain on healthcare facilities (R2). This complex regulatory landscape fosters uncertainty and subsequently gives rise to conflicts among State Agencies (SAGAs) at the county level, emphasising the imperative need for the harmonisation of regulatory frameworks (O). | | | |
| Relevant IPT(s) | Confirm/Refute/ Refine* | Suggested Revisions* | | Notes* |
|  | Refine |  | |  |

| CMOC-5 | Context | Mechanism | Outcome | Excerpts from Text |
| --- | --- | --- | --- | --- |
|  | Leadership structures within the departments of heath | R1: Frequent leadership changes and disruptive impact of leadership turnover on initiatives  R2: Newly appointed CDOH leaders often have to restart initiatives, leading to disruptions and negatively affecting the coordination of county health department activities | O1: lack of continuity and disrupted initiatives  O2: Impact the department's vision, plans, and service delivery | Departments of Health (CDOHs) encountered recurrent shifts in leadership, coupled with an absence of effective mechanisms to ensure the seamless continuation of projects over time. This scenario resulted in the need for newly appointed CDOH leaders to often recommence initiatives, a phenomenon viewed as disruptive and detrimental to the alignment of activities within the county health departments. The frequent turnover, particularly at the political leadership level, notably among the Chief Executive Committee (CEC) Members and Chief Officers, who hold pivotal roles in departmental management, is a prevailing trend. This leadership turnover, occurring between successive administrations, significantly impacts the coherence of the department's previous vision, strategic plans, and the overall delivery of services. A pertinent illustration is the reproductive health policy, where the initiation of a draft led by the county executive of health (CEC) was abandoned upon the appointment of a new county executive, giving rise to the commencement of a fresh document.  “There’s a high turnover, particularly at the political leadership level. If you look at the CEC Member and the Chief Officer, they are key people in terms of the running of the departments. From one government to the other, there is an obvious change. That affects the department’s previous vision and the plan and that affects the service delivery.” (County Manager 2, County B).  “An example is the reproductive health policy, the county executive of health (CEC) led the development of a draft, but when a new county executive was appointed, that draft thrown away and the development of a new document was started” (County Manager 1, County B). |
|  | Changing leadership structures (C) disrupt and affect the progress of initiatives (R1). New leaders might need to restart projects, causing problems and making it challenging for health departments to work together effectively (R2). This lack of continuity can disrupt plans and impact the department's functioning and service delivery (O). | | | |
| Relevant IPT(s) | Confirm/Refute/ Refine* | Suggested Revisions* | | Notes* |
| IPT 2 | Confirm |  | |  |

| CMOC-6 | Context | Mechanism | Outcome | Excerpts from Text |
| --- | --- | --- | --- | --- |
|  | Partnership framework at the national level | At the county level, coordination mechanisms included the creation of County Health Stakeholders Fora in addition to the prescribed partnership framework.  The engagement of donors in the development of county health department annual work plans, aided by liaison offices and designated staff at both national and county levels, lead to the enhanced coordination. | This led to a reduction in duplication and an increased alignment of development partner activities. | At the national level, the partnership framework aimed to enhance collaboration, although the Monitoring and Evaluation Report indicated incomplete adoption of this framework at the county level in both counties. In addition to the prescribed structures for coordinating partnerships, county governments established County Health Stakeholders Fora as local coordination mechanisms. Challenges were identified in this context. Counties also improved coordination by involving donors in the development of health department annual work plans. The Ministry of Health (MOH) at both national and county levels designated offices and staff for liaison and coordination between donors and the government, aiming to enhance coordination of development partner activities and minimise duplication. A stakeholders' forum was created to foster understanding among partners, aligning their contributions with priority areas, despite the challenges of differing focus areas. The presence of a liaising officer's office aided in coordinating partner activities, while collaborative work plan development clarified responsibilities and funding sources.  While the partnership framework was established at the national level, the MTR reported that it had not been fully adopted at the county level in both counties. In addition to the structures prescribed by the partnership framework to coordinate partnerships at the national and county level, county governments in addition had established County Health Stakeholders Fora as the coordination mechanisms at the county level. Several challenges were however highlighted by study participants. Counties also enhanced coordination by engaging and including donors in the development of the county Department of health’s annual work plans. The MOH at the national level and county departments of health also had an office and staff designated to provide liaison and coordination between donors and the government. These were thought to have increased co-ordination of development partner activities and reduced duplication.  “We have a stakeholders’ forum where we bring on board all partners in order to understand what they’re doing. For example, HIV could be an emerging issue, but the partners are largely skewed towards addressing maternal health. It becomes difficult to for them to support in that area even if it is your need. You may have adequate numbers sup- porting you in one area but lack partner support in another area. We try to know them and help them understand what we expect from them so that then they contribute towards what we want” (County Manager 2, County B).  “We have the office of the liaising officer which coordinates the activities of the partners” (County referral hospital Manager, County A).  “What is helping us is collaborative development of our annual work plans. It is clearly spelt out which activities will be supported by the County Government and those that will be supported by specific partners. If you look at our annual work plan, you will see each activity indicated and costed and the entity responsible.” (County Manager 1, County A). |
|  | The national partnership framework (C), aimed at enhancing collaboration between government and non-government entities, facilitated the inclusion of County Health Stakeholders Fora and donor engagement in annual work plans (R), improved the alignment of activities, and addressed challenges like imbalances in partner focus for more effective collaboration (O). | | | |
| Relevant IPT(s) | Confirm/Refute/ Refine* | Suggested Revisions* | | Notes* |
| IPT 2 | Refine |  | |  |

| Author | Year | Publication Type | Aims/Objectives | Setting |
| --- | --- | --- | --- | --- |
| André Janse van Rensburg et al., | 2018 | Peer-Review Article | To analyse the power in governance processes of public mental health service provision | district‐level service provision in South Africa |
| Study Participants | Study Design | Findings | Description of how power affects Intersectoral Collaboration | Models/Theoretical Frameworks |
| state and non‐state actors | Qualitative case study | collaborative processes were significantly state‐owned, in terms of funding models, administrative and legislative juris- diction, and state hierarchical referral structure.  Fragmentation between the Departments of Health and Social Development was telling in district forums. Resistance to power structures unfolded, some participants sidestepping traditional hierarchies to leverage funding and support. | power dynamics influence collaboration in the district mental health service delivery network by shaping participation, decision-making authority, resource allocation, and the ability of organisations to contribute effectively. The state health system hierarchy, formal rules, resource limitations, and dependencies on funding sources are key factors that shape power dynamics and impact collaboration within the network | Purdy's Framework for Assessing Power in Collaborative Governance Processes |
| Key messages of the paper | | Contributor | Snowball references | |
| The paper highlights the complexities and different facets of power in integrated mental health care in a South African district, adding to growing literature on the social mechanisms that influence collaboration. | | Primary | None | |

| CMOC-7 | Context | Mechanism | Outcome | Excerpts from Text |
| --- | --- | --- | --- | --- |
|  | The hierarchical structure within the state health system that establishes formal authority and governs the relationships between different actors and organisations involved in mental health service delivery | Resource: State Provincial referral policy is not inclusive  Reasoning: Sense of unequal ownership of the programme among the stakeholders | Limits the non-state service providers participation | It was made clear though, that the state holds primary responsibility for mental health care. “Whether they get funded through grants, or through tax increases, or whatever, the work that NGOs do is the state's responsibility. The only reason that they do it is because they do it on behalf of the state. So you can never financially untie yourself from an NGO... (SW_TH)”  The participant's perspective suggests that regardless of the funding source, whether it be grants or tax increments, the activities carried out by NGOs ultimately remain the responsibility of the state. The participant emphasises that NGOs operate on behalf of the state and therefore cannot completely detach themselves from financial reliance on the state. |
|  | When a state policy is not inclusive (R1) in a state with existing hierarchical structures (C), other sectors may feel left out, leading to a sense of unequal ownership among them (R2). This can limit the participation of non-state service providers in the program's implementation (O). | | | |
| Relevant IPT(s) | Confirm/Refute/Refine* | Suggested Revisions* | |  |
| IPT 1 | Confirm |  | |  |
| CMOC -8 | Context | Mechanism | Outcome | Additional Information & Excerpts from Text |
|  | State health system hierarchy: The hierarchical structure within the state health system that establishes formal authority and governs the relationships between different actors and organisations involved in mental health service delivery. | Resource: Non-inclusive policy  Reasoning: (NGOs) feel constrained to provide mental health services in rural areas due to limited autonomy and unavailability of resources. | Unequal participation | NGOs were further heavily dependent on Department of Social Development (DoSD) funding, and Department of Health (DoH) participants seemingly did not engage in this issue and showed reluctance to operate outside of the DoH governing sphere. |
|  | In situations where the state holds primary authority for providing services, including funding to NGOs (R1), NGOs feel constrained and are hesitant to come forward (R2), especially considering the existing hierarchical structures within the state health system (C). This may result in the NGO sector failing to collaborate with the government program for intersectoral mental health care delivery. | | | |
| Relevant IPT(s) | Confirm/Refute/Refine* | Suggested Revisions* | | Notes* |
| IPT 3 | Confirm |  | |  |
| CMOC-9 | Context | Mechanism | Outcome | Excerpts from Text |
|  | Variation in access to resources demonstrated by the different forms of professional capital across the service providers involved in the collaboration. | Resource: Skilled and trained professionals and funding  Reasoning:  Due to lack of resources few NGOs focused on providing basic care such as clothing, housing only.   Financial advantage of few NGOs enables them to provide services that they perceive as being of higher quality compared to state service providers. | Reduced engagement and strained relationships among NGO participants | NGOs varied widely in terms of resources, with one participant stating, “skilled workers equal money, and money is our only drawback” |
|  | When there is a significant disparity in resource access (C), participants with greater resource access, such as well-funded NGOs or professionals with higher expertise (R1), may have more influence in decision-making processes (R2). This power imbalance can affect the active contribution of other participants, leading to strained relationships and reduced intersectoral engagement (O). | | | |
| Relevant IPT(s) | Confirm/Refute/ Refine* | Suggested Revisions* | | Notes* |
| IPT3/IPT4 | Refine |  | |  |
| CMOC -10 | Context | Mechanism | Outcome | Excerpts from Text |
|  | Established relationship and unique position of social workers in leveraging community resources and connections within the community to support | Resource: Social workers presence skills, and expertise necessary to fulfill their role as valuable contributors to the collaborative arrangement  Reasoning: Improved Trust and open communication between the stakeholders | Outcome 1: Conflict resolution and knowledge sharing  Outcome 2: Improved coordination and collaboration | Social workers were valuable role‐players in a collaborative arrangement between the state psychiatric hospital and a specialised mental health NGO. Social workers at the hospital served as gatekeepers for the NGO to specialised services, while social workers from the NGO conducted home visits and provided other community‐based services for the hospital. |
|  | Existing interpersonal relationships within a sector (C) and skills (R1) facilitate open communication, cooperation, coordination, conflict resolution, trust, knowledge sharing, and cross-learning (R2), enhancing conflict resolution and improving collaborative efforts (O). | | | |
| Relevant IPT(s) | Confirm/Refute/ Refine* | Suggested Revisions* | | Notes* |
| IPT 4 | Refine |  | |  |
| CMOC -11 | Context | Mechanism | Outcome | Excerpts from Text |
|  | The hierarchical structure within the state health system | Resource: Legitimacy of providing mental health services  Reasoning: Other sectors’ stakeholders feel disempowered due to lack of expertise to contribute meaningfully within a hierarchical framework. | O-1 Affects decision-making process  O-2 Disengagement | Psychiatrists were identified as particularly powerful in district mental health decision making, due to psychiatry's legitimacy compared with that of social work, psychology, and nursing. In service delivery, the state psychiatric hos- pital had elevated status, which was amplified by serving as a base for psychiatric outreach. NGOs mentioned that the bulk of their clients are discharged patients from the psychiatric hospital, suggesting a level of dependency on the hospital for a client base |
|  | The presence of existing hierarchical structures within the state health system (C) and the legitimacy of experts in providing mental health services (R) may make other sectors feel less competent (R2). This perception can influence decision-making processes (O1) and decrease sectoral engagement (O2) within the state/district health system. | | | |
| Relevant IPT(s) | Confirm/Refute/ Refine* | Suggested Revisions* | | Notes* |
| IPT4 | Refine |  | |  |
| CMOC-12 | Context | Mechanism | Outcome | Excerpts from Text |
|  | Existing communications structures within the state health system | Resource: Formal and informal communications between the actors  Reasoning: Trust among actors gives rise to openness in the communication that brings clarity in the expectations by the state authorities | Increases the engagement between the sectors | Meetings between state and non‐state collaborating partners ranged from informal telephonic contact to regular formal face‐to‐face meetings. The psychiatric hospital organised a yearly catered social event as a way of thanking certain NGOs for their efforts. The most prominent space for contact was a quarterly mental health district forum, held at and paid for by the DoH provincial headquarters. Selected non‐state service providers in the service network were invited and participated. While many state participants felt that this meeting proved an opportunity for collab- oration, private participants seemed less encouraged about the effectiveness of these meetings. Some went as far as to describe the meetings as political grandstanding, having no clear structure, aims, and outcomes, stating: |
|  | Communication structures within the state health system (C), along with formal and informal communication between actors (R1), increase trust among them, leading to clearer expectations from other sectors (R2). This enhanced trust can result in a greater degree of collaboration (O). | | | |
| Relevant IPT(s) | Confirm/Refute/ Refine* | Suggested Revisions* | | Notes* |
| IPT4 | Confirm |  | |  |

| CMOC-13 | Context | Mechanism | Outcome | Excerpts from Text |
| --- | --- | --- | --- | --- |
|  | State health policy context | Resource: Lack of availability of guidelines on cross-sector partnership  Reasoning: Stakeholders feel confused about the objective of programme implementation and their roles and responsibilities in it. | O1: Reduced accountability  O2: Frustration and disengagement | There was a palpable lack of official strategy and awareness about mental illness and approaches to it, across sectors and service providers.  Regarding welfare, there is really an unhealthy conflict between the national departments and the provincial departments. The national department wants more power, which is good and bad, while the provincial guys also cling to their power because they say they want their own thing. |
|  | In the context of state health policy, the lack of available guidelines on cross-sector partnerships (R1) creates confusion among stakeholders about program objectives and their roles (R2). This can lead to decreased accountability (O1) and an increased risk of frustration and disengagement (O2) among those involved in program implementation. | | | |
| Relevant IPT(s) | Confirm/Refute/ Refine* | Suggested Revisions* | | Notes* |
| IPT5 | Confirm |  | |  |

| CMOC-14 | Context | Mechanism | Outcome | Excerpts from Text |
| --- | --- | --- | --- | --- |
|  | Rural settings | Resource: Lack of resources at the local level  Reasoning: Hesitance to collaborate due to lack to capacity | Lack of collaboration | NGOs sought out PHC clinics in their geographical area to access clinical care for clients suffering from mental illness. State facilities in turn referred MHSU for psychosocial aftercare to NGOs.  However, the limited service capacities of NGOs in rural areas were perceived by state service participants as constraints to collaboration. NGOs were further heavily dependent on Department of Social Development (DoSD) funding, and Department of Health (DoH) participants seemingly did not engage in this issue and showed reluctance to operate outside of the DoH governing sphere. It was made clear though, that the state holds primary responsibility for mental health care.  NGOs varied widely in terms of resources, with one participant stating, “skilled workers equals money, and money is our only drawback” (CC_NGO4). A constrained funding environment resulted in some participants using personal resources to keep their organisations afloat. While some NGOs employed mental health professionals, others focused on providing basic care such as clothing, housing, and treatment adherence and were therefore dependent on state facilities for clinical services, as well as public funding. Well‐funded NGOs saw themselves superior to state service providers in terms of quality, cost‐effectiveness, and efficiency, and one stated that “the state does not have the resources. They don't have the money to keep this massive machine going” (CC_NGO3). |
|  | In rural settings (C), limited resources (R1) can lead to NGOs hesitating to collaborate due to capacity limitations (R2), resulting in poor engagement (O). | | | |
| Relevant IPT(s) | Confirm/Refute/ Refine* | Suggested Revisions* | | Notes* |
| IPT5 | Confirm |  | |  |

| CMOC -15 | Context | Mechanism | Outcome | Excerpts from Text |
| --- | --- | --- | --- | --- |
|  | State policy context | Resources: Resource dependency of one sector on another sector  Reasoning: Unavailability of funds disempowers one sector influencing its relationship with other | Creating blockage for optimal collaboration | The schism between state and non‐state spheres was particularly striking, and the relation between the two service domains suggested resource‐based influences, supporting previous indications that the resource‐based power of NGOs significantly influences their relations with state government in South Africa. These dichotomies block optimal collaboration and cooperation and include key barriers to integrated care: professional domain conflicts; power relationships between services and professionals; distrust; vertical relationships with government; differences in expertise, organisational culture and service delivery approaches; bureaucratic structures; unclear roles; and funding mechanisms |
|  | Within the state policy context, (C) when the interdependence of resources between sectors becomes evident, as one sector's reliance on another is influenced (R2)by the unavailability of funds (R), subsequently creating obstacles that hinder the potential for optimal collaboration. (O) | | | |
| Relevant IPT(s) | Confirm/Refute/ Refine* | Suggested Revisions* | | Notes* |
| IPT 3 | Refine |  | |  |

| CMOC-16 | Context | Mechanism | Outcome | Excerpts from Text |
| --- | --- | --- | --- | --- |
|  | NGOs have limited resources/support from the state | Resource: Social workers embedded in community provides access to resources  Reasoning: Closer connection to community due to their presence in rural setting | Collaboration between state and NGO | The discipline of social work was highlighted as a key point of collaboration between service providers. Social workers' embeddedness in and access to community‐based resources was highlighted as a vital point of collaboration with different partners. For example, social workers were valuable role‐players in a collaborative arrangement between the state psychiatric hospital and a specialised mental health NGO. |
|  | With limited support and resources from the state level (C), social workers embedded in the community (R1) establish connections and facilitate collaboration between the state and NGOs (R2), particularly in rural settings (O). | | | |
| Relevant IPT(s) | Confirm/Refute/ Refine* | Suggested Revisions* | | Notes* |
| IPT 3 | Refine |  | |  |

| CMOC-17 | Context | Mechanism | Outcome | Excerpts from Text |
| --- | --- | --- | --- | --- |
|  | Hierarchical structure/state seen as more powerful/in charge vs non-state partners | Resource: NGOs dependent on state for funding and support  Reasoning: State is therefore perceived as the responsible for coordination, etc. | One-sided relationship where action/ collaboration/participation is contingent on the state’s decisions rather than collaboration | Collaborative processes were significantly state‐owned. This is apparent in the dependence of NGOs on state funding, administrative, and legislative support, as well as the hierarchical nature of referral patterns according to levels of state health care. No formal agreements were in place, and collaboration occurred in a piecemeal, informal fashion, dependent on key actors in health facilities to reach out to others to extend the scope of care for MHSU.  The responsibility to initiate and foster collaboration with non‐state service providers was the state's responsibility, both by state and private participants  Meetings between state and non‐state collaborating partners ranged from informal telephonic contact to regular formal face‐to‐face meetings. The psychiatric hospital organised a yearly catered social event as a way of thanking certain NGOs for their efforts. The most prominent space for contact was a quarterly mental health district forum, held at and paid for by the DoH provincial headquarters. Selected non‐state service providers in the service network were invited and participated. While many state participants felt that this meeting proved an opportunity for collaboration, private participants seemed less encouraged about the effectiveness of these meetings. Some went as far as to describe the meetings as political grandstanding, having no clear structure, aims, and outcomes, stating: If you look at what is said in Batho Pele [national patient rights charter] that every person has a right, have a right to best health services that he can get. I go to the Free State mental health meetings, where the police and all that sit and then you have to listen to countless promises and whatever, and I just shake my head. (CC_NGO3) |
|  | The hierarchical state holds more power and control than non-state partners like NGOs (C), as the latter rely on state funding (R1). This dynamic results in the state being perceived as responsible for coordination, given the one-sided relationship where collaboration hinges on state decisions (O). | | | |
| Relevant IPT(s) | Confirm/Refute/ Refine* | Suggested Revisions* | | Notes* |
| IPT 3 | Confirm |  | |  |

| CMOC 18 | Context | Mechanism | Outcome | Excerpts from Text |
| --- | --- | --- | --- | --- |
|  | Lack of mental health information systems- there is no lack of data collection | Resource: No avenues for communication between different providers/sectors  Reasoning: No knowledge/ awareness | Little coordination between providers.  Restricts | In the absence of a unified mental health information system, little or no routine information was gathered and shared among service providers. In the state sphere, one of the only indicators gathered by the district health system is the number of new patients. Little evidence emerged that this was used in planning and governance processes. Furthermore, the infrastructural challenges faced by smaller community‐based NGOs severely restricted their method and frequency of voice, given that often they did not have a telephone, fax, or internet presence, making them dependent on larger NGOs and state mental health actors to access the mental health service network. Information shared among state and private participants mostly involved telephone conversations and email. For instance, a participant at the state psychiatric hospital queried a mental health NGO to follow up on discharged patients requiring additional support, including assistance with financial management, acquiring identification documents, accessing disability grants, and processing curatorship. Some NGOs did not have initial access to the quarterly mental health forum and were dependent on key state participants to be formally invited. As far as could be determined, the dialogue was led by the DoH, and minutes were not circulated. The bulk of private participants had no knowledge of the existence of South Africa's national mental health policy and therefore did not analyse mental health care according to its strategic parameters |
|  | The absence of comprehensive mental health information systems at the state level (C) leads to a lack of communication channels among providers from different sectors (R2). This deficiency arises due to insufficient knowledge and awareness, resulting in limited coordination among stakeholders. | | | |
| Relevant IPT(s) | Confirm/Refute/ Refine* | Suggested Revisions* | | Notes* |
| IPT 2 | Refine |  | |  |

| CMOC-19 | Context | Mechanism | Outcome | Excerpts from Text |
| --- | --- | --- | --- | --- |
|  | Organisational culture and State bureaucratic structures | Resources: Vertical relationships with the government and power relationships between service and professionals  Reasoning: influences the flow of information and communication between sectors and can create mistrust and ambiguity in role clarity among stakeholders | Outcome 1: Creating conflicts  Outcome 2: Lack of participation of small sectors | The division between governmental and non-governmental sectors is notable, with resource-driven dynamics impacting their interaction. This reinforces the idea that the resource-based influence of NGOs plays a substantial role in their connections with the South African government. These divisions hinder effective teamwork and encompass obstacles to integrated care, encompassing conflicts within professions, power dynamics, distrust, interactions with government, divergent expertise and cultures, bureaucratic frameworks, uncertain roles, and funding mechanisms. |
|  | Within existing organisational and bureaucratic structures (C), vertical relationships with government entities (R1) result in a power imbalance, influencing information dynamics and fostering mistrust and role ambiguities (R2). These factors collectively contribute to intersectoral conflicts (O1) and discourage involvement of smaller sectors (O2). | | | |
| Relevant IPT(s) | Confirm/Refute/ Refine* | Suggested Revisions* | | Notes* |
| IPT 4 | Refute |  | |  |

| Author | Year | Publication Type | Aims/Objectives | Setting |
| --- | --- | --- | --- | --- |
| Denice Kamugumya | 2016 | Peer-Review Article | To understand power distribution and the interests of local actors to engage non-state actors (Public-Private Partnership). | District level-Tanzania |
| Study Participants | Study Design | Findings | Description of how power affects Intersectoral Collaboration | Models/Theoretical Frameworks |
| Key informants identified through stakeholder mapping. | Qualitative case study | The study highlights power differences between state and non-state actors which is determined by the weak capacity of governing bodies to monitor non-state actors, which is acerbated by weak accountability linkages.  The study shows that non-state actors are seen to be severely marginalised in district strategic planning.  private sector representation lacks authenticity as power influences the selection of non-state actor representatives at district-level committees.  Limited inclusion of non-state actors in planning and decision-making. | Uneven power between stakeholders impedes the inclusiveness of the private sector in district strategic planning.  Power dynamics influence coordination between state and non-state actors at the district health service delivery.  Power dynamics influence decision-making, planning and monitoring of health services by state and non-state actors. Power dynamics-controlled accountability and decision-making processes in order to achieve health goals  Power also is seen to impact participation and representation of stakeholders. | Decision-space framework. |
| Key messages of the paper | | Contributor |  | |
| The study shows how power dynamics influences policy implementation at a local level. It shows the capacity of local government officials to make choices that would embrace strategic plans in health care service delivery. The study also highlights the need of integrative collaborations of various sectors to achieve the optimum result of health care service delivery | | Primary | None | |

| CMOC-20 | Context | Mechanism | Outcome | Excerpts from Text |
| --- | --- | --- | --- | --- |
|  | District council teams and governing bodies at different hierarchical levels | R1- Council health management team having skilled professionals and resources  R2: Counsel team’s inclination towards public facilities has channelised more resources to public facilities leading to frustration among private providers for being neglected and feel less powerful compared to government sector | Poor engagement of non-state actors in PPP Model | The District Council Team is very strong and makes executive decisions; however, it is more inclined towards the public than the private sector. As a result, the Council Team directs the CHMT and CHSB, which also has some CHMT members as board members, to focus on public facilities. The Council Team focuses more on how resources are channeled to public facilities in order to win voters’ popularity rather than on strategies that would im- prove equity, efficiency and quality of care. The CHSB has the potential to foster the engagement of non-state actors, however the CHSB is weak as it lacks resources for effective PPP implementation: |
|  | Health governing bodies at different levels (C) favor public facilities in terms of resources (R1) and neglect integrating private providers, leading to frustration and a sense of powerlessness (R2), resulting in poor private sector engagement in the PPP model. | | | |
| Relevant IPT(s) | Confirm/Refute/ Refine* | Suggested Revisions* | | Notes* |
| IPT 2 & 3 | Confirm |  | |  |

| CMOC-21 | Context | Mechanism | Outcome | Excerpts from Text |
| --- | --- | --- | --- | --- |
|  | Weaker ward health committees | R1- Limited understanding of PPP strategy  R2: Private sector providers do not trust ward health committees due to poor implementation skills of PPP strategy and less power in decision-making at policy level | Leading to Poor engagement of non-state actors in PPP Model - 02  Lack of opportunity to engage – Outcome 1 | There are wards where the Ward Health Committees do not exist, and in other wards the Ward Health Committees do exist but are weak in terms of the PPP policy implementation as they have limited understanding of PPP as a concept or strategy:  “I would say our committee (Ward Health Committee) is non-functional, completely non-functional since last year. We tried to revive it but there were complaints among members about general skills development” (Participant 20 – Bagamoyo 2014). |
|  | Weaker ward health committees at the local level (C), due to limited understanding of PPP strategies and lower decision-making power at higher levels (R1), lead to a lack of trust from private providers, causing frustration and poor engagement in PPP strategies (O). | | | |
| Relevant IPT(s) | Confirm/Refute/ Refine* | Suggested Revisions* | | Notes* |
| IPT 4 | Confirm |  | |  |

| CMOC-22 | Context | Mechanism | Outcome | Excerpts from Text |
| --- | --- | --- | --- | --- |
|  | Non-inclusive Council Policies | R1: Inadequate involvement of the private sector at the stage of strategic decision-making  R2: Creating a sense of unfair treatment among private sector providers. Inauthentic engagement- purposeful engagement of old/Lack of meaningful engagement- inauthetntic engagement | Leading to poor participation | It was revealed by key informants that the CHMT pre- pares the Comprehensive Council Health Plan (CCHP) annually that has to be endorsed by the District Council Team before being approved by the Prime Minister‘s Of- fice Regional Administration and Local Government (PMO-RALG) and the MoHSW. All relevant policy guide- lines are provided by the MoHSW. However, it was ob- served in this research that the private sector is not adequately represented in strategic decisions. The CHSB is not directly involved in strategic planning. Those whose names appear in the CCHP document as representatives of non-state actors did not actually participate in strategic sessions, reflecting inadequate inclusion of all services providers. These were some responses from individuals whose name appears on the CCHP document as a repre- sentative of non-state actors:  “I used to attend strategic meetings back in early 2000s as a representative of (non-state actors) but after I took another job I stopped. So if you tell me I am still a representative, that is not right, I don’t attend those meetings...” (Participant 1 – Bagamoyo 2014).  “I usually hear about it (CCHP). I have never seen a copy...I don’t understand why my name should appear in that document as a representative” (Participant 9 – Bagamoyo 2014). |
|  | Non-inclusive council policies (C) exclude private providers (R1), generating a sense of unequal treatment and reducing private sector participation in PPP program implementation (O). | | | |
| Relevant IPT(s) | Confirm/Refute/ Refine* | Suggested Revisions* | | Notes* |
| IPT 5 | Refine |  | |  |

| CMOC-23 | Context | Mechanism | Outcome | Excerpts from Text |
| --- | --- | --- | --- | --- |
|  | Unsupportive govt structures and polices by govt | R1: Unavailability of enough staff at private health facilities and high number of cases to attend  R2: Healthcare staff at private facilities feel frustrated and overburdened | Limits the participation in PPP implementation | “The government needs to find a way to support us. They (government) see our monthly reports. In a month, a number of under-fives attended here is between 1000 and 2000, new cases of pregnant mothers is between 70 and 80, leave alone ‘re-attendance’. For family planning, we report 200 clients. That is a very tough job...We really struggle to get staff. And I understand even at government facilities there is a shortage, but there are ways we can work together on this” (Participant 21 – Bagamoyo 2014). |
|  | Unsatisfactory government policies and structures (C) and overworked healthcare staff in private facilities (R1) cause frustration (R2) and limit private sector engagement in PPP implementation (O). | | | |
| Relevant IPT(s) | Confirm/Refute/ Refine* | Suggested Revisions* | | Notes* |
| IPT 3 | Confirm |  | |  |

| CMOC-24 | Context | Mechanism | Outcome | Excerpts from Text |
| --- | --- | --- | --- | --- |
|  | Poor performance review accountability structures | R1: Poor articulation of health outcomes and lack of feedback mechanism  R2: Due to ambiguity in open communication, providers feel confused about their contribution to success or failure | Leading to a loss of interest in the participation of PPP strategy | he CCHP does outline health outcomes that need to be collectively achieved in the district. However, there is a weak accountability linkage structure among actors (Table 1), which is limited to supplying information creating a room for non-compliance. More emphasis is given to individual service’s entry to the market through registration and licencing.  “There is no feedback provided once we have submitted our reports. We don’t discuss anything further. When you see the performance of Bagamoyo District as a whole, you just assume you were part of the success/ failure...and it is only when you are lucky to attend higher level meetings” (Participant 3 – Bagamoyo 2014). |
|  | Inadequate review mechanisms and accountability structures in PPP policy (C), with improper or no feedback mechanism (R1), create unsupportive conditions for open communication between sectors,(R2) leading to disinterest in PPP participation. (O) | | | |
| Relevant IPT(s) | Confirm/Refute/ Refine* | Suggested Revisions* | | Notes* |
| IPT 5 | Confirm |  | |  |

| CMOC-25 | Context | Mechanism | Outcome | Excerpts from Text |
| --- | --- | --- | --- | --- |
|  | Unsupportive policy structures or guidelines | R1: Unavailability of MOU guidelines and role clarity  R2: creates confusion and misunderstanding between state and non-state providers | Leading to unsynchronised engagement | It was noted that a lack of written agreement at the service level contributes to misunderstandings between parties, and there are service interruptions that are experienced as a result. This was the case with the Prevention of Mother-to-Child Transmission of HIV (PMTCT) service provision where private providers receive subsidised Antiretroviral (ARV) drugs and reagents from the government. In order to receive these supplies private providers have to submit their reports monthly, something that is not done consistently. This has created an un-healthy relationship between parties as there are those government staff who strictly adhere to this requirement, and others who issue ARV drugs and reagents without adhering to this requirement:  “With private providers, there is a challenge as there are those who provide incomplete data, and those who do not respond. For example data on HIV, we need that information as they (private providers) get subsidised reagents from the government, but you will find that they (private providers) provide incomplete information claiming that they buy other items using their own resources” (Participant 8 – Bagamoyo 2014). |
|  | Absence of policies or guidelines (C) clarifying the roles of non-state sector providers (R1) results in confusion and misunderstanding between sectors, leading to disjointed engagement.(O) | | | |
| Relevant IPT(s) | Confirm/Refute/ Refine* | Suggested Revisions* | | Notes* |
| IPT 5 | Refine |  | |  |

| CMOC-26 | Context | Mechanism | Outcome | Excerpts from Text |
| --- | --- | --- | --- | --- |
|  | Hierarchical and dominated district health policies | R1: Unauthentically represented non-state actor representatives in the planning document  R2: Makes poor participation of non-state actors and they feel left from the decision-making process | Biased decision making | Much effort is being made to promote PPP and the private sector at the district level in Bagamoyo. However, findings from this study highlight that these efforts are not well-coordinated, and effective mechanisms have not been established as yet to tap resources from or for the private sector. Non-state actors are seen to be severely marginalised in district strategic planning.  The presence of non-state actor representatives’ names in the district strategic planning documents that are not authentically represented, brings to light the characteristics of actors who govern the decision-making process. As argued by ‘public choice’ theorists, this reveals the self-interest driven behaviour of local government officials and the use of ‘power as thought control’ to influence decisions of executive meetings at the district and national levels. Core elements for PPP such as inclusion, transparent, and ethical behaviours remain under-recognised |
|  | Hierarchical and non-transparent district health policy structures (C) exclude authentic representation of non-state actors, (R1) resulting in poor involvement in decision-making processes and unequal resource allocation, (R2) leading to waning interest in partnerships. (O) | | | |
| Relevant IPT(s) | Confirm/Refute/ Refine* | Suggested Revisions* | | Notes* |
| IPT 3 and 4 | Refine |  | |  |

| CMOC-27 | Context | Mechanism | Outcome | Excerpts from Text |
| --- | --- | --- | --- | --- |
|  | Politically motivate interests | R1: Resource allocation and distribution  R2: Makes district health council feel powerful and allow selective involvement of partner in decision making process | Leading to poor engagement of non-state actors  Ineffective resource allocation 0-2 | Local governments are expected to have more decision-making power on how funds are spent and with what type of provider [4]. However, this study reveals uneven power distribution between governing bodies at the district level. The CHMT is equipped with strong personnel with biomedical skills, and other resources, but perceives PPP narrowly. The District Council Team is powerful and tends to influence decisions made by CHMT and CHSB, however it is inclined to place politically motivated interests over value distribution, which has been argued to affect the engagement of non-state actors [11]. Study findings highlight limited skills on al- locative efficiencies given competing priorities to im- prove systems performance through PPP. |
|  | If government interests are politically influenced (C), councils gain more power (R2) in allocating resources to state and non-state actors (R1), resulting in weak engagement of non-state actors in the PPP strategy (O). | | | |
| Relevant IPT(s) | Confirm/Refute/ Refine* | Suggested Revisions* | | Notes* |
| IPT 3 and 4 | Confirm |  | |  |

| CMOC-28 | Context | Mechanism | Outcome | Excerpts from Text |
| --- | --- | --- | --- | --- |
|  | Existing relationship structures within district health councils | R1: Gaps in information sharing, non-transparent information sharing mechanisms  R2: No state actor’s perception of poor shared values and mistrust on policies | Leads to poor representation of non-state actors | Study findings here indicate a wide gap in information sharing as the relationship that is seen is that of ‘regulator and regulated type’ [16]. Relational elements dynamics such as commitment to shared values, high level of trust and interactive problem-solving are not perceived as centrally important by the local government officials in district planning and are exacerbated by the lack of dialogue with non-state actors. It has been highlighted that relational element, when they complement other governance mechanisms, are likely to yield desirable outcome |
|  | District health councils with poor communication structures (C) create confusion due to non-transparent (R1) communication and reduce trust in the engagement policies of govt leading to poor representation of non-state actors in PPP implementation | | | |
| Relevant IPT(s) | Confirm/Refute/ Refine* | Suggested Revisions* | | Notes* |
|  | Refine |  | |  |

| Author | Year | Publication Type | Aims/Objectives | Setting |
| --- | --- | --- | --- | --- |
| F. A. Asaag | 2021 | Peer-Review Article | To explore the facilitators of and barriers to successful convergence between the human, animal and environmental health sectors in India. | LMIC (India): National and state level; multi-sectoral. |
| Study Participants | Study Design | Findings | Description of how power affects Intersectoral Collaboration | Models/Theoretical Frameworks |
| Key informants | Mixed methods | Key barriers to successful cross-sectoral collaboration for zoonotic disease control include the absence of supportive policies, conflicting priorities across multiple ministries, and limited institutional capacities. The response to zoonotic diseases is influenced by a politicised and hierarchical environment that shapes the roles of various actors and collaborative outcomes. The challenge is exacerbated by the fragmentation of ministries responsible for human and animal health, each with distinct affiliations and goals, hindering effective convergence. Additionally, the intricate health system complexity arises from variations in state-level health administration and capacities for both animals and humans, significantly impacting health policy decisions and overall outcomes. | Power dynamics affect innumerable factors that operates to either constrain or facilitate the success of cross-sectoral convergence at different stages i.e. information-sharing, undertaking common activities and merging resources and infrastructure. Priorities of the ministries, policies, resources impact partnerships that is multi-sectoral in nature. |  |
| Key messages of the paper | | Contributor |  | |
| The paper highlights the importance of strengthening existing national policy frameworks as a first step for leveraging cross-sectoral capacity for improved disease surveillance and interventions. | | Primary | None | |

| CMOC-29 | Context | Mechanism | Outcome | Excerpts from Text |
| --- | --- | --- | --- | --- |
|  | Existing political structures and lack of availability of guidelines on ISC | R1: Sectoral affiliation, no goal alignment  R2: Decision making process gets becomes politically driven rather than technical | Ambiguity in role clarity | Challenges associated with achieving cross-sectoral convergence due to fragmentation and the presence of distinct affiliations within different sectors. The various ministries and departments involved often have divergent goals and power dynamics. The absence of comprehensive guidelines for coordinating collaboration among these sector agencies and departments exacerbates the issue. Decision-making in this context is driven more by political considerations rather than being solely technical in nature. This suggests that the lack of clear guidelines and the influence of political factors make it difficult to establish effective coordination between different sectors, resulting in conflicting policies and challenges in achieving alignment.  ““There are always points of conflicting policy as there is no comprehensive guidelines on synergising coordination between sector agencies and departments. The decision-making is almost always political as it is technical ... ” (Interview 7, Public Health). “ |
|  | The lack of guidelines on convergence (C) leads to sectoral isolation and misaligned goals (R1), influencing decision-making processes (R2) more politically than technically, resulting in ambiguity and poor participation from other sectors (O). | | | |
| Relevant IPT(s) | Confirm/Refute/ Refine* | Suggested Revisions* | | Notes* |
| IPT 5 | Confirm |  | |  |

| CMOC-30 | Context | Mechanism | Outcome | Excerpts from Text |
| --- | --- | --- | --- | --- |
|  | Existing government bureaucracy structures | R1: Perception of being silos  R2: Stakeholders fail to think collectively to address the health issues | No goal alignment affecting ISC | Effectiveness of cross-sectoral collaboration is hindered by the inherent characteristics of various government agencies representing different disciplines. These agencies are structured in a way that promotes isolation or working independently, like silos, without effective communication or cooperation with other sectors. This isolation is attributed to the bureaucratic nature of these agencies, where each department tends to believe that it possesses all the necessary expertise within its domain. This situation creates a challenge for promoting collaboration and integration between sectors, as the agencies are inclined to operate within their own boundaries without seeking expertise or input from other areas.  Cross-sectoral collaboration is hampered by the very nature of the different disciplinary government agencies. So, they are designed to be silos. They don’t work well with reaching out to others. This is to do with the nature of the bureaucracy, where each department thinks that all expertise lies within it. That’s one challenge ... ” (Interview 3, Environment). |
|  | Complex government bureaucracy structures (C) and policies can lead to a perception of sectoral isolation, inhibiting collective thinking and resulting in poor sectoral convergence. | | | |
| Relevant IPT(s) | Confirm/Refute/ Refine* | Suggested Revisions* | | Notes* |
|  | Refine |  | |  |

| CMOC-31 | Context | Mechanism | Outcome | Excerpts from Text |
| --- | --- | --- | --- | --- |
|  | Hierarchical health system structures | R1: Absence of feedback mechanism  R2: Sectors do not open up about their challenges in working together | Affecting the collaborated action | The hierarchical structure within the health system is identified as a key factor that weakens these feedback loops. As a result, there is a lack of effective communication between individuals at different levels of authority, such as those at the ground level and those in higher positions in Delhi and state capitals. Ground-level individuals may hesitate to provide feedback or raise concerns to their superiors due to perceived challenges or fears. This lack of open communication and feedback can prevent critical information from reaching decision-makers and limit their understanding of the actual conditions and challenges faced on the ground, ultimately affecting the responsiveness and adaptability of the health system.  “Because of the hierarchy, the (health system’s) self-feedback mechanisms are very weak. So very rarely do people sitting in Delhi and in state capitals listen to what is happening on the ground level. Very rarely do ground-level people have the courage or bravery to give feedback to their seniors ... ” (Inter-view 3, Environment). |
|  | Hierarchical health system structures (C) often lack a feedback mechanism (R1), which discourages sectors from openly discussing challenges (R2), leading to weakened collaboration (O). | | | |
| Relevant IPT(s) | Confirm/Refute/ Refine* | Suggested Revisions* | | Notes* |
|  | Confirm |  | |  |

| Author | Year | Publication Type | Aims/Objectives | Setting |
| --- | --- | --- | --- | --- |
| Aloysius Ssennyonjo | 2022 | Peer-reviewed article | Explores the nature of coordination instruments for multisectoral action at the national level in Uganda and the complexities of how these tools play out in implementation. | Uganda |
| Study Participants | Study Design | Findings |  |  |
| Govt and non govt officials | Qualitative | This study demonstrated that a contextualised examination of specific coordination tools can be enhanced by delineating the underlying institutional forms of ideal-type mechanisms | | |
| Key messages of the paper | | Contributor |  | |
| The research article explores the nature, implementation dynamics, and functioning of coordination instruments for multisectoral action in Uganda's national context. It underscores the need for a nuanced understanding of these tools and their interplay within government systems. | | Primary | None | |

| CMOC-32 | Context | Mechanism | Outcome | Excerpts from Text |
| --- | --- | --- | --- | --- |
|  | Structures of authority in governance  Resource: Autocrat leadership (Context) coz not offered by programme | Resource: Autocrat leadership (Context) coz not offered by programme  Reasoning: stakeholder feel constricted to act upon thematic areas due to over-centralised power | Suboptimal participation | The findings report on the hierarchical structure within the Cabinet of a government. It mentions that most respondents observed a strong hierarchy within the Cabinet, where some Cabinet ministers held more significant positions of political leadership over sectoral MDAs (Ministries, Departments, and Agencies), while others, such as junior ministers and ministers responsible for specific population groups or geographical regions, had less authority and power. It also highlights that a few individuals noted that even these Cabinet ministers were constrained by the considerable authority of the President. In other words, the President held substantial power and influence over the Cabinet's activities. Additionally, some activities carried out within the ministries were closely linked to the President's office. The inclusion of the development partner's remark suggests that this hierarchical structure and the influence of the President were also observed and noted by external actors involved in the country's development or governance processes. Overall, it provides insights into the power dynamics and hierarchical nature of the government's decision-making processes. One development partner further remarked as follows:  The power in the Cabinet is overcentralised. The President is too powerful. That is why we do not have standing cabinet committees. We have a lot of ad-hoc committees, which is not good. When you have standing committees, they allow you to disperse power to constituencies that will take an interest in thematic issues (NSA-1). |
|  | In a hierarchical governance structure (C) characterised by autocratic leadership, (C) stakeholders are notably constrained in their ability to actively engage (R2) in addressing thematic issues owing to the excessive centralisation of power and the suboptimal participation of crucial sectors. (O) | | | |
| Relevant IPT(s) | Confirm/Refute/ Refine* | Suggested Revisions* | | Notes* |
| IPT 3 | Refine |  | |  |

| CMOC-33 | Context | Mechanism | Outcome | Excerpts from Text |
| --- | --- | --- | --- | --- |
|  | Existing socio-political structures -conducive  Break it down- be specific-  Health system structures  National health policy context  --- Re Read--- | R: Platform to discuss and approve ISC policies and capacity building  R2: These platforms promote open communication and capacity building sessions brings in collective responsibilities among sectors | Coordinated action | The Cabinet in this context functions as a network-based mechanism, characterised by collective responsibility. It acts as a central platform for discussing and approving government priorities and policies, including those spanning multiple sectors. Internal Cabinet structures utilise network-based methods and 'soft power,' such as capacity building, to foster a common understanding of policymaking processes. This approach is confirmed and practised by a senior official, emphasising its significance in the government's decision-making procedures. One senior official affirmed  We also go there to do capacity building. We also move out to train them (MDAs) on practically how they do the coordination of policy formulation and implementation. Like from tomorrow, we are meeting the prison people. Last week, we met the information, communication, and national guidance ministry.—MDA-2 |
|  | If the existing health system structures are conducive ( C ) the platform established for the discussion and approval of ISC policies,(R1) combined with capacity building initiatives, (R1) plays a pivotal role in fostering open communication and instilling a sense of collective responsibility among sectors, (R2) ultimately driving coordinated action (O) | | | |
| Relevant IPT(s) | Confirm/Refute/ Refine* | Suggested Revisions* | | Notes* |
| IPT 4 | Refine |  | |  |

| CMOC-34 | Context | Mechanism | Outcome | Excerpts from Text |
| --- | --- | --- | --- | --- |
|  | administrative structures | Government approach as one homogeneous entity leading to internal silos within sectors  Pull out more in mechanism | Leading to poor sectoral collaboration  People working in silos- because they could be focused, competing interest, no guidelines, lack of leadership, binding authority | When administrative structures are consolidated and treated as if they constitute a singular, uniform entity, it sets the stage for certain adverse outcomes. Specifically, this approach leads to the formation of what are referred to as "internal silos" within different sectors of the government. These internal silos represent isolated units or compartments within the government where each sector primarily focuses on its own concerns and responsibilities, often to the exclusion of effective communication and collaboration with other sectors. As a result of these internal silos, the collaboration between sectors, which is essential for addressing complex and interconnected challenges, diminishes significantly. This decline in sectoral collaboration is one of the immediate consequences (O1) and has broader implications for governance as a whole. The overall effectiveness of governance is adversely affected (O2) because, when different parts of the government operate in isolation and fail to work together cohesively, the ability to implement policies, deliver services efficiently, and respond effectively to multifaceted issues becomes severely compromised.  One respondent observed:  “you know refugees fall under a ministry in OPM, ministry of disaster and refugees, so there are certain things one can- not do. One cannot go to <name of Minister of disasters> (to brief him) because they do not work under the ministry of refugees. So, the issue of mandate actually can also be a bottleneck.—MDA-7 |
|  | Rewrite: When administrative structures are unified under a government approach treated as a single homogeneous entity, (C) it fosters the development of internal silos (M) within sectors, subsequently resulting in a decline in sectoral collaboration (O1) and overall impaired governance effectiveness. (O2) | | | |
| Relevant IPT(s) | Confirm/Refute/ Refine* | Suggested Revisions* | | Notes* |
| IPT 3 | Confirm |  | |  |

| CMOC-35 | Context | Mechanism | Outcome | Excerpts from Text |
| --- | --- | --- | --- | --- |
|  | Existing hierarchical structures and National Coordination Policy | R1: Technical coordination committees  R2: Infrequent meetings of the Technical Implementation Coordination Committee and concerns about having a large number of members in the committee made its operation difficult, possibly leading to issues related to communication, decision-making, and overall efficiency. | Obstacles to achieving effective coordination and collaboration across government entities. | The policy aimed to achieve several objectives, including (1) enhancing collaboration and coordination among various Ministries, Departments, and Agencies (MDAs), (2) establishing standardised reporting procedures, and (3) strengthening the secretariats of Sector Working Groups (SWGs). However, the finding also highlights a significant issue concerning the implementation of the Integrated Framework for Coordination of Priority Programmes and Initiatives (IFCPPI). It is noted that the operationalisation of this framework had not been very effective. The finding also mentions that a government official expressed concerns about membership and efforts to rectify the situation and streamline participation. This suggests that there were challenges related to the membership composition of these coordination structures and ongoing efforts to improve and make them more efficient.  ...apparently this committee (TICC) was too big at that time. Its membership is very big. So, they were trying to see how they do not call anyone and then have nothing to talk about that affects them—MDA-8  The statement is explaining a challenge related to the Technical Implementation Coordination Committee (TICC), which is a committee responsible for implementing certain initiatives or programs. According to the source, at the time in question, this committee had a large number of members, suggesting that it was quite extensive in its composition.  The findings outlines the development of the National Coordination Policy and its objectives, while also highlighting issues related to the effectiveness of the Integrated Framework for Coordination of Priority Programmes and Initiatives (IFCPPI), particularly the infrequent meetings of the Technical Implementation Coordination Committee (TICC) and concerns regarding membership and participation. These issues suggest potential obstacles to achieving effective coordination and collaboration across government entities. |
|  | In a hierarchical governance structure (C), if the coordination committees (R1) are large and face hurdles such as infrequent committee meetings, this leads to reduced efficiency, communication problems, and decision-making challenges (R2), ultimately resulting in reduced overall effectiveness, which impedes effective coordination and collaboration across government entities (O). | | | |
| Relevant IPT(s) | Confirm/Refute/ Refine* | Suggested Revisions* | | Notes* |
| IPT 3& 5 | Confirm |  | |  |

| CMOC-36 | Context | Mechanism | Outcome | Excerpts from Text |
| --- | --- | --- | --- | --- |
|  | Existing bureaucracy structures | R1: Planning authority  R2: Planning authority with its positionality within the bureaucracy structures leverages its authority and convening power to facilitate inter-ministerial collaboration due to which actors actively get involved | (O) Facilitate collaboration among government ministries, shape policy definitions, clarify roles, and ensure financial feasibility | The National Planning Authority (NPA) plays a pivotal role in facilitating effective coordination and policy development across various government agencies. Respondents have reported that the NPA occasionally employs its influence and authority to assist other government bodies in performing their coordination roles more effectively. For instance, the NPA harnesses its convening power, which means it can bring together relevant stakeholders, to lead an inter-ministerial committee. Within this committee, the NPA has undertaken several crucial tasks:   Defining Universal Health Coverage (UHC): The NPA has been instrumental in shaping the concept of UHC as a comprehensive goal that transcends various government sectors. This definition emphasises the importance of considering social determinants of health when crafting policies related to healthcare access.  Clarifying Actor Roles and Performance Indicators: The NPA has contributed to clarifying the roles and responsibilities of different actors involved in achieving UHC. It has also played a part in establishing performance indicators or metrics to assess how effectively these actors are carrying out their roles. This ensures that there is clarity and accountability in the implementation of UHC-related policies.  Determining Intervention Costs: The NPA has been involved in the financial planning aspect by calculating the costs associated with implementing interventions aimed at achieving UHC. This financial assessment is critical for allocating resources effectively and ensuring the sustainability of UHC initiatives.  Importantly, an NPA official has confirmed their agency's commitment to leading the coordination of multisectoral policies related to universal health coverage. This commitment has culminated in the development of a UHC policy paper and a national roadmap. These documents serve as comprehensive guides, shaping the government's actions and strategies regarding universal health coverage.  The findings underscores how the NPA leverages its authority and convening power to drive inter-ministerial collaboration, shape policy definitions, enhance role clarity, and ensure financial feasibility, all aimed at achieving the goal of universal health coverage. The NPA's proactive role illustrates the significance of coordinated efforts in addressing complex societal challenges like healthcare access.  NPA coordination function was reportedly facilitated by its higher position in government bureaucracy favouring a focus on broader government issues, inclination towards consensus building and regular monitoring and evaluation activities as a basis for demand accountability from government MDAs. Government officials said as follows: “the mandates are described by law or policy, and they work like that. But as NPA, because we sit at the apex, we have seen that we need to break these silos because we are not seeing the desired results.—MDA-3 “ |
|  | In the context of government initiatives aimed at achieving universal health coverage (C), if planning authorities harness their authority and convening power to facilitate inter-ministerial collaboration (R2), then it fosters collaboration among government ministries, contributes to shaping policy definitions, clarifies roles, and ensures financial feasibility. | | | |
| Relevant IPT(s) | Confirm/Refute/ Refine* | Suggested Revisions* | | Notes* |
| IPT 5 | Confirm |  | |  |

| CMOC-37 | Context | Mechanism | Outcome | Excerpts from Text |
| --- | --- | --- | --- | --- |
|  | Existing legal institutional frameworks | R1: Inconsistent guidance  R2: Provision of inconsistent guidance by central coordinating agencies to various government sectors creates confusion among actors | Poor participation and involvement in coordinated action | The study identified a notable issue of inconsistent guidance stemming from central coordinating agencies. Specifically, it noted that entities like the Ministry of Finance, Planning, and Economic Development (MoFPED), the Office of the Prime Minister (OPM), and the National Planning Authority (NPA) were each providing separate guidance to various sectors within the government. Furthermore, the study highlighted a significant challenge in the form of limited capacity to enforce compliance with hierarchical structural instruments. One respondent pointed out that despite legal mandates, there were constraints in exerting control over other Ministries, Departments, and Agencies (MDAs) due to deficiencies in the legal and institutional frameworks. This inconsistency and limitation in enforcement mechanisms underscored the need for more streamlined and effective coordination within the government.  ‘The planning act provides that the Minister has the power to compel and penalise institutions that don’t provide rel- evant information for planning if requested by the NPA. So, I think those legal provisions compel participation in planning processes and alignment of budgeting processes to planning processes. However, there is a grey area on enforcement mechanisms and penalties’—MDA-14. |
|  | Existing legal institutional frameworks, (C) with legal provisions that compel participation in planning processes, (R1) encounter challenges when central coordinating agencies provide inconsistent guidance (R2) to various government sectors, resulting in confusion among actors resulting in poor coordination (O) | | | |
| Relevant IPT(s) | Confirm/Refute/ Refine* | Suggested Revisions* | | Notes* |
| IPT 5 | Confirm |  | |  |

| CMOC-38 | Context | Mechanism | Outcome | Excerpts from Text |
| --- | --- | --- | --- | --- |
|  | Interministerial institutional structures | R1: Limited decision-making powers of advisory boards  R2: Due to the limited power of advisory boards there is lack of binding of sectors | Suboptimal participation of actors outside the hosting sector | In Uganda, network-based instruments for coordination include various interministerial committees and advisory bodies that lack formal decision-making authority. These structures are designed to facilitate coordinated responses to specific policy matters like nutrition, universal health coverage (UHC), and early childhood development. Additionally, informal forums such as the Forum for the Permanent Secretaries and Commissioners exist. The technical working groups (TWGs) within sectors were seen as formal mechanisms to harness contributions from multiple sectors, emphasising the importance of coordinated efforts in policymaking and implementation.  But the Technical working groups are mandated to make sure that other sectors of relevance, including academia, are invited. They are part of the process and part of the decision-making process. —MOH-7  The TWGs reportedly offered opportunities for consultations, but respondents felt they could more do. Interviewees noted that these structures were further constrained by the suboptimal participation of actors outside the hosting sector(s) and a lack of binding authority.  Actually, some technical working groups require that we co-opt these other sector people. Some of them work more easily, for example, for adolescent health. But others don’t work. I said by design, they are programmed differently. Different activities are going on. —MOH-7 |
|  | Interministerial institutional structures, (C) characterised by limited decision-making powers (R1) of advisory boards, result in a lack of binding authority among sectors, (R2) ultimately leading to suboptimal participation of actors outside the hosting sector. (O) | | | |
| Relevant IPT(s) | Confirm/Refute/ Refine* | Suggested Revisions* | | Notes* |
| IPT 1 | Refine |  | |  |

| CMOC-39 | Context | Mechanism | Outcome | Excerpts from Text |
| --- | --- | --- | --- | --- |
|  | Hierarchical government structures | R1: Informal and volunteer committee meetings  R: The ratification requirement of higher bodies on the decisions made in informal volunteering meetings makes actors feel that their discussions/decisions are undervalued | Lack of interest in participation and meeting | Informal and voluntary committees face significant challenges in maintaining stakeholder interest and sustained collaboration. These challenges are rooted in concerns related to mandates and a limited shared vision among participants. An illustrative example provided by a Ministry of Health (MOH) official highlights this issue, specifically in the context of addressing Non-Communicable Diseases (NCDs). The official explained the formation of a multisectoral committee comprising key sectors such as Gender, Agriculture, Trade, Works, the Office of the Prime Minister (OPM), Finance, and the President's office, with the aim of preventing and controlling NCDs. Despite its inauguration in 2018, the committee has become inactive because other sectors do not perceive health-related matters as within their mandate. Consequently, these committees often assume advisory roles, and the decisions they make typically require ratification by higher hierarchical bodies. This example underscores the challenges faced by these committees in fostering sustained collaboration and the need for a more inclusive and shared vision among stakeholders.  First, we formed a multisectoral committee for the prevention and control of NCDs, and we met for some time. Of course, it involved all the key sectors, i.e. Gender, Agri- culture, Trade, Works, OPM, Finance, Presidents’ office. It was inaugurated in 2018. But the challenge is that it is inactive now because the other sectors don’t find the motivation. They don’t see it as their own mandate talking about health. —MOH-6 |
|  | In a hierarchical government structure (C ) if there is a requirement of ratification by higher bodies for decisions made by the smaller and informal gatherings, then actors perceive their discussions and decisions as undervalued (R) Consequently, this perception can lead to a lack of interest in participation and attendance at intersectoral meetings. (O) | | | |
| Relevant IPT(s) | Confirm/Refute/ Refine* | Suggested Revisions* | | Notes* |
| IPT 2 | Refine |  | |  |

| CMOC-40 | Context | Mechanism | Outcome | Excerpts from Text |
| --- | --- | --- | --- | --- |
|  | Resource-poor setting | R1: Lack of resources   R2: Due to unavailability of appropriate resources actors feel constrained to perform the day-to-day tasks leading to delay in decision making | Resulting in poor engagement | Sectoral Working Groups (SWGs) are portrayed as essential formal mechanisms for collective decision-making at the sectoral level, in alignment with the government's Sector Wide Approach (SWAP) policy, emphasising coordination within and among SWGs. However, evidence from documents and interviews reveals a gap between the ideal concept and the actual functionality of these groups. A non-state actor pointed out that while sectors have been organised into SWGs, not all of them are fully operational. Some lack functioning secretariats, budget allocations, active steering mechanisms, and regular engagement. These challenges parallel those faced by Technical Working Groups (TWGs), as mentioned by several government officials, particularly the issue of inadequate budget allocation for multisectoral initiatives. The study illustrates the reliance on goodwill and persuasive efforts due to the absence of dedicated financing mechanisms for many multisectoral activities. For instance, while the Ministry of Water allocates a substantial budget for sanitation, the Ministry of Health does not, showcasing disparities in resource allocation among sectors. This underscores the need for addressing these constraints to enhance the effectiveness of both SWGs and TWGs in promoting multisectoral collaboration.  “Sectors have been constituted into sector working groups, but these sectors are not all working. For example, some do not have functioning secretariats, others do not have budgets, others do not have active steering mechanisms, and others do not have regular engagements—NSA-1.  ....if an activity is due, then there (should be) an arrangement that ensures that it is financed because most of the multisectoral arrangements depend on courtesy, on pleadings—MDA-1  ...(regarding sanitation), you find the Ministry of Water has dedicated for several years two billion on that, (but) the Ministry of Health nothing—MDA-4 |
|  | In a resource-poor setting, (C ) unavailability of appropriate resources (R1) can constrain actors in performing their day-to-day tasks, (R2) ultimately leading to delays in decision-making and resulting in poor engagement (O). | | | |
| Relevant IPT(s) | Confirm/Refute/ Refine* | Suggested Revisions* | | Notes* |
| IPT 3 | Confirm |  | |  |

| CMOC-41 | Context | Mechanism | Outcome | Excerpts from Text |
| --- | --- | --- | --- | --- |
|  | Hierarchical power structures within govt systems | R: Due to unequal distribution of power OR giving more power to one department over others makes other sectors weaker in terms of programmatic decision making due to poor allocation of financial resources | Lack of programme implementation | study in Uganda has unveiled a complex interplay of hierarchical and network mechanisms within the national government's Coordination Instruments (CIs). Interestingly, many of these instruments exhibit characteristics of both mechanisms. For instance, negotiation and consultative bodies like interministerial committees, primarily grounded in a network-based logic, also possess hierarchical attributes that allow them to tap into the structural power associated with hierarchy. Notably, central coordination agencies, such as the Ministry of Finance with its authority over line ministries through budget approvals, and the Prime Minister's office, which relies on its hierarchical power for coordination, exemplify how coordination is achieved through structural power derived from legal mandates. This intricate blend of hierarchical and network mechanisms underscores the multifaceted nature of governance coordination at the national level in Uganda. |
|  | Hierarchical power structures in government systems, (C) characterised by an unequal distribution of authority favouring certain departments, (R1) can weaken the programmatic decision-making capabilities of other sectors. This power imbalance often leads to inadequate allocation of financial resources, resulting in a lack of effective program implementation. (O) | | | |
| Relevant IPT(s) | Confirm/Refute/ Refine* | Suggested Revisions* | | Notes* |
| IPT 5 | Confirm |  | |  |

| CMOC-42 | Context | Mechanism | Outcome | Excerpts from Text |
| --- | --- | --- | --- | --- |
|  | Multisectoral policy -complex structure of coordination bodies | R1: Influential individual holding multiple positions  R2: hinder transparent communication among actors, actors do not open up due to power exerted by the influential individual | (O) Creating competing agendas, influence resource allocation decisions, foster fragmentation, and diminish collaboration efforts within coordination bodies. | Coordination bodies within a government are themselves proactive entities that aim to optimise organisational or collective objectives. They take intentional actions to enhance their coordination functions. In the context of Uganda, the Office of the Prime Minister (OPM) underwent internal restructuring and developed pertinent policy tools, such as the National Coordination Policy. A closer examination of these coordination bodies reveals that they are not uniform but rather complex entities with multiple roles and identities, which can add layers of complexity to their functioning. For instance, the First Lady, who champions adolescent health, also leads the Ministry of Education, which plays a significant role in adolescent health matters. Moreover, the OPM itself comprises special ministries and various departments. This compartmentalisation introduces power differentials and reinforces internal silos, which can ultimately undermine the effective functioning of coordination bodies by creating divisions and hindering collaboration. |
|  | In a complex multisectoral policy context, (C) when an influential individual simultaneously occupies multiple positions (R1) within the committee, transparent communication is impeded, (R2) resulting in unequal resource allocation and suboptimal collaboration. (O) | | | |
| Relevant IPT(s) | Confirm/Refute/ Refine* | Suggested Revisions* | | Notes* |
| IPT 5 | Confirm |  | |  |

| Author | Year | Publication Type | Aims/Objectives | Setting |
| --- | --- | --- | --- | --- |
| Anne L | 2018 | Peer reviewed article | Challenges faced by Peru’s multi sectoral policy | Peru |
| Study Participants | Study Design | Findings |  |  |
| Govt stakeholders, NGO and academia | Qual | The findings highlight three key challenges faced by the group in its multisectoral policy context: 1) the selection of representatives, 2) achieving a balance in membership and leadership across sectors, and 3) managing role transitions and conflicts. In response to these challenges, the group has implemented several measures, including a rotation system for formal leadership roles, professionalisation of management functions, the introduction of electoral systems for civil society representation, and the development of conflict-of-interest guidelines. These experiences provide valuable lessons for other countries grappling with the configuration of multisectoral groups. Additionally, they offer insights for donors who mandate the establishment of such groups, emphasising the need to combine inclusive participation with practical adaptability and a realistic approach. | | |
| Key messages of the paper | | Contributor |  | |
| The article highlights the challenges faced by multi-sector groups like CONAMUSA in achieving broader participation and presents organizational strategies employed to overcome these challenges. It serves as a valuable resource for countries and donors seeking to establish similar groups while emphasizing the importance of balancing idealism with practical considerations. | | Secondary | None | |

| CMOC-43 | Context | Mechanism | Outcome | Excerpts from Text |
| --- | --- | --- | --- | --- |
|  | Collaborative arrangements in National health policy | R: Platform for collaboration of different sectors  R2: Lack of diversity in representation of sectors. Smaller organisations feel overshadowed by the larger and more influential entities | This dominance creates a power imbalance that can hinder effective collaboration and decision-making within collaborative arrangements | The selection processes for government representatives in CONAMUSA (National AIDS, Tuberculosis, and Malaria Council) are well-established through elections and appointments. However, there are no equivalent mechanisms for civil society representatives. The selection methods for civil society members vary, including delegation for government members, elections for NGOs and representatives of populations affected by HIV, TB, and malaria, nomination by assembly for representatives of vulnerable populations, and self-expression of interest for academic and religious institutions. Some smaller organisations have raised concerns about the dominance of more established NGOs and civil society associations within CONAMUSA, calling for more diverse representation. To address this, associations of people living with HIV/AIDS (PLWHA) introduced a democratic voting process, establishing an electoral committee to oversee a national secret ballot election with the participation of 66 accredited PLWHA associations. |
|  | In an existing national collaborative health policy arrangement (C), if there is a lack of diversity in the selection of sectors when forming an intersectoral body (R1), smaller organisations feel overshadowed by the larger and more influential entities, leading to hindrances in collaborative decision-making processes (O1) and hampering intersectoral program implementation (O2). | | | |
| Relevant IPT(s) | Confirm/Refute/ Refine* | Suggested Revisions* | | Notes* |
| IPT 2 | Confirm |  | |  |

| CMOC-44 | Context | Mechanism | Outcome | Excerpts from Text |
| --- | --- | --- | --- | --- |
|  | Collaborative policies structures | R1: Vice Chair, revising membership eligibility, and establishing electoral systems  R2: This gives a fair chance to other sectors for selection that brings a sense of equality among all the sectors that addresses power imbalances | Active participation in collaborative arrangement | he case of CONAMUSA illustrates that expanding participation in decision-making processes and healthcare can enhance access for stigmatised populations. However, achieving representative and balanced multi-sector participation can be complex and impose significant time and role conflicts on civil society members. The case offers two crucial lessons for groups seeking to broaden participation: the significance of institutionalising participation mechanisms and health policy reforms, and the influence of civil society mobilisation.  To balance power, CONAMUSA restructured its organisation, introduced a Vice Chair, revised membership eligibility to prevent conflicts of interest, and established electoral systems for civil society. Institutionalising these mechanisms ensures inclusive participation persists beyond specific individuals' involvement. Creating multi-year strategic plans and passing legislation further solidifies CONAMUSA's impact in national health policy.  The experience of CONAMUSA underscores the importance of mobilisation in facilitating civil society involvement. Well-organised groups, such as PLWHA and LGBT movements, have influenced CONAMUSA's structure and agenda by advocating for specific policy changes, increased leadership opportunities, and better representation.  Donor-mandated participation can elevate marginalised voices and promote cross-sector interaction, as demonstrated by CONAMUSA's case. However, this model's applicability should consider the level of civil society engagement in different regions. Selection of civil society representatives should align with the group's mandate, such as including populations affected by specific diseases or focusing on geographic diversity. Additionally, powerful actors like the government may require strong external incentives for engagement.  Sustaining CONAMUSA beyond external funding remains a significant challenge, given its reliance on external incentives. |
|  | If the existing Collaborative policies (C ) offer a fair opportunity for sectors across the board to be selected,(R1) fostering a sense of equality among all participants (R2) and promoting active engagement in collaborative arrangement (O ) | | | |
| Relevant IPT(s) | Confirm/Refute/ Refine* | Suggested Revisions* | | Notes* |
| IPT 3 | Confirm |  | |  |

| Author | Year | Publication Type | Aims/Objectives | Setting |
| --- | --- | --- | --- | --- |
| Sunny S. Kim | 2017 | PR | how intersectoral convergence in nutrition programming is operationalised between ICDS and NRHM from the state to village levels in Odisha, and the factors influencing convergence in policy implementation and service delivery. | India |
| Study Participants | Study Design | Findings |  |  |
| Health and ICDS staff | Qual | Close collaboration at state level was observed in developing guidelines, planning, and reviewing programs, facilitated by a shared motivation and recognised leadership for coordination. However, the health department was perceived to drive the agenda, and different priorities and little data sharing presented challenges. At the district level, there were joint planning and review meetings, trainings, and data sharing, but poor participation in the intersectoral meetings and limited supervision. While the block level is the hub for planning and supervision, cooperation is limited by the lack of guidelines for coordination, heavy workload, inadequate resources, and poor communication. Strong collaboration among FLWs was facilitated by close interpersonal communication and mutual understanding of roles and responsibilities. | | |
| Key messages of the paper | | Contributor |  | |
| The research highlights the challenges and opportunities for sectoral convergence in maternal and child health and nutrition interventions in the Indian context. It underscores the importance of effective coordination mechanisms and shared priorities to improve service delivery and outcomes. | | Secondary | None | |

| CMOC-45 | Context | Mechanism | Outcome | Excerpts from Text |
| --- | --- | --- | --- | --- |
|  | State intersectoral health care policies | R1: Intersectoral meetings  R2: Due to the narrow focus of one department and essential interventions by one dept gives a feeling of health department being superior to others | Leading to biased decision making and poor engagement of other sectors | Findings highlight that although there is a shared understanding of goals and priorities between the health and nutrition sectors, the health department tends to dominate in practice. This is partly because some essential interventions, such as immunisation, are delivered by the health department. The narrow focus on specific health indicators during coordination meetings, driven by the health department's priorities, can hinder meaningful improvements in coordination for nutrition interventions. Additionally, frontline worker coordination is better for services primarily driven by the health department, leading to uneven power dynamics and coordination challenges for services like counselling for infant and young child feeding (IYCF). The passage suggests that power dynamics and sectoral priorities can impact the effectiveness of intersectoral coordination in healthcare. |
|  | "When state intersectoral healthcare policies lack balance (C) and primarily prioritise one department's objectives and essential interventions, they foster a perception of superiority within the health department, (R) ultimately leading to diminished engagement from other sectors. (O) | | | |
| Relevant IPT(s) | Confirm/Refute/ Refine* | Suggested Revisions* | | Notes* |
| IPT 4 | Refute |  | |  |

| Author | Year | Publication Type | Aims/Objectives | Setting |
| --- | --- | --- | --- | --- |
| Santosh R. Pathak | 2022 | PR | The aim of the study is to examine and promote a multi-stakeholder partnership approach, complemented by technological solutions developed by ICIMOD (International Centre for Integrated Mountain Development), to address the significant challenges and explore opportunities in river basin management in Afghanistan. | Afghanistan |
| Study Participants | Study Design | Findings |  |  |
|  | Qual | The major finding of the study is that adopting a multi-stakeholder partnership approach, along with the use of technological solutions developed by ICIMOD, can effectively address the substantial challenges and opportunities related to river basin management in Afghanistan. This approach facilitates collaboration among various stakeholders and enhances water resource management in the region. | | |
| Key messages of the paper | | Contributor |  | |
| This study delves into the complexities of water governance and emphasizes the need for a multi-faceted approach. Water governance is depicted as intricate and contentious, requiring the active involvement of numerous stakeholders and the strategic application of technology to efficiently manage water resources. Afghanistan's recent water law is spotlighted for its strong focus on engaging stakeholders at various administrative levels, ensuring fair water allocation, and facilitating collaborative decision-making, from sub-basins to the national stage. | | Primary | None | |

| CMOC-46 | Context | Mechanism | Outcome | Excerpts from Text |
| --- | --- | --- | --- | --- |
|  | Governance and bureaucratic structures | R1: Multiple government agencies  R2: Each agency having different interests and distinct responsibilities creates ambiguity in terms of having formal control over the decision-making process leading to potential imbalance in decision-making | Difference in understanding and poor engagement | “At the initial discussion with these stakeholders, SWaRMA faced challenges of control and communication among the stakeholders, as multiple government agencies were in- volved. Stakeholders were communicating without a clear communication channel and plan due to which some of the stakeholders felt left out or not informed. All the stake- holders were not on same page in terms having common understanding of the project. This was mainly due to lack of clarity regarding the roles of the respective agencies, which resulted in confusion about the partnership’s purpose, its objectives, and how to measure its success”  The power dynamics affecting collaboration in the SWaRMA partnership become quite evident in following ways  Control and Decision-Making: Multiple government agencies were involved in the partnership, which inherently introduced different power dynamics. Each agency likely had its own priorities, interests, and ways of making decisions. This created challenges in terms of who had control over the partnership, how decisions were made, and whose interests were prioritised. Some agencies may have had more influence or authority than others, leading to potential imbalances in decision-making power.  Differing Understandings: The absence of a common understanding of the project among stakeholders highlights another dimension of power dynamics. When there is a lack of clarity about roles, objectives, and success criteria, some stakeholders may exploit this ambiguity to advance their interests. Those with more power or resources may assert their interpretations and priorities, potentially sidelining the perspectives of others. |
|  | When national governance and bureaucratic structures involve multiple government agencies (C ) with each agency's varying interests and distinct responsibilities create ambiguity in terms of formal control over the decision-making process, (R) potentially resulting in an imbalance in decision-making, differences in understanding, and poor engagement ( O) | | | |
| Relevant IPT(s) | Confirm/Refute/ Refine* | Suggested Revisions* | | Notes* |
| IPT 3 | Refine |  | |  |

| CMOC-47 | Context | Mechanism | Outcome | Excerpts from Text |
| --- | --- | --- | --- | --- |
|  | Existing communication structures within Intersectoral Partnership policies | Due to the absence of absence of clear communication channels and a coordinated communication plan, stakeholders do not effectively share information, ideas, or plans with one another. As a result of the communication challenge, some actors feel left out | Lack of meaningful engagement in decision making process leading to poor ISC | Communication Gaps: The lack of a clear communication channel and plan further exacerbated power dynamics. When stakeholders, especially government agencies, communicate without an established structure, those with more influence or resources may dominate the conversation. This can lead to some stakeholders feeling left out or not adequately informed about important decisions and developments. Effective communication is crucial for equitable collaboration.  Purpose and Objectives: The confusion about the partnership's purpose, objectives, and how to measure its success is a reflection of power dynamics related to goal setting. Stakeholders with more influence may shape the partnership's goals to align with their own priorities, potentially neglecting the broader interests or needs of other stakeholders. |
|  | When Intersectoral Partnership policies suffer from the absence of clear communication channels and a coordinated communication plan, (C) that will hinders effective sharing of information, ideas, or plans among stakeholders.(R ) This results in lack of meaningful engagement in the decision-making process ultimately leads to poor Intersectoral Collaboration." (O ) | | | |
| Relevant IPT(s) | Confirm/Refute/ Refine* | Suggested Revisions* | | Notes* |
| IPT 4 | Confirm |  | |  |
| CMOC-48 | Context | Mechanism | Outcome | Excerpts from Text |
|  | Intersectoral Partnership policies | Lacking clarity in the purpose leads to different interpretation from different partners leading to confusion among actors regarding contributions to the partnership and failure in comprehending how their actions fit into the broader goals | (O1) Uncertainty in objective measurement and confusion about partnership’s purpose (O2) | Among the stakeholders were those who were contributors to the project and those who had little to contribute but stood to benefit. The partnership approach created an enabling environment in which to carry out the stakeholder consultations where the challenges could be elicited and discussed in a transparent and amicable manner. From the inception, SWaRMA focused on these partnership challenges to clarify and understand the roles and responsibilities of each stakeholder by identifying the value addition that each stakeholder would bring to the project and the individual interest each stakeholder could reap from being part of the project.  The clarity attained regarding the roles, contributions, and benefits of stakeholders helped to ease the tension caused by ambiguity of stakeholder roles and their individual interests. This approach also facilitated the mutual development of strategies to implement the project for the purpose of improving the status of water resource management in Afghanistan. The clarity on roles and the transparency maintained by the partners also created a strong foundation for SWaRMA to reach a successful conclusion.  “This partnership helped to bring all the partners together but stand out point was that due to this partnership we could have a bigger picture of WRM in Afghanistan together, which really empowered everyone through knowledge sharing and capacity building”, NWARA, KU & NEPA.  The partnership facing difficulty due to   Lack of Common Understanding: Another challenge was the absence of a common understanding of the project among all stakeholders. This means that different stakeholders had varying interpretations of the partnership's purpose, objectives, and criteria for measuring success. This lack of alignment in understanding can lead to confusion and disagreements within the partnership. Role Ambiguity: The root cause of these challenges was identified as a lack of clarity regarding the roles and responsibilities of the respective government agencies. When roles are unclear, stakeholders may struggle to define their contributions to the partnership and may not fully comprehend how their actions fit into the broader goals. Confusion About Partnership's Purpose: The culmination of these challenges resulted in confusion about the overall purpose of the partnership. When stakeholders have different interpretations of the partnership's objectives, it becomes difficult to establish a unified direction and work towards shared goals. Objective Measurement Uncertainty: In addition to the partnership's purpose, there was also uncertainty about how to measure its success. This ambiguity regarding success criteria further complicated the partnership's early stages. |
|  | If intersectoral partnership policies lack clarity in the purpose of the partnership (C), this can lead to different interpretations from various partners, resulting in confusion among actors regarding their contributions to the partnership and their failure to comprehend how their actions fit into the broader goals (R). This uncertainty in objective measurement and confusion about the partnership's purpose (O2) ultimately hampers its effectiveness. | | | |
| Relevant IPT(s) | Confirm/Refute/ Refine* | Suggested Revisions* | | Notes* |
| IPT 5 | Confirm |  | |  |

| CMOC-49 | Context | Mechanism | Outcome | Excerpts from Text |
| --- | --- | --- | --- | --- |
|  | Existing governance structures | R1: Formation Committee/a common platform  R2: Creating a committee/a common platform demonstrates a deliberate effort to distribute decision-making authority making stakeholders feel included and given a fair chance and a democratic way to ensure that all voices are heard. | O1: Power to influence the partnership's direction is shared more evenly  O2: Greater degree of cooperation | 4.2. Formalising Partnership  Power dynamics in this context are evident in several aspects:  Decision-Making Authority: The formation of a governance structure, including a Steering Committee and Technical Coordination Committee, reflects power dynamics. The steering committee oversees the progress of different partners, indicating a level of authority and control over the partnership's direction. This can influence decision-making and the allocation of resources.  Selection of Focal Ministry: The partners agreed to name the Ministry of Energy and Water (MEW) as the focal institution. This decision involves power dynamics as it designates a particular government agency as a key player, potentially giving it more influence in decision-making processes within the partnership.  Participatory Mechanism: The participatory mechanism used to form the governance structure suggests an attempt to distribute power more evenly. However, the level of influence of each partner within these structures may still vary based on factors such as resources and expertise.  Capacity Building: The focus on capacity building can also impact power dynamics. Partners who receive training and capacity building may become more influential due to their enhanced skills and knowledge. |
|  | If governance structures, (C ) provide a common platform, (R1 ) and exemplify a deliberate endeavour to equitably distribute decision-making authority, then it fosters a sense of inclusion among stakeholders and provides them with a fair and democratic avenue to ensure that all voices are heard, (R2) this results in a more balanced distribution of power to influence the partnership's direction (O1) and fostering a greater degree of cooperation. (O2)" | | | |
| Relevant IPT(s) | Confirm/Refute/ Refine* | Suggested Revisions* | | Notes* |
| IPT 3 | Refine |  | |  |

| CMOC-50 | Context | Mechanism | Outcome | Excerpts from Text |
| --- | --- | --- | --- | --- |
|  | Inclusive governance and bureaucrat structures | R1: Frequent communication and remote participation  R2: Creates a sense of equal participation and fosters open communication that brings more clarity in roles and responsibilities among each sector | 01: Increases stakeholder’s confidence in the partnership's governance structure.  O2: Greater degree of collaboration | 4.2. Formalising Partnership  Power dynamics in this context are evident in several aspects:  The frequency of meetings, such as the choice to convene Steering Committee meetings three times during the program and Technical Coordination Committee meetings quarterly, can significantly impact participation and influence within the partnership. Stakeholders who have the capacity to attend meetings more frequently may wield greater input and exert more influence on decision-making processes. Additionally, the allowance of remote participation by ICIMOD and CSIRO underscores power dynamics associated with resource access, potentially providing advantages to those with the necessary resources and technology to engage remotely, thereby influencing discussions and decisions. Furthermore, the clarity attained through the formalisation of roles and responsibilities also serves as a factor in power dynamics, ensuring that each partner comprehends their role and potential spheres of influence within the partnership."  “Timely updates, communications, a consultative decision-making approach were some of the key features of this partnership.”—Kabul Polytechnic University. |
|  | When governance and bureaucratic structures (C) prioritise inclusivity,(R1 ) followed by frequent communication and remote participation (R1) then it instils a sense of equal participation and encourages open communication, ultimately enhancing clarity regarding roles and responsibilities across various sectors (R2). This leads to increased stakeholder confidence in the partnership's governance structure (O1) and fosters a greater degree of collaboration (O2)." | | | |
| Relevant IPT(s) | Confirm/Refute/ Refine* | Suggested Revisions* | | Notes* |
| IPT 3 | Refine |  | |  |

| CMOC-51 | Context | Mechanism | Outcome | Excerpts from Text |
| --- | --- | --- | --- | --- |
|  | Existing governance structures | R1: Capacity building and training  R2: Leads to clarity in tasks and comprehensive understanding of the programme that not only empowers the stakeholder but brings a sense of ownership among stakeholders | O1: An inclusive and collaborative approach to decision-making  O2: Long-term organisation development and sustainability of partnerships | 4.3 Partnership Execution and Management  In the execution and management of the SWaRMA partnership, a focus on capacity enhancement, clear communication, and collaborative decision-making played a pivotal role. The partnership prioritised building the capacities of individuals and institutions, aiming to contribute to long-term organisational development. Capacity enhancement events were conducted, ensuring full participation and involvement of stakeholders. The governance structure involved a Steering Committee for strategic decisions, a Technical Coordination Committee for program-level decisions, and focal persons from partner organisations for day-to-day operational matters.  Despite challenges posed by remote project sites and the inability to hold face-to-face meetings, partners committed to results-based monitoring and reporting. This approach fostered a sense of ownership among partners and increased the likelihood of sustainability beyond the project phase. The collaborative design and implementation of solutions through the partnership approach facilitated engagement among stakeholders and contributed to a comprehensive understanding of Water Resource Management (WRM) in Afghanistan, empowering all involved through knowledge sharing and capacity building. |
|  | When governance structures and intersectoral policies (C ) involve capacity building and training component (R1), then it this leads to clarity in tasks, a comprehensive understanding of the programme and stakeholders feeling empowered to execute the tasks (R2), resulting in an inclusive and collaborative approach to decision-making (O2) and contributing to long-term organisational development and sustainability of partnerships. | | | |
| Relevant IPT(s) | Confirm/Refute/ Refine* | Suggested Revisions* | | Notes* |
| IPT 5 | Confirm |  | |  |

| CMOC-52 | Context | Mechanism | Outcome | Excerpts from Text |
| --- | --- | --- | --- | --- |
|  | Communication structures | R1: Integration of reviews into the project cycle  R2: The review process involving facilitation discussions creates an environment of open communication where actors feel their views are valued challenges heard which increases shared understanding | O1: Resolves conflict among stakeholders  O2: Increases accountability | SWaRMA employed a partnership approach that included regular health checks and reviews to assess the well-being of the partnership. This collective process focused on various components, such as strategies, governance structure, communication, challenges, and opportunities. It aimed to continually improve the partnership for sustainable impact. The reviews were integrated into the project cycle to ensure effectiveness and efficiency. They highlighted successes, areas for improvement, unforeseen benefits, and potential partnership modalities for the future.  The review process involved facilitated discussions where partners reached common understandings on issues. Facilitators engaged with stakeholders individually to understand their concerns, challenges, and achievements. Key concerns and achievements were then discussed collectively in annual workshops facilitated by a partnership broker. These workshops helped evaluate the partnership's health and identify necessary remedies.  Partners recognised the significance of this partnership approach in facilitating effective engagement, cooperation, and successful implementation of activities. It provided a strong platform for collaboration among stakeholders within Afghanistan and at the regional level. This not only led to successful outcomes during the project but also fostered interest in continuing the work beyond its duration.  The collective review highlighted several successful outcomes of the partnership, including the establishment of Afghanistan's first benchmark glacier, widespread adoption of J2000 hydrological modeling, the creation of a science-based multi-stakeholder partnership at the national and regional levels, enhanced knowledge in Multi-Scale Integrated River Basin Management, advancements in water information systems, the adoption of technology for flood monitoring, and opportunities for gender integration in water resource management.  Tracer surveys and capacity self-assessments showed that participants gained confidence and knowledge through project activities. These reviews improved accountability, efficiency, and the overall value of the SWaRMA initiative. Partners expressed positive sentiments about the partnership's growth, energy, and collaborative decision-making approach, emphasising its success.  “It has been a good growing partnership, which has evolved with a lot of positive energy and all the partners in table providing their best of the effort. Timely updates, communications, consultative decision-making approach were some of key features of this partnership”—MEW. |
|  | When existing communication structures (C ) include review mechanisms (R1) including facilitation discussions, creates an environment of open communication where all actors feel their views are valued, challenges are heard, (R2) and this contributes to resolving conflicts among stakeholders (O1) and increasing accountability." (O2) | | | |
| Relevant IPT(s) | Confirm/Refute/ Refine* | Suggested Revisions* | | Notes* |
| IPT 5 | Refine |  | |  |

| Author | Year | Publication Type | Aims/Objectives | Setting |
| --- | --- | --- | --- | --- |
| Enyi Etiaba | 2022 | Peer reviewed Article | Examine collaboration in the implementation of maternal, neonatal, and child health (MNCH) programs within Nigeria's federal government system, with a focus on understanding the dynamics of intergovernmental collaboration. The study seeks to identify principles that can be applied to other multilevel governance contexts, particularly in low-income countries. |  |
| Study Participants | Study Design | Findings |  |  |
|  |  | The paper emphasizes the need for a comprehensive approach to intersectoral collaborations following the HiAP framework, with a particular focus on governance, trust-building, and the importance of both internal and external legitimacy and credibility in achieving better population health and reducing health inequalities. It offers valuable insights for policymakers and practitioners working in the field of public health and policy. | | |
| Key messages of the paper | | Contributor |  | |
| The study highlights that structural misalignments and ineffective collaboration dynamics in Nigeria's federal government system hinder the successful implementation of MNCH programs. These findings have implications for improving intergovernmental collaboration in healthcare initiatives, particularly in similar multilevel governance contexts, including low-income countries. | | Primary | Power dynamics, both within and between government levels, influenced the effectiveness of collaboration in implementing MNCH programs. Asymmetries in power, fiscal centralization, and policy design contributed to the study's findings of misaligned governance structures and challenges in achieving collaborative actions. | |

| CMOC-53 | Context | Mechanism | Outcome | Excerpts from Text |
| --- | --- | --- | --- | --- |
|  | Existing governance and administration structures | R1: Global Push of IMNCH strategy  R2: Due to the global push to improve MNCH, stakeholders felt the need for engagement for shared vision | Joint guidelines and collaborated working | Key international contexts included the Millennium Development Goals (MDGs), with a particular emphasis on Nigeria and other high Maternal, Neonatal, and Child Health (MNCH) burden countries striving to achieve MDGs 4 and 5. International economic drivers included debt relief in 2005 and financial support from the World Bank for the Saving One Million Lives (SOML) Performance for Results (PfR) program in 2015.  At the national level, the study considered the influence of governance, political, and economic arrangements within Nigeria. Administrative decentralization granted states autonomy over national-level policies, while political processes, including elections at both national and subnational levels, played a role in shaping the political landscape. Economically, fiscal resources, primarily from oil revenues, were centralized. Specific program-related factors also influenced the MNCH policy process, and the study's findings regarding intergovernmental collaboration dynamics, collaborative actions, and outcomes were detailed in Table 2. |
|  | When existing governance and administration structures (C) were influenced by the global push of the Integrated Maternal, Neonatal, and Child Health (IMNCH) strategy,( R1) as stakeholders recognized the importance of engagement(R2) to foster a shared vision, joint guidelines, and collaborative working. | | | |
| Relevant IPT(s) | Confirm/Refute/ Refine* | Suggested Revisions* | | Notes* |
| IPT 5 | Confirm |  | |  |

| CMOC-54 | Context | Mechanism | Outcome | Excerpts from Text |
| --- | --- | --- | --- | --- |
|  | Governance and administration structures | Due to dominance of national in key aspects of programme key stakeholder felt undervalued | Leading to poor engagement | The study observed that early stakeholder engagement and consultation played a crucial role in the successful adoption of the framework strategy at subnational levels for maternal, neonatal, and child health (MNCH). Subnational stakeholders were actively involved in the development of guidelines and plans, creating a sense of ownership and commitment to the program. However, this high level of engagement was not sustained when the policy was decentralized to subnational levels. States were expected to take ownership and roll out the program in their respective regions, leading to variations in the timing of implementation. In subsequent programs, such as the MSS and SURE-P MCH, the design process lacked the same level of consultative engagement, which created a disconnect between stakeholders and affected the execution of these programs. This shift in approach highlighted a diminishing collaborative intent in the later stages of the initiatives. |
|  | When governance and administrative structures (C) dominate the key aspect of the programmes or policies, stakeholder feel undervalued and unheard-of subnational level challenges (R), such top down approach leads to poor engagement of sectors (O) | | | |
| Relevant IPT(s) | Confirm/Refute/ Refine* | Suggested Revisions* | | Notes* |
| IPT 5 | Confirm |  | |  |

| CMOC-55 | Context | Mechanism | Outcome | Excerpts from Text |
| --- | --- | --- | --- | --- |
|  | Concentration of power in the hands of national authorities | Due to top decision-making process resources were diversified leading to poor attention to the primary programme needs leading to sense of disempowerment among other stakeholders | Limiting the engagement | The implementation of the Saving One Million Lives Performance for Results (SOML PfR) program faced challenges stemming from subnational governance structures. These structures granted executive governors’ significant powers over all sectors, including control over program funds. Accountability mechanisms for these funds were inadequate, and even though program funds were ring-fenced, they still required the governor's approval for expenditure. In some cases, governors redirected program funds to other health sector projects, leading to mis procurements of items like tricycle ambulances and generators. These purchases were not aligned with SOML PfR objectives, resulting in a diversion of resources away from critical maternal and child health indicators. This indicated a lack of priority attention to maternal and child health within the program implementation. |
|  | If there is a concentration of power in the hands of national authorities, (C) a lack of financial accountability, and a diversion of resources away from the programme goals, then the principles of collaboration will be undermined. This can lead to a sense of disempowerment among other stakeholders, (R) reduced engagement, and a breakdown in the collaborative process, ultimately resulting in poor collaboration in implementing the programme. (O) | | | |
| Relevant IPT(s) | Confirm/Refute/ Refine* | Suggested Revisions* | | Notes* |
| IPT 5 | Confirm |  | |  |

| CMOC-56 | Context | Mechanism | Outcome | Excerpts from Text |
| --- | --- | --- | --- | --- |
|  | National health policy context | R1: Leadership transition  R2: Introduction of new actors such as ministers, governors, and commissioners, then few stakeholders may not embrace new ideas due to perception that they are not aligned with their mandates leading to lack of accountability | Leading to poor engagement | Leadership transitions that occur during election cycles involve the emergence of new political leaders, typically governors and commissioners, who hold significant decision-making authority within their regions or sectors. This shift in power dynamics can have a notable impact on collaborative initiatives. The prioritization of collaborative efforts hinges on the new leaders' political agendas, priorities, and vested interests, which may either bolster or diminish ongoing collaborative projects. Moreover, the advocacy and momentum behind collaborative programs are strongly affected by these leaders' decisions and actions, including their capacity to allocate resources and champion initiatives. Additionally, the perception of whether collaborative programs align with a state's mandate is molded by these power dynamics; new leaders might not fully embrace initiatives if they do not align with their political interests, potentially jeopardizing the overall success of collaborative endeavors. In essence, the dynamics of power and leadership changes during election cycles significantly influence the course and outcomes of collaborative efforts. |
|  | Within national health policy context, (C ) if there is no prioritisation of collaborative working structures and if there is a change in the leadership, then it can significantly influence the direction, prioritisation, and success of collaborative efforts. (R) The unique perspectives and priorities that new leaders bring can either support or hinder the continuity and effectiveness of ongoing programs. | | | |
| Relevant IPT(s) | Confirm/Refute/ Refine* | Suggested Revisions* | | Notes* |
| IPT 5 | Confirm |  | |  |

| CMOC-57 | Context | Mechanism | Outcome | Excerpts from Text |
| --- | --- | --- | --- | --- |
|  | Hierarchical governance and administrative structures | Division of power and responsibilities between federal, state and local level leads to concerns among stakeholders related to authority, local priorities, political implications, resource availability, perceptions of policy implementation, alignment with local needs. These factors collectively contribute to the mechanism of subnational executive powers and a lack of political will, hindering the collective action at the local level. | Poor engagement | Despite strong overarching policies, substantial resources, and national leadership, the collaborative goals set for MNCH policy implementation were not fully realized. Early collaboration efforts were witnessed during the initial stages of the strategy development, but they failed to sustain over time. Several factors contributed to this, including misaligned governance structures, fiscal and administrative capacity issues, and the reluctance of subnational entities to collaborate. These power imbalances and governance arrangements, coupled with the top-down institutional design, presented significant challenges to intersectoral collaboration.  The division of power and responsibilities between the federal, state, and local government levels in Nigeria played a critical role. Subnational executive powers and a lack of political will at the state level hindered the successful translation of national initiatives and policies to the subnational level. Inadequate fiscal and administrative capacity, as well as issues related to collaboration, further complicated the situation. While other countries like India and Kenya have attempted to address similar issues through devolution and shared decision-making, Nigeria's challenges were primarily linked to political will and the perceived top-down nature of healthcare policies.  The study ultimately suggests the need for full devolution of healthcare responsibilities to subnational governments in Nigeria, with the national level providing independent evaluation and guidance. This would bring healthcare policy implementation closer to the communities affected, improve subnational commitment, and promote effective collaboration. However, this recommendation also has broader political and economic implications and requires sustained advocacy efforts for MNCH and innovative approaches for achieving distributed leadership across government levels, especially in resource-limited low- and middle-income countries. |
|  | Hierarchical governance and administrative structures (C) results in the division of power and responsibilities between federal, state, and local levels, then stakeholders negatively affected by authority, local priorities, political implications, resource availability, perceptions of policy implementation, and alignment with local needs. These concerns collectively contribute to hindering collective action at the local level. | | | |
| Relevant IPT(s) | Confirm/Refute/ Refine* | Suggested Revisions* | | Notes* |
| IPT 5 | Confirm |  | |  |

| Author | Year | Publication Type | Aims/Objectives | Setting |
| --- | --- | --- | --- | --- |
| Marysol Astrea Balane | 2020 | Peer reviewed article | The aim of this study is to address the gap in understanding how complex concepts such as power, interest, and position are operationalized and assessed in stakeholder analyses related to policy. The study aims to develop a framework that can be applied to policy implementation in low- and middle-income countries |  |
| Study Participants | Study Design | Findings |  |  |
|  |  | The developed framework provides a practical tool for the analysis of policy actors' characteristics and addresses the complexities involved in assessing complex concepts within stakeholder analyses. This framework contributes to health policy research by offering a useful approach to understanding and analyzing stakeholders in policy implementation. | | |
| Key messages of the paper | | Contributor |  | |
| The study recognized that the intersection of characteristics of actors, such as their level of knowledge and interest, plays a crucial role in determining their positions on a policy. This emphasizes the significance of analyzing these characteristics in conjunction to understand stakeholders better.  The study emphasized the importance of incorporating both top-down and bottom-up approaches when analyzing policy actors involved in policy implementation. Different levels of stakeholders (national, local, and frontline) have varying types of knowledge, interests, and sources of power, which must be considered in the analysis. | | Secondary |  | |

| CMOC-58 | Context | Mechanism | Outcome | Excerpts from Text |
| --- | --- | --- | --- | --- |
|  | Hierarchical structures | Positionality of stakeholders influence their interest in the participation in the collaborative efforts. When few sectors are prioritised in terms of resource allocation other feel left out leading to poor engagement | Lack of coordination among sectors | The study emphasized the importance of assessing power dynamics among stakeholders during policy implementation. Power dynamics encompass various aspects, such as resources, exercise of power, and interactions among stakeholders. The study also highlighted the challenges in assessing stakeholder power, especially in contexts with diverse actors and power structures. Additionally, the study pointed out the significance of incorporating both top-down and bottom-up approaches when analyzing policy actors, recognizing that even frontline workers, often considered to have low power, can collectively wield significant discretionary power that can impact policy outcomes. These findings reinforce the need to consider nuanced power dynamics to better understand the intersectoral collaboration landscape during policy implementation in low- and middle-income countries. |
|  | If hierarchical structures are in place,(C) then the positionality of stakeholders will impact their interest in participating in collaborative efforts.( R )When certain sectors are prioritized, others may feel excluded,(R ) resulting in poor engagement. Additionally, if there is a lack of coordination among sectors, collaborative efforts can be hindered. (O) | | | |
| Relevant IPT(s) | Confirm/Refute/ Refine* | Suggested Revisions* | | Notes* |
| IPT 5 | Confirm |  | |  |

| Author | Year | Publication Type | Aims/Objectives | Setting |
| --- | --- | --- | --- | --- |
| Ahad Bakhtiari | 2022 | Peer reviewed article | The aim of the study is to investigate intersectoral collaboration (ISC) in preventing and controlling noncommunicable diseases (NCDs)-related risk factors in Iran. This investigation involves conducting stakeholder analysis and social network analysis to understand and improve collaboration among governmental stakeholders involved in NCD prevention and control. The study aims to identify the interest, position, and power of decision-making centers related to NCDs, analyze the social network of councils involved, and determine how they influence NCDs and related risk factors. Additionally, the study seeks to identify interventions to enhance intersectoral collaboration in this context. |  |
| Study Participants | Study Design | Findings |  |  |
|  |  |  | | |
| Key messages of the paper | | Contributor |  | |
| The study provides valuable insights into the landscape of intersectoral collaboration in the context of NCD prevention and control in Iran, highlighting the key decision-making centers and proposing interventions to strengthen cooperation and coordination among them. | |  |  | |

| CMOC-59 | Context | Mechanism | Outcome | Excerpts from Text |
| --- | --- | --- | --- | --- |
|  | National health policy | Imbalance in the government vs non-government stakeholder representation leads to skewed distribution of power among govt and non govt actors which affects diversity of perspectives | Biased decision making and poor engagement | The study identifies 113 national collective decision-making centers that have an influence on NCDs and their risk factors. These centers include governmental and non-governmental members. Notably, the majority of members (86%) are from governmental ministries, while only a minority (14%) are non-governmental. This distribution of power within these councils is skewed towards government representation, potentially affecting the diversity of perspectives and interests in decision-making processes. |
|  | If there is an imbalance in the representation of government and non-government stakeholders in the national health policy and structures (C), then there will likely be a skewed distribution of power among government and non-government actors, (R)which can affect the diversity of perspectives. This imbalance can lead to biased decision-making and poor engagement in the policy development process (O). | | | |
| Relevant IPT(s) | Confirm/Refute/ Refine* | Suggested Revisions* | | Notes* |
| IPT 5 | Confirm |  | |  |

| CMOC-60 | Context | Mechanism | Outcome | Excerpts from Text |
| --- | --- | --- | --- | --- |
|  | Hierarchical structures | Supreme Councils for decision making  The concentration of decision making authorities lied in few higher key authorities from the supreme councils, this disempowered the other stake holders | Leading to biased decision making and poor sectoral collaboration | he study evaluates several criteria, including interest, position, power, and influence, to identify key councils in the decision-making process related to NCDs. The Supreme Council for Health and Food Security, Supreme Council for Standards, Supreme Council for Environmental Protection, Supreme Council for Health Insurance, and Supreme Council for Centers of Excellence in Medical Sciences are identified as the most influential. These councils hold significant power and influence, suggesting that the decision-making landscape is concentrated among a few key actors. |
|  | When hierarchical structures exist, particularly involving Supreme Councils for decision-making, (C ) and the concentration of decision-making authorities is primarily held by a few higher key authorities within the supreme councils, then this centralization of power gives a sense of disempowerment among other stakeholders. (R ) This disempowerment may result in biased decision-making and hinder sectoral collaboration. (O ) | | | |
| Relevant IPT(s) | Confirm/Refute/ Refine* | Suggested Revisions* | | Notes* |
| IPT 5 | Refine |  | |  |

| CMOC-61 | Context | Mechanism | Outcome | Excerpts from Text |
| --- | --- | --- | --- | --- |
|  | Existing communication structures | Holding the information within higher authorities and poor transparency in information sharing creates trust issues among other stakeholders leading to feeling of undervalued | This leads to poor collaboration | The study employs network analysis metrics, such as betweenness centrality and eigenvector centrality, to understand the importance of nodes (decision-making centers) in the flow of information. The Supreme Council for Standards and Supreme Council for Health and Food Security emerge as central nodes with high betweenness centrality, indicating their pivotal role in information dissemination. This concentration of influence can impact the sharing of knowledge and communication across different decision-making centers. |
|  | When the existing communication structures are poor (C ) and involve holding information within higher authorities and maintaining poor transparency in information sharing, then this can erode trust (R ) among other stakeholders. These trust issues may lead to stakeholders feeling undervalued, (R ) which, in turn, results in poor collaboration. (O ) | | | |
| Relevant IPT(s) | Confirm/Refute/ Refine* | Suggested Revisions* | | Notes* |
| IPT 5 | Confirm |  | |  |

| Author | Year | Publication Type | Aims/Objectives | Setting |
| --- | --- | --- | --- | --- |
| Karina Kielmann | 2014 | Peer reviewed article | The aim of the study is to examine the roles of tuberculosis health visitors (TB HVs) in mediating working relationships among various stakeholders, including private healthcare providers, program staff, and patients, as part of the Private–Public Mix (PPM) Directly Observed Therapy, Short-Course (DOTS) initiative under India's Revised National Tuberculosis Control Programme (RNTCP) in western Maharashtra. The study seeks to understand how social relations are negotiated among these diverse actors within the PPM-DOTS framework. |  |
| Study Participants | Study Design | Findings |  |  |
| Private practitioners and TB programme coordinators |  | the study sheds light on the often-overlooked social dynamics and power relations within the PPM-DOTS framework in India. It underscores the vital role of TB HVs in mediating these complex relationships and advocates for a more comprehensive approach to accountability that considers both technical and social outcomes of healthcare partnerships. | | |
| Key messages of the paper | | Contributor |  | |
|  | |  |  | |

| CMOC-62 | Context | Mechanism | Outcome | Excerpts from Text |
| --- | --- | --- | --- | --- |
|  | Existing communication structures | Disparities in education qualification and position leading to The TB Health Visitors underestimate themselves to approach medical professional leading to poor communication | This leads to poor collaboration for programme outcome | Unequal Interactions: TB HVs' interactions with PPs are characterized by inequality. PPs, who typically hold higher positions and educational qualifications, are not always receptive to the TB HVs' role in the DOTS program, which involves overseeing TB patients' treatment. This unequal power dynamic hinders effective collaboration. |
|  | If the communication structures are hierarchical (C ) If there are disparities in education qualification and position among the TB Health Visitors (TB HVs), then the TB Health Visitors tend to underestimate themselves (R ) when approaching medical professionals. Consequently, this underestimation results in poor communication between the TB HVs and medical professionals, ultimately leading to poor collaboration and negatively impacting the program's outcome. ( O) | | | |
| Relevant IPT(s) | Confirm/Refute/ Refine* | Suggested Revisions* | | Notes* |
| IPT 5 | Confirm |  | |  |

| CMOC-63 | Context | Mechanism | Outcome | Excerpts from Text |
| --- | --- | --- | --- | --- |
|  | Existing communication structures | Biases and prejudices against the public sector and endure disrespectful treatment towards TB HVs makes them feel not respected leading to poor communication | Poor rapport building | Challenges in Building Rapport: Establishing rapport with PPs and other stakeholders is a complex process that involves negotiating hierarchy, knowledge, and status. TB HVs have to overcome biases and prejudices against the public sector and endure disrespectful treatment, such as being made to wait for long hours. |
|  | In a context where hierarchical communication structure exists (C ) If biases and prejudices against the public sector persist and TB Health Visitors (TB HVs) continue to experience disrespectful treatment, ( R1) then the TB HVs may feel disrespected and undervalued. This lack of respect and poor treatment leads to difficulties in communication, ultimately resulting in poor rapport building between the TB HVs and those who hold biases or treat them disrespectfully. (O ) | | | |
| Relevant IPT(s) | Confirm/Refute/ Refine* | Suggested Revisions* | | Notes* |
| IPT 5 | Refine |  | |  |

| CMOC-64 | Context | Mechanism | Outcome | Excerpts from Text |
| --- | --- | --- | --- | --- |
|  | State health policy framework | Professional disparity and identity lead to confusion among private practitioner with respect to their roles and responsibilities, this makes private practitioners to feel that their work is increases in terms of documentation. This leads to poor engagement | Leading to poor collaboration between public and private sector for the TB programme implementation | Professional Disparities: The professional status of TB HVs, who are non-allopathic practitioners, often limits their interactions with allopathic PPs. Allopathic doctors may resist their roles being reduced to mere paperwork or observation of patients taking medication, creating tensions and challenges in collaboration.  Professional Identity: TB HVs struggle to establish a distinct professional identity. Their affiliations with the DOTS program sometimes lead to confusion among stakeholders about their roles, titles, and responsibilities. This lack of clarity affects their ability to engage effectively with various actors. |
|  | If there is a professional disparity and identity confusion among private practitioners regarding their roles and responsibilities in the State health policy framework, (C ) then private practitioners may perceive an increased workload in terms of documentation. (R ) This perception of increased work can result in poor engagement among private practitioners with public sector. As a consequence, the poor engagement can lead to ineffective collaboration between the public and private sectors in implementing the TB program. (O ) | | | |
| Relevant IPT(s) | Confirm/Refute/ Refine* | Suggested Revisions* | | Notes* |
| IPT 5 | Confirm |  | |  |

| Author | Year | Publication Type | Aims/Objectives | Setting |
| --- | --- | --- | --- | --- |
| Ida Okeyo | 2020 | PR | examining implementation of the first thousand days (FTD) of childhood initiative in the Western Cape province of South Africa. This initiative aims to improve child outcomes through a holistic intersectoral  approach, referred to as nurturing care. | South Africa |
| Study Participants | Study Design | Findings |  |  |
|  |  | . | | |
| Key messages of the paper | | Contributor |  | |
| several power dynamics are evident in the development and implementation of the FTD initiative, including provincial autonomy, bureaucratic authority, shifts in priorities, the influence of global and local factors, the political environment, the role of policy communities, and pre-existing conditions. These power dynamics can affect the collaboration, focus, and success of intersectoral initiatives like the FTD. | |  |  | |

| CMOC-65 | Context | Mechanism | Outcome | Excerpts from Text |
| --- | --- | --- | --- | --- |
|  | Existing Bureaucratic Authority structures | Housing the FTD mandate under the Health Programmes Directorate in the Department of Health reflects bureaucratic power dynamic of health department  This placement concentrated the authority and decision-making within the health sector, potentially limiting the influence of other sectors in intersectoral collaboration. | Poor sectoral engagement | Provincial Autonomy vs. National Influence: The FTD initiative was solely a provincial initiative, led by the provincial health department, without national budgetary support. This indicates a power dynamic where the provincial government asserted its autonomy and leadership in the initiative, potentially impacting the collaboration with national authorities.  Bureaucratic Authority: The decision to house the FTD mandate under the Health Programmes Directorate in the Department of Health reflects a bureaucratic power dynamic. This placement concentrated the authority and decision-making within the health sector, potentially limiting the influence of other sectors in intersectoral collaboration. |
|  | When bureaucratic structures (C ) concentrate authority and decision-making within a particular sector, it leads to limited participation (R ) of other sectors in intersectoral collaboration (O ) | | | |
| Relevant IPT(s) | Confirm/Refute/ Refine* | Suggested Revisions* | | Notes* |
| IPT 5 | Confirm |  | |  |

| CMOC-66 | Context | Mechanism | Outcome | Excerpts from Text |
| --- | --- | --- | --- | --- |
|  | National health policy and global agenda’s | Global agenda and international evidence playing significant role in shaping ISC for FTD this makes sectors to contribute for ISC | Leading to greater degree of collaboration | Global and Local Factors: The influence of global agendas and evidence from international sources played a significant role in shaping the FTD initiative. This reflects a power dynamic where global factors influenced the local agenda. |
|  | If national health policy (C ) aligns with global agendas and international evidence, then sectors are more likely to contribute to intersectoral collaboration (ISC),(R ) resulting in a greater degree of collaboration. (O ) | | | |
| Relevant IPT(s) | Confirm/Refute/ Refine* | Suggested Revisions* | | Notes* |
| IPT 5 | Confirm |  | |  |

| CMOC-67 | Context | Mechanism | Outcome | Excerpts from Text |
| --- | --- | --- | --- | --- |
|  | State health system | Policy communities/groups  Policy community such as PICH act as binding agencies between the sectors this makes stakeholders from other sectors to actively participate where they can openly communicate | Leading to greater degree of collaboration and engagement | Policy Communities: The role of policy communities, such as the PICH group, acted as policy entrepreneurs, advocating for the FTD initiative. These communities exerted influence by shaping the direction of the initiative and engaging with key stakeholders. |
|  | If state health systems (C) actively engage policy communities or groups (R1) like PICH as intermediaries between sectors, it encourages stakeholders from various sectors to participate and communicate openly, (R2) ultimately leading to a greater degree of collaboration and engagement in policy development and implementation. (O) | | | |
| Relevant IPT(s) | Confirm/Refute/ Refine* | Suggested Revisions* | | Notes* |
| IPT 5 | Confirm |  | |  |

| CMOC-68 | Context | Mechanism | Outcome | Excerpts from Text |
| --- | --- | --- | --- | --- |
|  | Existing policy frameworks and | pre-existing conditions and efforts by various actors played a role in paving the way for the initiative, indicating a power dynamic where early actions set the stage for later developments. | Leading to sustainable partnership | Pre-existing Conditions: The text mentions that the soil was "tilled" and the seeds planted before the formal launch of the FTD. This suggests that pre-existing conditions and efforts by various actors played a role in paving the way for the initiative, indicating a power dynamic where early actions set the stage for later developments. |
|  | If existing policy frameworks, ( C) incorporates preparatory efforts (R1) by various actors, lay the foundation for an initiative, this power dynamic of early actions setting the stage can lead to the establishment of sustainable partnerships (R2) in policy development and implementation. (O) | | | |
| Relevant IPT(s) | Confirm/Refute/ Refine* | Suggested Revisions* | | Notes* |
| IPT 5 | Confirm |  | |  |

| CMOC-69 | Context | Mechanism | Outcome | Excerpts from Text |
| --- | --- | --- | --- | --- |
|  | Ambiguity in terms of sectoral roles in the policy | Different stakeholders had varying interpretations of the FTD's objectives and strategies. This ambiguity created challenges in aligning interests and achieving a common agenda | Poor collaboration | Lack of Clarity and Varied Ideas Surrounding the FTD: The text highlights a lack of clarity regarding the FTD initiative's objectives and goals. This lack of clarity is attributed to the FTD representing a time period rather than a specific program or policy. Different stakeholders had varying interpretations of the FTD's objectives and strategies. This ambiguity created challenges in aligning interests and achieving a common agenda. |
|  | If ambiguity exists regarding the roles of different sectors in a policy, (C ) where various stakeholders interpret the objectives and strategies differently, then this lack of clarity can lead to challenges in aligning interests (R )and achieving a common agenda, ultimately resulting in poor collaboration (O ). | | | |
| Relevant IPT(s) | Confirm/Refute/ Refine* | Suggested Revisions* | | Notes* |
| IPT 5 | Confirm |  | |  |

| CMOC-70 | Context | Mechanism | Outcome | Excerpts from Text |
| --- | --- | --- | --- | --- |
|  | Hierarchical and vertical programming | Differing interests, institutional constraints, and a lack of clarity regarding the FTD's goals played role in participation of other key sectors over time leading to lack of interests | Poor implementation | Loss of Momentum and Support: Over time, the FTD initiative lost its intersectoral focus and momentum. This loss of focus is attributed to the divergent interests and a perception that the FTD was primarily a health intervention. The fading political support and the departure of key champions of the initiative also contributed to the loss of momentum.  Resistance from Implementation Actors: Implementation actors, particularly those responsible for delivering services within the health sector, expressed concerns about the feasibility of intersectoral initiatives. They believed that intersectoral activities were intangible and fell outside the boundaries of the health sector's responsibilities. This resistance from implementation actors further highlights the influence of power dynamics in shaping the FTD's trajectory. |
|  | If hierarchical and vertical programming structures (C ) leads to differing interests, institutional constraints, and a lack of clarity regarding the FTD's goals, then this can hinder (R) the participation of other key sectors over time, ultimately resulting in a lack of interest and, consequently, poor implementation. (O ) | | | |
| Relevant IPT(s) | Confirm/Refute/ Refine* | Suggested Revisions* | | Notes* |
| IPT 5 | Confirm |  | |  |

| Author | Year | Publication Type | Aims/Objectives | Setting |
| --- | --- | --- | --- | --- |
| Antonio Paulo Gomes Chiari | 2023 | PR | to analyze the intersectoral collaboration in the social protection network involved in promoting early child growth and development in Brazilian municipalities. |  |
| Study Participants | Study Design | Findings |  |  |
|  |  | Challenges and deficiencies in intersectoral collaboration for child development in Brazilian municipalities, and it underscores the importance of addressing power dynamics and mobilizing resources to improve collaboration in this context. | | |
| Key messages of the paper | | Contributor |  | |
| Need to mobilize actors, resources, management, and communication tools that promote processes of intersegment and enrollment in favor of inter- sectoral collaboration policies and practices for child development. | |  |  | |

| CMOC-71 | Context | Mechanism | Outcome | Excerpts from Text |
| --- | --- | --- | --- | --- |
|  | State health policy frameworks | Scarcity of action by mediators and unused local potentials that limit the local sectors engagement leading to poor collaboration | Poor engagement | Our analysis revealed a rhetorical discussion about intersectoral collaboration but almost no practice. While the findings showed a theoretically receptive speech of the management to external intervention (Projeto Nascente), they also highlighted an irregular enrollment of FHS teams and professionals from other sectors. Our study also identified the manager of a Health Department interfering negatively during the intervention, by asking a doctor not to participate in the training and not encouraging the event. The use of strategies by powerful actors hindered the empower- ment and engagement of local professionals. How to align the interests of the involved actors, including management, for better socialization to sustain the changes aimed at intersectoral collaboration? Current models considered that successful networks of aligned interests were created by recruiting a sufficient body of allies and translating their interests into the thinking and acting that maintained the network |
|  | When state health policy frameworks ( C ) lack a fair representation of all the sectors at different hierarchical levels, few sectors especially at local level feel disempowered (R ) leading to poor communication between the sectors leading to poor engagement (O ) | | | |
| Relevant IPT(s) | Confirm/Refute/ Refine* | Suggested Revisions* | | Notes* |
| IPT 5 | Confirm |  | |  |

| Author | Year | Publication Type | Aims/Objectives | Setting |
| --- | --- | --- | --- | --- |
| Denis Male | 2021 | PR | Understanding difficulties in managing industry engagement across government ministries and in developing effective whole-of- government accountability for tobacco control | Uganda |
| Study Participants | Study Design | Findings |  |  |
| Govt officials | Qualitative | Ambiguity and uncertainty about accountability for Article 5.3 implementation, with policy makers in departments beyond health often uncertain about obligations under the FCTC. Second, we highlight how responsibility for Article 5.3 implementation and the obligations incurred are widely seen as restricted to the Ministry of Health. Third, competing mandates and perceived difficulties in reconciling health goals with economic growth are shown to impact on accountability for tobacco control. Yet, importantly, the data also demonstrate enthusiasm in some unexpected parts of government for actively engaging with Article 5.3 and for promoting greater intersectoral coordination. | | |
| Key messages of the paper | | Contributor |  | |
| This paper demonstrates the intrinsic challenges of developing whole-of-government approaches, highlighting considerable uncertainty and ambiguity among decision makers in Uganda about tobacco control governance. The analysis points to the potential for Uganda’s national coordinating mechanism to help reconcile competing expectations and demonstrate the importance of Article 5.3 beyond health actors. | |  |  | |

| CMOC-72 | Context | Mechanism | Outcome | Excerpts from Text |
| --- | --- | --- | --- | --- |
|  | State health policy structures | Perceived responsibility in the health sectors leading to seeing health dept as primary responsible dept for the implementation of FCTC that makes other sectors to feel disinterested in the collaboration | Leading poor ISC | Perceived Responsibility in the Health Sector: Responsibility for Article 5.3 implementation and related obligations are commonly seen as confined to the Ministry of Health. This concentration of responsibility within one sector can be attributed to power dynamics. The health sector may hold more influence or have stronger advocacy for tobacco control, leading to the assumption that this sector alone should bear the responsibility.  Competing Mandates and Economic Growth: Competing mandates and difficulties in reconciling health goals with economic growth impact accountability for tobacco control. Sectors that are more economically driven may resist or undermine tobacco control efforts to protect economic interests. This resistance can be linked to the power of economic sectors to influence policy decisions.  Enthusiasm for Intersectoral Coordination: Despite the challenges, the data also highlight enthusiasm in unexpected parts of the government for actively engaging with Article 5.3 and promoting greater intersectoral coordination. This enthusiasm may result from power shifts or changes in priorities within certain sectors. |
|  | "If state health policy appoints health sector as primarily responsible for the implementation of FCTC, that leads to other sectors viewing the health department as the primary responsible department, it results in other sectors feeling disinterested in collaboration. This can lead to poor intersectoral collaboration (ISC)." | | | |
| Relevant IPT(s) | Confirm/Refute/ Refine* | Suggested Revisions* | | Notes* |
| IPT 5 | Confirm |  | |  |

| Author | Year | Publication Type | Aims/Objectives | Setting |
| --- | --- | --- | --- | --- |
| Jenny Wagner | 2022 | PR | To understand how integrated partnerships, with shared tasks and accountability across organizations, changed the nature of depression care for older adults. |  |
| Study Participants | Study Design | Findings |  |  |
| Stakeholders | Qual | Partnerships established by the Care Partners Project reshaped late-life depression care in two ways: (1) bidirectional communication across organizations facilitated greater recognition among providers of intersecting medical and social needs associated with late-life depression; and (2) depression care became more coordinated and effective as care teams established or strengthened relationships across organization | | |
| Key messages of the paper | | Contributor |  | |
| The ways cross-organizational health and social care partnerships that move beyond traditional referrals can strengthen late-life depression care and enhance organizational capacities. | |  |  | |

| CMOC-73 | Context | Mechanism | Outcome | Excerpts from Text |
| --- | --- | --- | --- | --- |
|  | Communication structures within programme | If there is platform for facilitating recognition of intersectoral needs this brings sense of shared responsibility among stakeholders leading to open communication and clarity in tasks | Leading to greater ISC | Facilitating Recognition of Intersecting Needs: Bidirectional communication across organizations enabled providers to recognize the interconnection between medical and social needs associated with late-life depression. This recognition likely involved sharing insights, experiences, and information related to patients' conditions and needs. In this way, communication served as a bridge between different providers and organizations, helping them see the bigger picture of patients' needs beyond their specific domains.  Coordinating Care: Effective communication led to the coordination of care, making it more efficient and purposeful. When care teams from different organisations established or strengthened relationships, it suggests that they were able to communicate more effectively with each other. This improved coordination likely resulted in a more comprehensive and holistic approach to late-life depression care. Care providers were able to work together more seamlessly, leveraging their collective expertise and resources. |
|  | “If the state health policy programme (C ) offers a platform facilitating recognition of intersectoral needs within a program, this brings a sense of shared responsibility among stakeholders, (S) leading to open communication and clarity in tasks. (O) This, in turn, leads to greater intersectoral collaboration (ISC)."(O2) | | | |
| Relevant IPT(s) | Confirm/Refute/ Refine* | Suggested Revisions* | | Notes* |
| IPT 5 | Refute |  | |  |
