# Supplementary material for: Power dynamics and intersectoral collaboration for health in low- and middle-income countries: a realist review
Source: Health Policy Plan. 2025 Apr 5;40(6):661–83. doi: 10.1093/heapol/czaf022 (PMC12160828; doi:10.1093/heapol/czaf022)
Supplement: czaf022_Supp [file czaf022_supp.zip › Supplementary File 2.docx]

Supplementary File 2: Demi Regularities

| **Demi Regularity** | **Sub DR** | **Relevant CMOCs** | **Supported studies** |
| --- | --- | --- | --- |
| Coordination and Role Clarity Issues | Overlapping Roles and Responsibilities | CMOC-1, CMOC-2 | Lizah Nyawira et al. |
|  | Redundancy and Inefficiency | CMOC-3, CMOC-4 | Antônio Paulo Gomes Chiari et al. |
|  | Poor Integration and Collaboration | CMOC-1, CMOC-3, CMOC-4 | Anne L. et al. |
| Power Imbalance and Participation | Hierarchical State Power | CMOC-9 | André Janse van Rensburg et al. |
|  |  | CMOC-17 | Denice Kamugumya et al. |
|  |  | CMOC-65 | Ida Okeyo et al. |
|  | Non-Inclusive Policies | CMOC-20, CMOC-21, CMOC-22, CMOC-26 | Denice Kamugumya et al. |
|  | Political Influence and Resource Allocation | CMOC-27, CMOC-28 | F. A. Asaag et al. |
|  |  | CMOC-46 | Santosh R. Pathak et al. |
| Communication and Trust Issues | Communication Structures and Transparency | CMOC-12, CMOC-19, CMOC-30 | Enyi Etiaba et al. |
|  |  | CMOC-38, CMOC-39 | Anne L. et al. |
|  |  | CMOC-61 | Karina Kielmann et al. |
|  | Hierarchical Structures and Power Dynamics | CMOC-32, CMOC-34 | Aloysius Ssennyonjo et al. |
|  |  | CMOC-42 | Anne L. et al. |
|  |  | CMOC-62, CMOC-63 | Karina Kielmann et al. |
|  | Inclusivity and Stakeholder Engagement | CMOC-13 | Enyi Etiaba et al. |
|  |  | CMOC-43 | Anne L. et al. |
|  |  | CMOC-45, CMOC-46 | Santosh R. Pathak et al. |
|  |  | CMOC-69 | Antônio Paulo Gomes Chiari et al. |
|  | Effective Communication and Capacity Building | CMOC-47 | Santosh R. Pathak et al. |
|  |  | CMOC-50 | Sunny S. Kim et al. |
|  |  | CMOC-51, CMOC-52 | Enyi Etiaba et al. |
| Resource Allocation and Support | Favouritism and Inequity | CMOC-20, CMOC-21, CMOC-22 | Denice Kamugumya et al. |
|  | Inadequate Support and Resources | CMOC-16 | Enyi Etiaba et al. |
|  |  | CMOC-24 | F. A. Asaag et al. |
|  | Political and Administrative Influence | CMOC-27 | Anne L. et al. |
|  |  | CMOC-56 | Enyi Etiaba et al. |
| Interpersonal Communication | NA | CMOC-10, CMOC-12, CMOC-16, CMOC-51, CMOC-52 | Enyi Etiaba et al. |
|  |  | CMOC-33 | Aloysius Ssennyonjo et al. |
|  |  | CMOC-49, CMOC-50 | Sunny S. Kim et al. |
|  |  | CMOC-67 | Ida Okeyo et al. |
